# Mechanistic Insights into CYP199A4-Catalyzed α-Hydroxyketone Formation and Hydrogen Bond-Assisted C–C Bond Cleavage Catalyzed by the CYP199A4 F182L Mutant

**DOI:** 10.3390/ijms26041526

**Published:** 2025-02-11

**Authors:** Chang Yuan, Jiaqi Xu, Shun Wang, Ye-Guang Fang, Hongwei Tan

**Affiliations:** 1 Institute of New Materials & Industrial Technologies, Wenzhou University, Wenzhou 325024, China; yuanchang@bnu.edu.cn (C.Y.); shunwang@wzu.edu.cn (S.W.); 2 Key Laboratories of Theoretical and Computational Photochemistry, Ministry of Education, Beijing Normal University, Beijing 100875, China; xu1455410657@163.com

**Keywords:** cytochrome P450 enzymes, lyase reactions, CYP199A4, protein engineering

## Abstract

CYP199A4 is a cytochrome P450 and can catalyze the hydroxylation of 4-propionylbenzoic acid (4-pIBA) to generate α-hydroxyketone with high stereoselectivity. The F182L mutant of CYP199A4 (F182L-CYP199A4) has been shown to support the cleavage of the C–C bond between the carbonyl and hydroxyl groups of α-hydroxyketone, whereas wild-type CYP199A4 cannot. To uncover how the Phe182 regulates substrate reactivity, we conducted classical molecular dynamics (MD) and quantum mechanics/molecular mechanics (QM/MM) MD simulations on these systems. The results predicted that the formation of α-hydroxyketone preferentially led to the (*S*)-enantiomer. Moreover, the findings revealed that the F182L-CYP199A4 facilitated the formation of a hydrogen bond between the α-hydroxyketone and the reactive peroxoanion (POA) species. This interaction stabilized the α-hydroxyketone near POA and promoted the subsequent C–C bond cleavage. The mechanism of α-hydroxyketone formation and the subsequent C–C bond cleavage were elucidated by employing the hybrid density functional theory (DFT). The α-hydroxyketone formation mechanism involved C–H hydroxylation of 4-pIBA with a rate-limiting energy barrier of 17.1 kcal/mol. The C–C bond cleavage of α-hydroxyketone catalyzed by F182L-CYP199A4 occurred via a radical attack mechanism.

## 1. Introduction

Carbon–carbon (C–C) bonds are fundamental chemical bonds in organic compounds. Selectively cleaving C–C bonds and performing molecular editing, such as deleting, inserting, or modifying atoms or functional groups, enables the restructuring of the carbon framework and the rapid construction of unique organic compound structures [1,2]. However, C–C bonds are typically challenging to break without prior activation of the substrate. A common strategy in nature is to couple the cleavage of C–C bonds with oxidation reactions [3,4,5,6]. This process involves functionalizing the target C–C bond to incorporate adjacent structures such as carbonyls, tricarbonyl compounds, diols, or aldehydes (hereafter referred to as the *preactivation* process for C–C bond cleavage), thereby enabling the subsequent cleavage reaction. 

Cytochrome P450 enzymes (CYP450s) are a class of heme-containing enzymes linked to thiols that are capable of catalyzing various reactions, including hydroxylation, epoxidation, and heteroatom oxidation. They are among the most widely used biocatalysts in nature [7,8,9,10,11,12,13]. Due to the requirement for *preactivation* functionalization before C–C bond cleavage and combined with its multifunctional characteristics, CYP450s are ideal catalysts for facilitating C–C bond cleavage reactions.

**Scheme 1:**
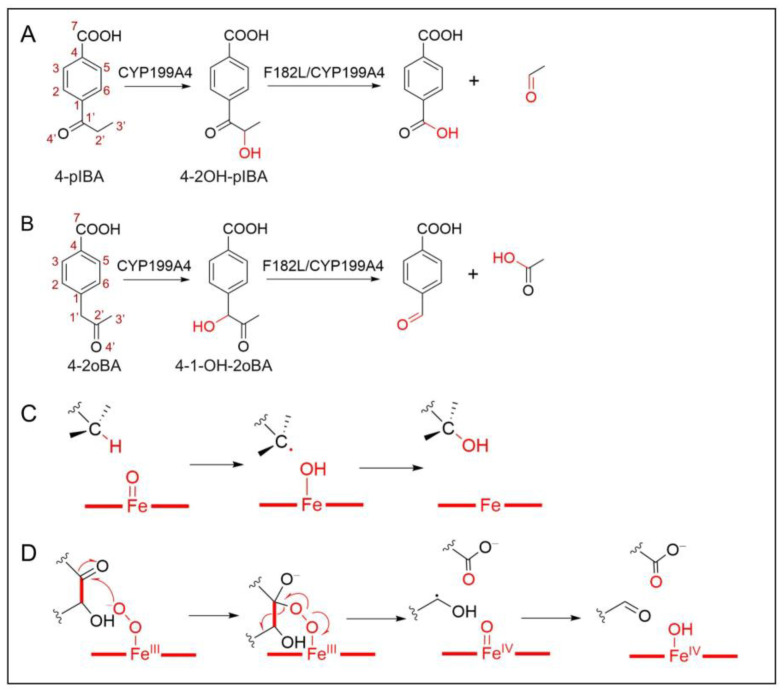
CYP199A4-catalyzed oxidation of 4-propionylbenzoic acid (**A**) and 4-(2-oxalopropyl)-benzoic acid (**B**). Mechanistic hypotheses for (**C**) aliphatic C–H bond hydroxylation and (**D**) C–C bond breakage by cytochrome P450s.

**Figure 1:**
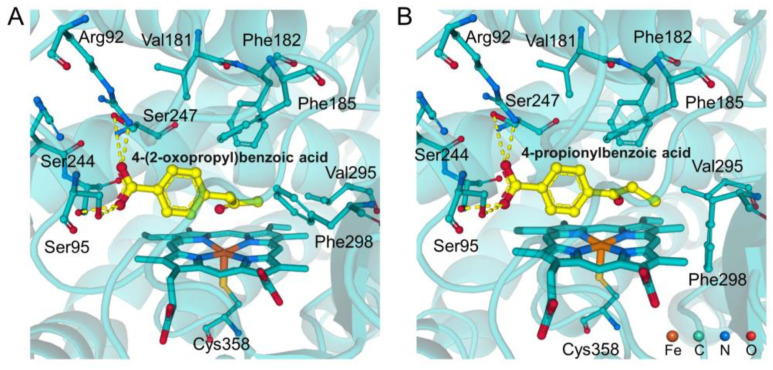
Active sites of CYP199A4 combined with (**A**) 4pIBA (PDB CODE: 8VKF) and (**B**) 4-2oBA (PDB CODE: 8VL0) in the X-ray crystal structures.

The cytochrome P450 CYP199A4 derived from *Rhodopseudomonas palustris HaA199* has been demonstrated to be an ideal model system for expanding the functionality of CYP450s and for catalyst design [14,15,16,17,18]. Its natural catalytic function efficiently oxidizes *para*-substituted benzoic acids, such as 4-methoxybenzoic acid [15,19,20]. Lee et al. reported that wild-type CYP199A4 (WT-CYP199A4) is capable of catalyzing the hydroxylation of the C–H bond at the C2’ position of 4-propionylbenzoic acid (designated as 4pIBA) and the C–H bond at the C1’ position of 4-(2-oxopropyl) benzoic acid (designated as 4-2oBA); this results in the formation of α-hydroxyketone metabolites, namely, 4-(2-hydroxypropionyl) benzoic (designated 4-2OH-pIBA) acid and 4-(1-hydroxy-2-oxopropyl) benzoic acid (designated 4-1-OH-2oBA) (Scheme 1A,B) [21]. The activities catalyzed by CYP199A4 are supported by a class I electron transfer chain, which consists of a [2Fe–2S] ferredoxin (HaPux) and a flavin-dependent ferredoxin reductase (HaPuR), facilitating heme reduction via NADH [17,18,22,23]. Interestingly, as shown in Figure 1, the crystal structures of the WT-CYP199A4/4pIBA and WT-CYP199A4/4-2oBA complexes reveal that the alkyl groups to be hydroxylated of these substrates do not orient toward the active iron; this result contradicts the typically favorable configuration assumed for CYP450-catalyzed aliphatic C–H bond hydroxylation reactions (Scheme 1C) [21]. Recent work by Mokkawes et al. shows that while crystal structures suggest specific catalysis via substrate binding, this is not always the quantum mechanically lowest-energy pathway [24]. The reaction state of substrate in CYP199A4 requires further investigation.

Moreover, the hydroxylation reactions catalyzed by CYP199A4 for 4pIBA and 4-2oBA demonstrated high enantioselectivity; however, the experimental results did not clarify the predominant enantiomer. Using molecular dynamics (MD) simulations, Lee et al. proposed that the oxidation of 4pIBA during the pre-activation reaction would favor the formation of the (*R*)-enantiomer. Nevertheless, the MD simulation results from Lee et al. indicated minimal selectivity preference for one enantiomer (4-2oBA) [21]. The mechanisms by which the substrate undergoes conformational adjustments to achieve highly enantioselective hydroxylation reactions need to be further investigated. Experimental observations indicated that the binding affinities of these two substrates differed by an order of magnitude, and 4pIBA exhibited a tighter binding affinity than 4-2oBA. However, these two substrates differed only at the C=O position; the cause for this substantial impact on binding affinity remains unknown [21]. This insight is needed to guide subsequent substrate design using further detailed analysis.

Lee et al. investigated the potential of CYP199A4 to catalyze the C–C bond cleavage in the substrates 4-(1′-hydroxy-1′-methyl-2′-oxopropyl)benzoic acid (labeled as JCM1) and 4-(1′-oxo-2′-hydroxy-2′-methylpropyl)benzoic acid (labeled as JCM2) [25]. Interestingly, while wild-type CYP199A4 (WT-CYP199A4) was ineffective in catalyzing C–C bond cleavage in these substrates, the F182L–CYP199A4 facilitated successful C–C bond cleavage (Scheme S1). Interestingly, when the methyl group adjacent to the hydroxyl group was removed (4-2OH-pIBA and 4-1-OH-2oBA), WT-CYP199A4 also could not catalyze the C–C bond cleavage, whereas the F182L-CYP199A4 retained this C–C bond cleavage catalytic capability [21]. MD simulations indicated that the bulky side chain of F182 restricted substrate rotation and prevented optimal orientation at the active site. These results indicated that if a substrate capable of unrestricted head rotation could be designed, WT-CYP199A4 might be able to effectively catalyze the sequential hydroxylation reactions and subsequent C–C bond cleavage in carbonyl-containing compounds [21]. However, further research is needed to prove whether this was truly the case.

The catalytic mechanism by which CYP450s mediate C–C bond cleavage has long been of significant interest [2,26,27]. Two primary candidate species are proposed as the key active intermediates in the CYP450s: the iron peroxo anion (designated POA) and the high-valent iron-oxo porphyrin π-cation radical (designated Cpd I) [2,26,27,28,29,30,31,32,33]. Lee et al.’s research group used isotope labeling experiments to confirm that the active oxidizing species in this catalytic cycle was POA [21]. This finding provided critical insight into the reaction mechanism. According to previous studies, CYP450-catalyzed C–C bond cleavage mediated by POA primarily proceeded via a radical pathway (Scheme 1D). The POA species facilitated substrate C–C bond cleavage, generating reactive radical intermediates that could subsequently transform into various oxidized groups, including ketones, carboxyl groups, double bonds, and hydroxyl groups [2,28,29,30]. Previous work by the de Visser group has provided valuable reference models for the CYP199A4 enzyme, offering essential insights to this study [34].

In this study, classical MD simulations and QM/MM MD simulations were employed to predict the regioselective hydroxylation of 4pIBA and 4-2oBA. We determined that the hydroxylation of 4pIBA favors the production of the (*S*)-enantiomer. These simulations indicated that the F182L-CYP199A4 enabled the substrate to approach the POA-oxidizing substance more closely to facilitate the C–C bond cleavage. To further understand the underlying mechanisms, the hybrid density functional theory (DFT) calculations were carried out for both the hydroxylase and lyase reactions. Based on 4pIBA, we designed a reaction process involving consecutive C–H bond hydroxylation and C–C bond cleavage for the 4pIBA lacking the terminal methyl group, and this reaction was catalyzed by CYP199A4. The catalytic mechanisms of CYP199A4 in these reactions were comprehensively examined and could be used to facilitate new avenues for related research and applications.

## 2. Results and Discussion

### 2.1. Target Systems of the C–H Bond Hydroxylation

**Figure 2:**
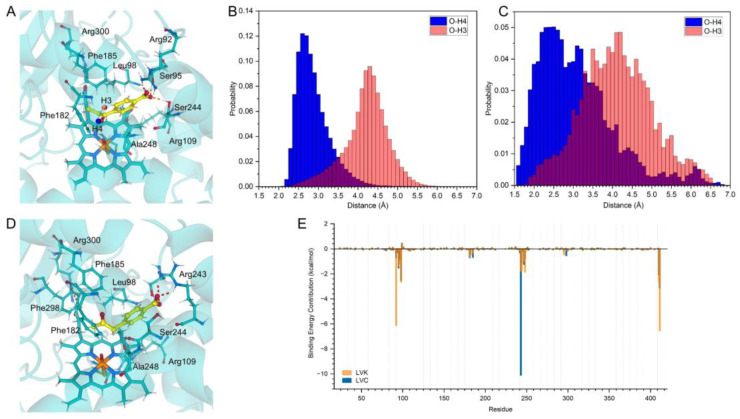
(**A**) MD-simulated complexes of CYP199A4/Cpd I/4-pIBA. Distance distributions of the oxo of Cpd I to the site of H3 or H4 in the classical MD simulation (**B**) and in the QM/MM MD simulation (**C**). (**D**) MD-simulated complexes of CYP199A4/Cpd I/4-2oIBA. (**E**) MMPBSA energy decomposition of 4-pIBA or 4-2oIBA bound to CYP199A4.

To investigate the enantioselectivity of CYP199A4-catalyzed substrate hydroxylation, we constructed initial CYP199A4/Cpd I/4-pIBA and CYP199A4/Cpd I/4-2oIBA complex models in conf-1 (maintain the binding mode of the substrate in the crystal structure) and conf-2 (shown in Figure S1, consistent with the results from the MD simulations by Bell et al.), respectively, as described in the Models and Methods section. Classical MD simulations of 500 ns were performed on these complexes. Interestingly, during the simulations of conf-2 for both 4-pIBA and 4-2oIBA, the C–H bond rapidly deviated from the set position and eventually relaxed back to the conformation in conf-1; these results indicated that the complexes of CYP199A4/Cpd I/4-pIBA and CYP199A4/Cpd I/4-2oIBA in conf-2 were not stable in the presence of Cpd I. Therefore, only the CYP199A4/Cpd I/4-pIBA and CYP199A4/Cpd I/4-2oIBA complexes in conf-1 were used for further evaluation. Results of the root-mean-square-deviation (RMSD) during the 500 ns MD simulations indicate both complexes achieved stable conformations (Figure S2). Figure 2A,D show the representative structures of the two complexes obtained through cluster analysis of the 500 ns MD simulation trajectories. It is apparent that the substrates deviated from the Fe center, and space was created for the oxo group of Cpd I in both complexes.

In the CYP199A4/Cpd I/4-pIBA complex, the heme is anchored via a salt bridge between its carboxylate group and Arg109 and Arg300. The 4-pIBA molecule binds within a pocket formed by Arg92, Ser95, Leu98, Phe182, Phe185, Ser244, and Ala248. The carboxylate head of 4-pIBA engages in a salt bridge interaction with Arg92 and forms hydrogen bonds with the hydroxyl groups of Ser95 and Ser244. The phenyl portion of 4-pIBA hydrophobically interacts with Leu98, Phe185, and Ala248, whereas the ethyl end of 4-pIBA is positioned away from the active Fe-O center. To assess enantioselectivity of the 4-pIBA hydroxylation, we measured the distances between the oxo atom and the two hydrogen atoms (H3 and H4) on C2’ over the 500 ns simulation, as shown in Figure 2B. Nevertheless, our results differed from those of Bell et al. Specifically [21], in our study, the H4 atom was more favorable for abstraction by a central metal than the H3 atom; these results indicated that CYP199A4 preferentially catalyzed the formation of the (S)-enantiomer of 4-pIBA rather than the (R)-enantiomer predicted by Bell et al. To further verify our conclusion, we conducted an additional 20 ns QM/MM MD simulation based on the initial conf-1 of CYP199A4/Cpd I/4-pIBA; these results also supported our MD findings (Figure 2C). In summary, our results indicated that CYP199A4 favored the formation of the (*S*)-enantiomer in the hydroxylation of 4-pIBA. In the following sections, we compare the reaction potential energy surfaces for the formation of both enantiomers to further substantiate this deduction, which will be discussed in detail.

The representative structure of the CYP199A4/Cpd I/4-2oIBA complex after 500 ns of MD simulation is shown in Figure 2D. The binding site of 4-2oIBA is largely consistent with that of 4-pIBA, but slight differences are observed in the interactions with the active site of CYP199A4 due to the functional group variation. Compared with those of 4-pIBA, the carbonyl and methylene groups at the 1’ and 2’ positions of 4-2oIBA exchange. In 4-2oIBA, the 1’ position contains a methylene group, and the 2’ position contains a carbonyl group. The sp3 hybridization at the 1’ carbon causes the 2’ carbonyl to approach Phe185, weakening their hydrophobic interaction. Moreover, the 1’ methylene strengthens the interaction with Phe182 on the opposite side. This subtle difference shifts the salt bridge interaction of the carboxylate head from Arg243 to Arg92 and causes a binding energy difference. Our MMPBSA calculations show a binding energy of 28 kcal/mol for 4-pIBA with CYP199A4, whereas it is 22 kcal/mol for 4-2oIBA; these results align with the experimental observations. The energy decomposition analysis (Figure 2E) further corroborates these findings.

### 2.2. Mechanism of α-Hydroxyketone Formation from 4-pIBA Catalyzed by CYP199A4

**Figure 3:**
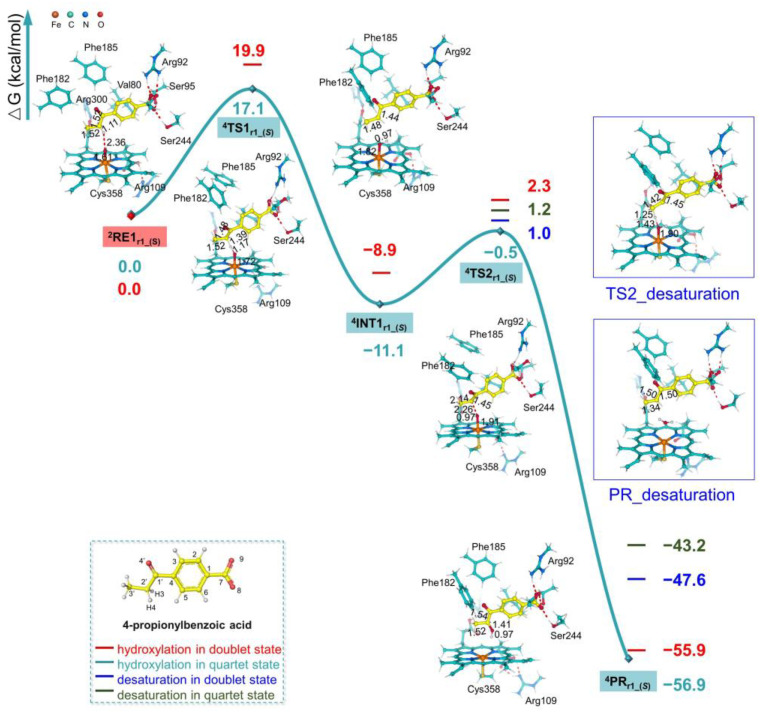
Energy profile of the CYP199A4-catalyzed C–H bond hydroxylation and desaturation reactions of 4-pIBA. The key structural information of the stationary points along the reaction pathway is shown (Å).

Since the formation of α-hydroxyketone from 4-pIBA and 4-2oIBA catalyzed by CYP199A4 proceeds through fundamentally similar mechanisms, we focus exclusively on 4-pIBA to investigate the underlying reaction mechanism. Based on the representative snapshots extracted from the MD trajectories of the CYP199A4/Cpd I/4-pIBA complex, we constructed models for the reactant state of this system. Considering the electronic configuration of Cpd I, we investigated the C2’–H4 hydroxylation reaction pathways on the doublet (S = 1/2) and quartet (S = 3/2) potential energy surfaces. The calculation results indicated that the energies of reactants on the doublet and quartet surfaces are quite close to 0.3 kcal/mol, which was the same as the early work [35,36,37,38,39,40,41,42]. The quartet surface exhibited lower energy barriers. Therefore, the details of the C2’–H4 bond hydroxylation of 4-pIBA by CYP199A4 to produce the (*S*)-enantiomer in the quartet state are subsequently described. As illustrated in Figure 3, the calculations indicated that the C2’–H4 hydroxylation process involved two key steps: (1) hydrogen atom H4 transfer (HAT) from the target carbon atom to the oxo of Cpd I, followed by (2) hydroxyl OH4 rebound to the C2’-centered radical of the substrate. The key structural information and Mulliken Spin Densities of the stationary points along the CYP199A4-catalyzed C-H bond hydroxylation in doublet state are shown in Figure S3 and Table S1, respectively.

In the initial reactant state ^2^RE1_r1_(*S*)_ of the CYP199A4/Cpd I/4-pIBA system, the central metal adopts an octahedral coordination structure formed by coordination with the four nitrogen atoms of the porphyrin ring, the proximal axial ligand Cys358 thiolates, and the distal axial ligand oxo. The C2’–H4 bond of 4-pIBA is oriented toward the Fe(IV)=O group of Cpd I, with an H4···O distance of 2.36 Å and a ∠C14-H4-O angle of 158.2° (Figure 3). As shown in Table 1, the Mulliken spin density on Fe(IV)=O in ^2^RE1_r1_(*S*)_ is +2.09, whereas the total Mulliken spin density on the porphyrin ring and the proximal Cys358 ligand (labeled (Por^+^-Cys^−^)·) is -1.10. Spin analysis reveals that two unpaired α electrons occupy the two π* orbitals of Fe(IV)=O in ^2^RE1_r1_(*S*)_, with an additional unpaired β electron on the porphyrin ring and proximal Cys358 ligand. In typical cytochrome P450 Cpd I species, the porphyrin ring exists as a cationic radical; however, in ^2^RE1_r1_(*S*)_, the total spin density on the porphyrin ring is approximately -0.25 and is caused by the oxidation of the anionic Cys358 ligand by the porphyrin ring in ^2^RE1_r1_(*S*)_.

The first reaction step from ^2^RE1_r1_(*S*)_ involves hydrogen atom abstraction by Cpd I from the C2’ atom, which requires an energy barrier of 17.1 kcal/mol on the quartet potential energy surface to reach the transition state ^4^TS1_r1_(*S*)_. In the optimized ^4^TS1_r1_(*S*)_ structure, the C2’–H4···O alignment becomes nearly collinear, with a C2’–H4 bond length of 1.39 Å and O···H4 distance of 1.17 Å. This linearity is consistent with the expected geometry for efficient hydrogen abstraction, and is similar to the previous studies [40,43,44,45,46,47,48]. As the C2’–H4 bond elongates, the spin density on the substrate molecule increases from 0.00 in ^2^RE1_r1_(*S*)_ to +0.47, whereas the spin density on Fe(IV)=O decreases from +2.10 to +1.70, with no unpaired electron remaining on H4. The change in the Mulliken spin density indicates the transfer of a β-spin electron from Fe=O to the (Por^+^-Cys^−^) fragment in this step; these results confirm that this reaction proceeds via a HAT mechanism.

After the HAT step, intermediate ^4^INT1_r1_(*S*)_ is formed. In ^4^INT1_r1_(*S*)_, the Mulliken spin density on C2’ is +0.72, indicating a radical character. The Mulliken spin densities on the central iron and oxygen atoms are +1.78 and +0.16, respectively; these correspond to an intermediate-spin Fe(IV)-O state. The β electron carried by the hydrogen atom pairs with the α electron on the oxygen atom to form an O–H4 bond with a bond length of 0.97 Å. This pairing disrupts the collinear alignment of the C2’–H4···O structure and weakens the coordination between oxygen and Fe, as reflected by an increase in the Fe–O bond length of 0.11 Å compared with that of ^2^RE1_r1_(*S*)_.

As the Fe–O distance further increases to 1.91 Å, the OH4 group dissociates from the metal center, reaches the transition state ^4^TS2_r1_(*S*),_ and then rebounds to the C2’ radical of the substrate. Upon crossing ^4^TS2_r1_(*S*)_, the Fe–O bond is completely broken, and the hydroxylated product ^4^P1_r1_(*S*)_ is formed at the C2’ position. In ^4^P1_r1_(*S*)_, the spin density on OH4 disappears, and the spin density at the Fe center increases from +1.78 in ^4^INT1_r1_(*S*)_ to +2.56 in ^4^P1_r1_(*S*)_. These results indicate that during the attack of the OH4 on C2’, a portion of the β electron density from the Fe center transfers to the (Por^+^-Cys^−^)· group. The entire hydroxyl rebound process requires a local barrier of 19.9 kcal/mol.

In addition to generating the (*S*)-enantiomer of the hydroxylation product, we also constructed the reactants for the (*R*)-type hydroxylation reaction and computed the reaction pathway (Figure S4). The details are shown in the Figure S5. The results indicated that the rate-limiting step was the HAT process, with a higher energy barrier of 22.1 kcal/mol. This value represented an increase of 13.2 kcal/mol compared with the local energy barrier associated with the (*S*)-configuration. This indicates that the hydroxylation of the C3’–H bond of 4-pIBA by CYP199A4 to generate the (*S*)-α-hydroxyketone is an energetically unfavorable process. Therefore, during the reaction process of the CYP199A4-catalyzed hydroxylation of 4-pIBA, the (*S*)-isomer was more likely to be formed.

From ^4^INT1_r1_(*S*)_, the desaturation mechanism, where two successive hydrogen atom abstractions lead to the formation of a ketone and water [49,50,51], was also investigated. As shown in Figure 2, the calculations indicate that the energy barrier for the desaturation reaction is very close to that of the hydroxyl rebound step, with the desaturation barrier being slightly higher by 1.5 kcal/mol. Additionally, the desaturation product is 9.3 kcal/mol less exothermic compared to the C–H bond hydroxylation product. Based on the reaction, while the hydroxyl rebound leading to the C–H bond hydroxylation product is more favorable, the desaturation reaction represents a competitive side reaction.

### 2.3. Blocking Mechanism of Phe182 in (S)-4-2OH-pIBA C–C Cleavage Reactions by CYP199A4

**Figure 4:**
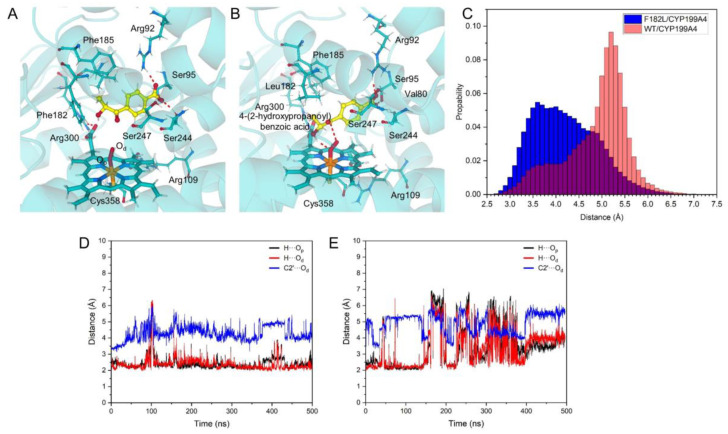
MD simulated complexes of WT-CYP199A4/Cpd I/4-2OH-pIBA (**A**) and F182L-CYP199A4/Cpd I/4-2OH-pIBA (**B**). (**C**) Distance distributions of the O_d_ to the C1’ atom in WT-CYP199A4 and F182L-CYP199A4 complexes, respectively. The distance of the POA to the 4-2OH-pIBA in F182L-CYP199A4 (**D**) and WT-CYP199A4 (**E**) complexes, respectively.

Using isotope labeling experiments, the Lee group confirmed that the active oxidant in this catalytic cycle was the POA [21]. This discovery provided critical insights for further exploration of the reaction mechanism. To investigate the impact of Phe182 on the CYP199A4-catalyzed C1’–C2’ bond cleavage of (*S*)-4-2OH-pIBA, the complexes of WT-CYP199A4 and F182L-CYP199A4 containing POA with 4-2OH-pIBA were constructed, and then, 500 ns MD simulations were performed for each complex. Figure 4A,B shows representative structures of the WT-CYP199A4/POA/4-2OH-pIBA and F182L-CYP199A4/POA/4-2OH-pIBA complexes after 500 ns MD simulation. In both complexes, 4-2OH-pIBA moves away from the central metal, thereby creating space for dioxygen binding. In the F182L-CYP199A4/4-2OH-pIBA complex, 4-2OH-pIBA remains bound within the reactive distance. However, 4-2OH-pIBA is far from the active POA species in WT-CYP199A4; this result is consistent with those from Lee et al. [21]. The variation in the distance of C1’ atom and the distal oxygen atom (O_d_) of POA during the simulation time further supports this result (Figure 4C). Analysis of the trajectories reveals that the hydroxyl group at the C2’ position of 4-2OH-pIBA can form a hydrogen bond with the POA species in both complexes. The distance between the hydroxyl hydrogen and POA is positively correlated with the distance between O···C1’. Specifically, when the hydrogen bond distance between the hydroxyl group and POA is within the optimal hydrogen bonding range, the O_d_···C1’ distance is shorter. These results indicate that the hydrogen bond between the hydroxyl group and POA stabilizes the substrate at the active site and is corroborated by the distance data (Figure 4D,E). However, in WT-CYP199A4, the large hydrophobic side chain of Phe182 obstructs the hydrogen bond interaction between the hydroxyl group and POA. As a result, in WT-CYP199A4, 4-2OH-pIBA is positioned away from the POA, preventing the reaction from occurring.

Additionally, a 200 ns constrained MD simulation of WT-CYP199A4 bound to 4-2OH-pIBA was performed by restricting the O_d_···C1’ distance to 3.5 Å. Then, the binding energies were decomposed for the substrate in its stable state. Interestingly, when the substrate remained close to the central metal, the contribution of Phe182 to the binding energy was positive; these results indicated that the presence of Phe182 was unfavorable for the binding of the hydroxylation intermediate.

### 2.4. C–C Bond Cleavage Mechanism of 4-2OH-pIBA by CYP199A4

**Figure 5:**
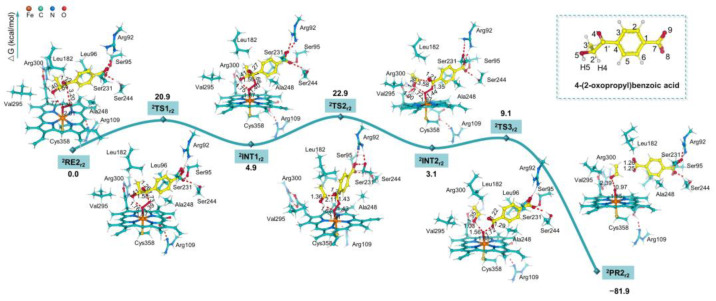
Energy profile of the CYP199A4-catalyzed C–C bond cleavage of 4-2OH-pIBA. The key structural information of the stationary points along the reaction pathway in the doublet state is shown (Å).

Based on prior studies, CYP450s-catalyzed C–C bond cleavage mediated by POA primarily occurs via a radical pathway (Scheme 1D). The POA species facilitate the substrate’s C–C bond cleavage, generating reactive radical intermediates that can subsequently transform into various oxidized functional groups, including ketones, carboxyl groups, double bonds, and hydroxyl groups [2,28,29,30]. Building upon the existing experimental results and previous studies, the detailed potential reaction pathways for the C–C bond cleavage of 4-2OH-pIBA catalyzed by CYP199A4 were verified. Figure 5 shows the potential energy profile and the key stationary points involved in the process. The reaction proceeded in the doublet ground state. In the following sections, we focus on the doublet state to elucidate the reaction mechanism in detail. The Mulliken spin population and Mulliken charges of the stationary points along the reaction path of the CYP199A4-catalyzed C–C bond cleavage of 4-2OH-pIBA in doublet is shown in Table 2.

After the formation of ^2^RE2_r2_, the electrons are transferred from the ferrous ions to dioxygen. Simultaneously, upon receiving an electron from the external environment, the POA species is generated. The C2’–OH group of the substrate 4-2OH-pIBA points toward the proximal oxygen atom (O_p_) of the POA and forms a hydrogen bond interaction with it (H···O_p_ = 1.77 Å). The O_d_ atom is positioned beneath the C1’ atom in a favorable orientation for the subsequent reaction.

The attack of the O_d_ atom on the C1’ atom represents the initial step in CYP-catalyzed C1’–C2’ bond cleavage mediated by POA through a transition state (^2^TS12_r2_). As shown in Figure 5, this step requires a barrier of 20.9 kcal/mol (^2^RE2_r2_→^2^TS12_r2_), and the resulting oxygen-bridged intermediate (^2^INT1_r2_) is 4.9 kcal/mol higher than ^2^RE2_r2_. During this process, the C1’ atom switches from *sp^2^* to *sp^3^* hybridization; this causes the C1’=O group to deviate from the phenyl plane, disrupts the original conjugation, and accounts for the high energy. Additionally, the formation of the C1’–O_d_ bond weakens the C1’–C2’ bond, causing the bond length to increase from 1.54 Å to 1.58 Å. In ^2^INT1_r2_, the hydrogen bond between OH and O_p_ is still maintained.

In the subsequent reaction, the C1’–C2’ bond is further weakened and overcomes an energy barrier of 22.9 kcal/mol; this leads to the bond cleavage and the formation of the intermediate ^2^INT2_r2_ and is exothermic by 1.8 kcal/mol. At this stage, the spin populations on C1’ and C2’ are -0.85 and 0.00, respectively. The spin on the central metal increases from 0.82 in ^2^INT1_r2_ to 1.07 in ^2^INT2_r2_; these results indicate that the homolytic cleavage of the C1’–C2’ bond initially occurs and is immediately followed by the α electron on C2’ being partially transferred to the central metal and the substrate.

**Scheme 2:**
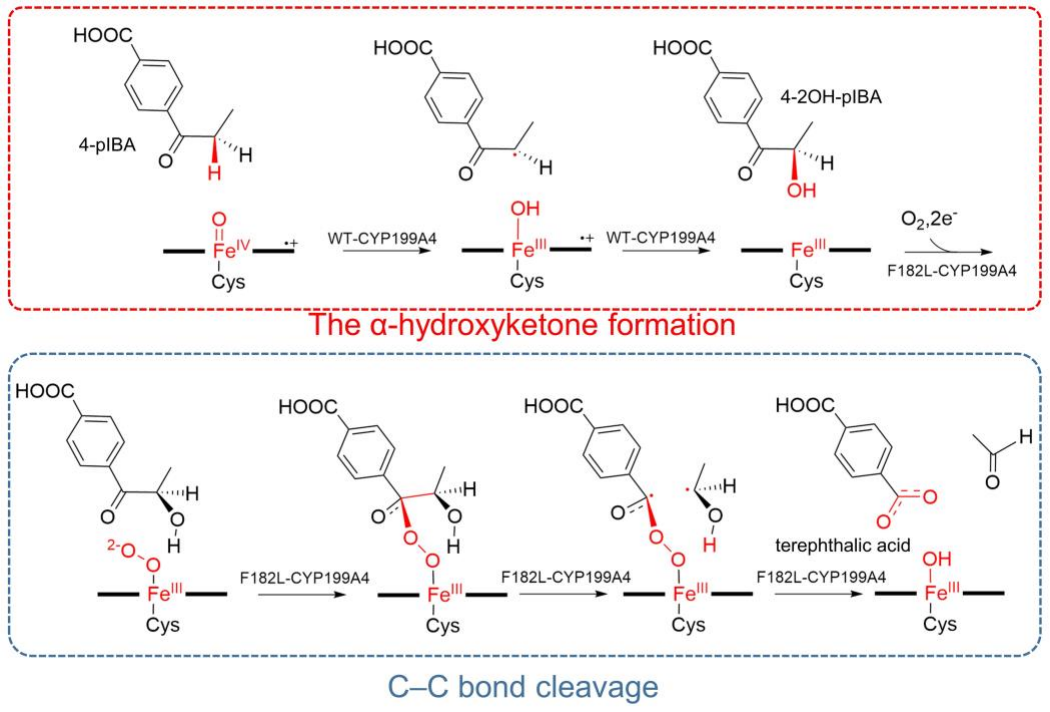
The catalytic conversion of 4-pIBA to terephthalic acid via intermediate 4-2OH-pIBA, with WT-CYP199A4 and F182L-CYP199A4 acting as sequential catalysts in the reaction process.

In the subsequent steps of the reaction, the O_p_–O_d_ bond undergoes cleavage, and the hydrogen atom of the hydroxyl group of 4-2OH-pIBA transfers to the O_p_ atom, completing the reaction process. The quenching of the radicals results in a strongly exothermic reaction with a value of 81.6 kcal/mol. The rate-determining step of the entire reaction is the C1’–C2’ bond cleavage, with an energy barrier of 22.9 kcal/mol. The general reaction is shown in Scheme 2.

### 2.5. Substrate Design for the WT-CYP199A4 Catalyzed C–C Bond Cleavage

Lee et al. investigated the catalysis of JCM2 and 4-pIBA by CYP199A4 [21,25]. They reported that C–C bond cleavage reactions in WT-CYP199A4 could not occur in either JCM2 or 4-pIBA but could be achieved only when Phe182 of CYP199A4 was mutated to Leu182. In the previous section, the mechanism by which F182L-CYP199A4 catalyzed substrate C1’–C2’ bond cleavage indicated that reaction initiation involved the terminal O_d_ atom of POA attacking the carbonyl carbon of the substrate. MD simulations further demonstrated that in WT-CYP199A4, Phe182 prevented the C1’–C2’ bond cleavage of the substrate owing to steric hindrance. The bulky phenyl group of Phe182 and the ethyl group on the substrate were the primary factors restricting this flipping motion. These results led to our hypothesis that consecutive C–H hydroxylation and C1’–C2’ bond cleavage without CYP199A4 mutation could be achieved by reducing the steric hindrance around the substrate’s tail group. Based on this hypothesis, we modified 4-pIBA by substituting the tail methyl group with hydrogen atoms, resulting in new molecules, named 4-pIBA_no_Me_.

To evaluate the feasibility of the initial hydroxylation reaction, the initial WT-CYP199A4/Cpd I/4-pIBA_no_Me_ complex was constructed, and then, 500 ns MD simulations were performed. Olsen’s criterion for the hydroxylase transition state was applied to consider the bond distance between the oxo of Cpd I and the aliphatic hydrogen (*d1*) and the angle (*θ1*) defined by the C1’-H-oxo [26]. The following reaction conditions needed to be met: *d1* ≤ (1.20 + 2) Å and *θ1* ≥ 125°. The representative structure of the CYP199A4/Cpd I/4-pIBA_no_Me_ complex is shown in Figure S5A; analysis of the MD trajectories revealed that in 42.6% of the frames, 4-2OH-pIBA_no_Me_ met the hydrogen abstraction criteria at the hydroxylation site. These MD data indicated that WT-CYP199A4 could catalyze the C–H hydroxylation of 4-pIBA_no_Me_; thus, these results provide a basis for the subsequent C1’–C2’ bond cleavage reaction.

Based on the MD simulations of the WT-CYP199A4/Cpd I/4-pIBA_no_Me_ complex, a complex of hydroxylated 4-2OH-pIBA_no_Me_ (labeled 4-2OH-pIBA_no_Me_) bound with WT-CYP199A4/POA (CYP199A4/POA/4-2OH-pIBA_no_Me_) was constructed, and 500 ns MD simulations were conducted on these structures. Olsen’s criterion for a six-membered cleavage transition state was used to assess whether WT-CYP199A4 could further oxidize α-hydroxyketone [26]. Here, the distance between the O_p_ atom and the hydroxyl hydrogen of 4-2OH-pIBA_no_Me_ is *d1*, the distance between the O_d_ and carbonyl carbon is *d2*, and the angles formed by O_p_-H-O and C1’-O_d_-O_p_ are defined as *σ* and *ρ*, respectively. Sampling the trajectory every 100 ps over the 500 ns simulation yielded 50,000 frames. The following C1’–C2’ bond cleavage conditions needed to be met: *d1* ≤ 3.62 Å, *d2* ≤ 3.47 Å, *σ* ≤ 156°, and *ρ* ≥ 112°. Analysis revealed fewer frames satisfying the transition state criteria for C1’–C2’ bond cleavage in the 500 ns MD simulations of the CYP199A4/POA/4-2OH-pIBA_no_Me_ complex (Figure S5). Specifically, CYP199A4 was unable to catalyze the continuous hydroxylation of the C–H bond and subsequent C–C bond cleavage of the substrate 4-pIBA_no_Me_. Certainly, further experimental verification is needed. Based on the analysis of the trajectories, although the removal of the terminal methyl group reduced the steric hindrance between the substrate and the hydrophobic side chain of Phe182, the flexible terminal group of 4-2OH-pIBA_no_Me_ enabled the hydroxyl group to rotate around its C1’–C2’ single bond and positioned it away from POA. As a result, it did not form a hydrogen bond interaction with POA. Consequently, the substrate was not stabilized at the active site but instead moved toward Arg92 and Arg243. This further supported the role of the hydrogen bonding interaction between the hydroxyl group and POA in stabilizing the substrate.

## 3. Materials and Methods

### 3.1. Classical MD Simulations

Due to the C–H bonds of the two substrates in the crystal structures of CYP199A4/4-pIBA (PDB CODE: 8VKF) and CYP199A4/4-2oIBA (PDB CODE: 8VL0) [21] are far from the active Fe(IV)=O center, CYP199A4 is unable to complete the hydroxylation of the C–H bonds of the two substrates based on these configurations. To obtain reasonable complexes for the hydroxylation of the C–H bonds in 4-pIBA and 4-2oIBA by CYP199A4, we constructed two conformations for each of the two substrates. In the first conformation (conf-1), based on the crystal structure, the oxygen atom along the central metal axial direction was added to construct the Cpd I model. In the second conformation (conf-2), on the basis of the conf-1, the C–H bond involved in the reaction was twisted to face the active Fe(IV)=O center. In the C–C bond cleavage reaction, Lee et al. confirmed that the active oxidant in this catalytic cycle was the POA through isotope labeling experiments [21]. Therefore, POA was used to replace the Cpd I model to obtain the initial complex model for the C–C bond cleavage reaction. In accordance with the pH environment of the experiment, the PROPKA program [52] was used to add the missing hydrogen atoms of the amino acids. All Glu and Asp residues existed in a deprotonated state, whereas Arg and Lys residues were protonated. Regarding His residues, the δ-nitrogen atoms of His20, His65, His105, His173, His202, and His356 were protonated. In contrast, the ε-nitrogen atoms of His225 and His348 were protonated.

For the MD simulations, the AMBER ff14SB force field [53] parameter was first applied to the standard residues in the complexes. The substrates 4-pIBA and 4-2oIBA were parameterized using the Antechamber module [54,55] based on the General AMBER Force Field (GAFF) [56]. The force field parameters of Cpd I and the axial ligand Cys358 were adopted from the results of previous studies [57]. To simulate the real reaction environment, all CYP199A4/4-pIBA and CYP199A4/4-2oIBA complexes were dissolved in TIP3P water boxes [58]. The minimum distance between the boundary of the protein and the edge of the water box was set to 10 Å, leading to a 90.0 × 90.0 × 90.0 cube box. The negative charges of the system were neutralized with Na⁺. Each complex then underwent a three-step energy minimization process to gradually release the tension of the hydrogen atoms, solvent molecules, and entire system. After energy minimization, these systems were heated to 300 K and preequilibrated for 300 ps. Finally, an NPT ensemble was used to conduct a 500 ns MD simulation of these systems. During the simulation, the cutoff distance for nonbonding interactions was 10.0 Å, and the simulation step size was 2 fs. All of the above molecular dynamics simulation operations were completed using AMBER 14 [59] software. To investigate the binding ability of the substrates to CYP199A4, the binding free energies of these complexes were determined separately using the molecular mechanics Poisson–Boltzmann surface area (MM-PBSA) [60,61]. For all systems, after the MD simulation reached equilibrium, the last 50 ns of the trajectory were selected, and then 500 snapshots were extracted at 100 ps intervals for energy calculations.

### 3.2. QM/MM MD

To further obtain stable complexes, the CYP199A4/4-pIBA and CYP199A4/4-2oIBA without MD simulation were extracted to establish the initial models for QM/MM MD. The QM regions in the models include (1) Cpd I; (2) the proximal axial ligand cysteine (Cys358) of the central metal; (3) the substrates 4-pIBA or 4-2oIBA; and (4) the key residues (Val80, Arg92, Ser95, Arg109, Phe182, Phe185, Ser244 and Arg300) that form intermolecular interactions with the substrates. During the process of QM/MM MD, the atoms in the MM region beyond 6 Å from the QM region were frozen. The QM–MM boundaries were cut in the C_α_-C of the residue’s backbone.

For the QM region, the exchange and correlation interactions of electrons were modeled using the Becke–Lee–Yang–Parr functional with Grimme’s dispersion correction (BLYP-D3) [62,63]. Goedecker–Teter–Hutter (GTH) pseudopotentials were employed to describe the core electrons. Energy cutoffs were set at 300 Ry for the plane-wave basis set and 40 Ry for the Gaussian basis set. In the MM region, the force field parameters for the standard residues in the complexes as well as other components were consistent with those applied in the classical MD simulations. The electrostatic coupling between the QM and MM regions was calculated using a real-space multigrid technique [64]. All QM/MM simulations were carried out in the canonical ensemble (NVT) with a time step of 1 fs, and the temperature was maintained at 300 K using a Nosé–Hoover chain thermostat. All QM/MM simulations were performed using the CP2K 2022 package [65] interfaced with Plumed 2.8 software [66].

### 3.3. DFT Calculations

Based on the results of the QM/MM MD and MD simulations, representative snapshots were selected to construct the initial models for the DFT calculations. Based on the research of Visser and Himo et al. [34,42,47,67,68,69], the cluster models were consistent with the QM regions in the QM/MM MD. Truncation was performed at the α-C atoms of the residues, and the truncated sites were saturated with H atoms. The α-C atoms at the truncation sites were fixed during the optimization process.

Since previous studies have verified that the high-precision calculation results of the unrestricted hybrid density functional method UB3LYP [36,70] for the cytochrome P450 enzyme system are in good agreement with the experimental results [46,71,72,73], we selected the UB3LYP functional and the def2-SVP basis set [74] to explore the catalytic reaction mechanism. The intrinsic reaction coordinate (IRC) and harmonic vibrational frequency calculations were carried out at the same theoretical optimization level to confirm the correctness of the transition states. Based on the calculation results of the frequency analysis, we also obtained the zero-point energy (ZPE) and entropy correction values. The dispersion interaction was evaluated using Grimme’s D3 [63] method. To obtain high-precision reaction free energy, the optimized structures were used with a large basis set (def2-TZVP) [75] to obtain a more accurate energy, and the final reaction Gibbs free energy was obtained by combining entropy correction, van der Waals correction, and zero-point energy correction. All the DFT calculations mentioned above were performed using the Gaussian09 software package [76].

## 4. Conclusions

In this study, the mechanisms of 4-pIBA hydroxylation to the α-hydroxyketone intermediate, catalyzed by WT-CYP199A4, and the subsequent C-C bond cleavage catalyzed by its F182L mutant, have been elucidated using classical MD simulations, QM/MM MD simulations, and DFT calculations. The results provide a detailed understanding of the reaction mechanisms and the key structural and catalytic factors influencing these two steps.

The generation of the α-hydroxyketone intermediate is achieved by the hydroxylation of the aliphatic C–H bond of 4-pIBA. Both classical and QM/MM MD simulations show that, upon introduction of Cpd I, the C2’–H4 bond of 4-pIBA is more likely to orient toward the active Fe=O species, resulting in the formation of the (*S*)-enantiomer of the α-hydroxyketone intermediate. This is slightly different from previous studies. DFT calculations show that the hydrogen atom abstraction is the rate-determining step in this α-hydroxyketone formation step. A side reaction involving substrate desaturation may also occur.

In subsequent C–C bond cleavage step, MD simulations reveal that the bulky Phe182 residue in WT-CYP199A4 obstructs the formation of a stable hydrogen bond between the hydroxyl group of the α-hydroxyketone intermediate (4-OH-pIBA) and POA. This inhibition leads to the destabilization of the substrate within the active site, thereby preventing efficient C–C bond cleavage. In contrast, in the F182L mutant of CYP199A4, the absence of the bulky phenyl group allows for the formation of a hydrogen bond between 4-OH-pIBA and POA. This interaction stabilizes the substrate in the active site and significantly enhances the efficiency of C–C bond cleavage.

Furthermore, MD simulations demonstrate that when the hydrophobic methyl group of 4-pIBA is replaced with a hydrogen atom, WT-CYP199A4 retains its ability to catalyze the hydroxylation of the modified substrate. However, the increased flexibility of the substrate’s hydrophobic terminus, resulting from the loss of the methyl group, prevents the formation of a stable hydrogen bond with POA. Consequently, the substrate is displaced from the active site, inhibiting C–C bond cleavage. This study provides insights into the catalytic mechanism of CYP199A4 at the molecular level, particularly the role of residue mutations in modulating enzyme activity.

## Figures and Tables

**Table 1:** The Mulliken Spin Densities and Mulliken Charges of the selected moieties at the stationary points along the reaction path of the CYP199A4-catalyzed C–H bond hydroxylation of 4-pIBA.

Spin Densities					
	^2^RE1_r1_(*S*)_	^4^TS1_r1_(*S*)_	^4^INT1_r1_(*S*)_	^4^TS2_r1_(*S*)_	^4^PR1_r1_(*S*)_
Porphrin	−0.25	0.17	−0.14	−0.14	−0.05
Fe	1.29	1.16	1.78	2.12	2.56
oxo	0.81	0.54	0.16	0.02	0.00
4-pIBA	0.00	0.47	1.00	0.83	0.00
C2’	0.00	0.36	0.72	0.56	0.00
O4’	0.00	0.10	0.27	0.23	0.00
H4	0.00	−0.04	0.00	0.00	0.00
Cys358	−0.85	0.71	0.19	0.17	0.49
Mulliken Charges					
	^2^RE1_r1_(*S*)_	^2^TS1_r1_(*S*)_	^2^INT1_r1_(*S*)_	^2^TS2_r1_(*S*)_	^2^PR1_r1_(*S*)_
Porphrin	−0.95	−0.43	−0.39	−0.32	−0.78
Fe	0.61	−0.02	−0.21	−0.19	0.15
oxo	−0.33	−0.51	−0.56	−0.58	−0.45
4-pIBA	−0.67	−0.89	−0.74	−0.67	−0.58
C2’	−0.05	−0.27	−0.12	−0.02	0.12
O4’	−0.21	−0.38	−0.41	−0.40	−0.38
H4	0.07	0.29	0.30	0.32	0.28
Cys358	0.02	0.01	−0.06	−0.08	−0.24

**Table 2:** The Mulliken spin population and Mulliken charges of the stationary points along the reaction path of the CYP199A4-catalyzed C–C bond cleavage of 4-2OH-pIBA.

Spin Densities							
	^2^RE2_r2_	^2^TS1_r2_	^2^INT1_r2_	^2^TS2_r2_	^2^INT2_r2_	^2^TS3_r2_	^2^PR2_r2_
Porphrin	−0.10	−0.11	−0.10	−0.09	−0.09	−0.10	−0.10
Fe	1.08	0.77	0.82	1.03	1.07	0.37	0.96
O_p_	−0.44	0.26	0.23	−0.08	0.03	−0.15	0.10
O_d_	−0.63	0.12	0.09	−0.11	0.00	−0.10	0.00
4-2OH-pIBA	0.01	0.00	0.00	−0.72	−0.98	0.97	0.00
C1’	0.00	0.00	0.00	0.02	0.00	0.00	0.00
C2’	0.00	0.00	0.00	−0.26	−0.85	0.80	0.00
Cys358	0.08	−0.04	−0.04	0.05	−0.02	0.00	0.04
Mulliken Charges							
	^2^RE2_r2_	^2^TS1_r2_	^2^INT1_r2_	^2^TS2_r2_	^2^INT2_r2_	^2^TS3_r2_	^2^PR2_r2_
Porphrin	−0.77	−0.93	−0.88	−0.72	−1.19	−1.06	−0.90
Fe	−0.15	−0.14	−0.14	−0.14	0.53	−0.15	−0.08
O_p_	−0.26	−0.34	−0.36	−0.42	−0.34	−0.47	−0.63
O_d_	−0.26	−0.28	−0.24	−0.17	−0.17	−0.30	−0.52
4-2OH-pIBA	−0.80	−1.14	−0.91	−0.83	−0.64	−0.81	−0.48
C1’	0.22	0.36	0.31	0.37	0.28	0.36	0.31
C2’	0.22	0.21	0.21	0.15	−0.03	−0.04	0.15
Cys358	−0.44	−0.61	−0.60	−0.45	−0.23	−0.59	−0.56

## Data Availability

The data presented in this study are available on request from the corresponding author.

## Supplementary Materials

The following supporting information can be downloaded at: https://www.mdpi.com/article/10.3390/ijms26041526/s1.
