# Supplementary material for: Mechanistic Insights into CYP199A4-Catalyzed α-Hydroxyketone Formation and Hydrogen Bond-Assisted C–C Bond Cleavage Catalyzed by the CYP199A4 F182L Mutant
Source: Int J Mol Sci. 2025 Feb 11;26(4):1526. doi: 10.3390/ijms26041526 (PMC11854964; doi:10.3390/ijms26041526)
Supplement: Supplementary file 1 [file ijms-26-01526-s001.zip › ijms-3455674-supplementary.pdf]

# Supplementary Materials

# Mechanistic Insights into CYP199A4-Catalyzed $\alpha$-Hydroxyketone Formation and Hydrogen Bond-assisted C–C Bond Cleavage Catalyzed by the CYP199A4 F182L Mutant

**Chang Yuan[1,2], Jiaqi Xu[2], Shun Wang[1], Ye-Guang Fang[1,2]* and Hongwei Tan[2]***

[1] Institute of New Materials & Industrial Technologies, Wenzhou University, Wenzhou 325024, China;
[2] Key Laboratories of Theoretical and Computational Photochemistry, Ministry of Education, Beijing Normal University, Beijing, China, 100875

## 1 Schemes

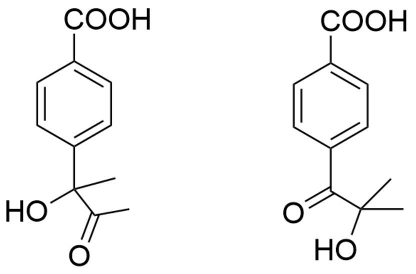


JCM1 JCM2

**Scheme S1.** Structures of the 4-(1'-hydroxy-1'-methyl-2'-oxopropyl)benzoic acid and 4-(1'-oxo-2'-hydroxy-2'-methylpropyl)benzoic acid.

## 2 Figures

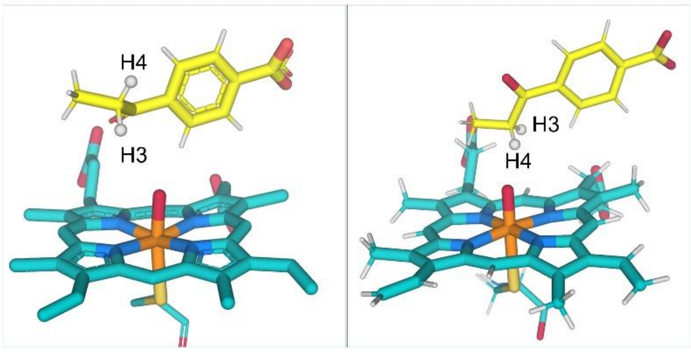


Initial conformation of conf-2 The conformation after MD simulation

**Figure S1**. The initial conformation and the MD simulated conformation of CYP199A4/Cpd I/4-pIBA complex in conf-2.

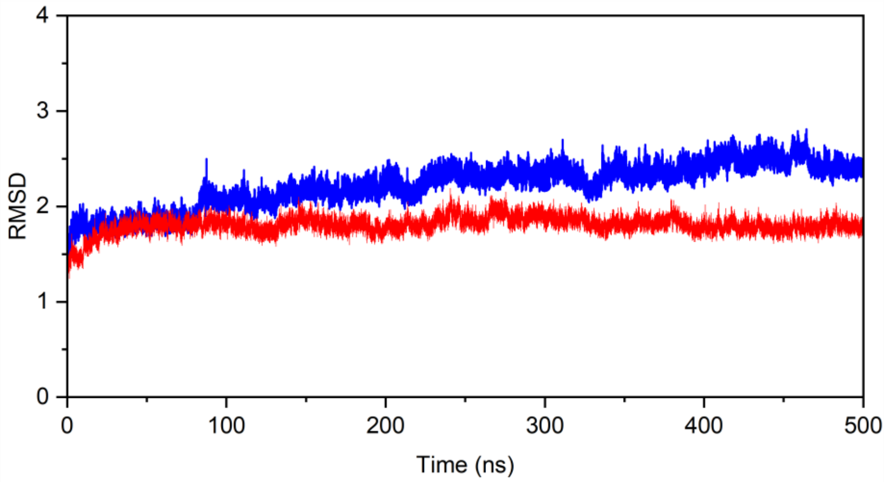


**Figure S2.** The root-mean-square deviation (RMSD) values for the CYP199A4/Cpd I/4-pIBA and CYP199A4/Cpd I/4-2oIBA in conf-1 during 500 ns simulation.

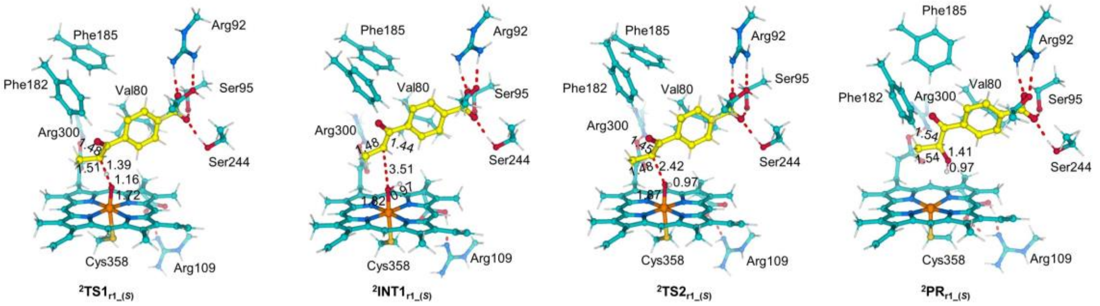


**Figure S3.** The key structural information of the stationary points along the CYP199A4-catalyzed C-H bond hydroxylation (Å).

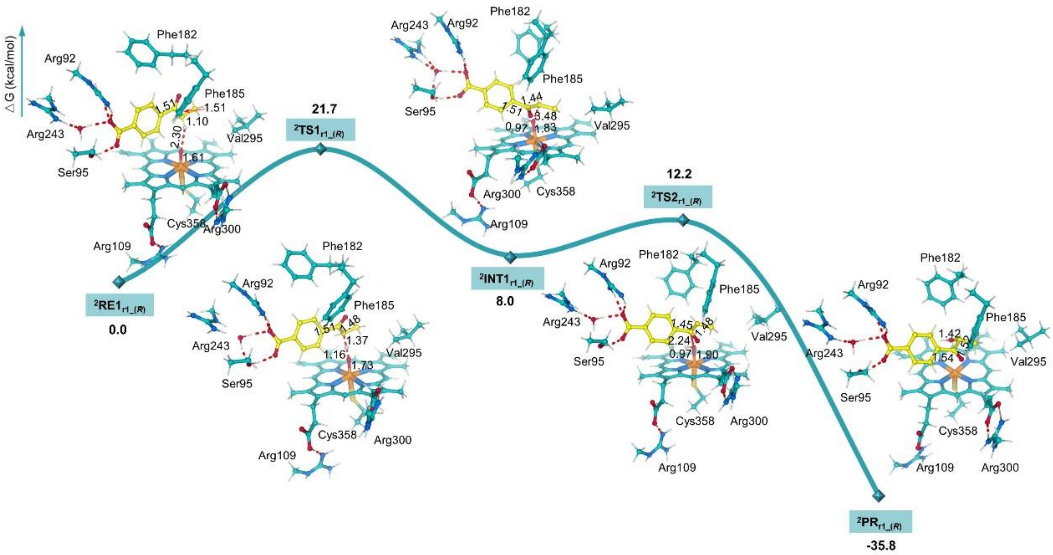


**Figure S4.** Energy profile of CYP199A4-catalysed C-H bond hydroxylation of 4-pIBA to product (*R*)-enantiomer. The key structure information of the stationary points along the reaction pathway in doublet state are shown (Å).

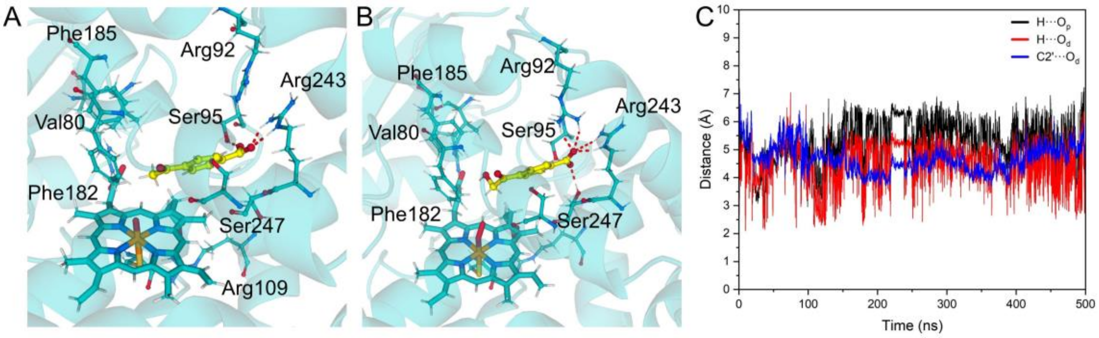


**Figure S5.** MD simulated complexes of WT-CYP199A4/POA/4-pIBA$_{no_Me}$ (A) and WT-CYP199A4/POA/4-2OHpIBA$_{no_Me}$ (B). The distance of the POA to the 4-2OHpIBA$_{no_Me}$ in WT-CYP199A4.

# 3 Tables

**Table S1.** The Mulliken Spin Densities and Mulliken Charges of the selected moieties at the stationary points along the reaction path of the CYP199A4-catalyzed C-H bond hydroxylation of 4-pIBA.

| Spin Densities | | | | | |
|---|---|---|---|---|---|
| | $^4$RE1$_{r1_(S)}$ | $^2$TS1$_{r1_(S)}$ | $^2$INT1$_{r1_(S)}$ | $^2$TS2$_{r1_(S)}$ | $^2$PR1$_{r1_(S)}$ |
| Porphrin | -0.25 | -0.33 | -0.08 | -0.38 | -0.12 |
| Fe | 0.00 | 0.47 | 1.00 | 0.82 | 0.00 |
| oxo | 1.30 | 1.17 | 0.68 | 1.14 | 1.14 |
| 4-pIBA | 0.79 | 0.52 | 0.13 | -0.02 | 0.00 |
| C1’ | 0.00 | 0.39 | 0.79 | 0.62 | 0.00 |
| O4’ | 0.00 | 0.11 | 0.29 | 0.24 | 0.00 |
| H4 | 0.00 | -0.06 | 0.00 | 0.00 | 0.00 |
| Cys358 | -0.85 | -0.77 | -0.73 | -0.56 | -0.02 |
| **Mulliken Charges** | | | | | |
| | $^4$RE1$_{r1_(S)}$ | $^2$TS1$_{r1_(S)}$ | $^2$INT1$_{r1_(S)}$ | $^2$TS2$_{r1_(S)}$ | $^2$PR1$_{r1_(S)}$ |
| Porphrin | -0.58 | -0.88 | -1.01 | -0.94 | -0.73 |
| Fe | 0.11 | 0.55 | 0.44 | 0.53 | -0.03 |
| oxo | -0.45 | -0.36 | -0.35 | -0.38 | -0.45 |
| 4-pIBA | -0.91 | -0.79 | -0.64 | -0.61 | -0.58 |
| C1’ | -0.22 | -0.17 | -0.13 | -0.01 | 0.12 |
| O4’ | -0.38 | -0.24 | -0.23 | -0.24 | -0.38 |
| H4 | 0.17 | 0.20 | 0.17 | 0.18 | 0.28 |
| Cys358 | 0.01 | 0.04 | 0.13 | -0.01 | -0.11 |

## 4 Cartesian coordinates of stationary points

### The α-Hydroxyketone Formation Reaction

**$^{2}$RE1$_{r1_(S)}$**

| | | | |
|---|---|---|---|
| C | 33.067005 | 60.178993 | 0.247003 |
| N | 34.036484 | 59.389133 | 0.981543 |
| C | 34.144592 | 59.191528 | 2.319936 |
| N | 33.214977 | 59.638809 | 3.164014 |
| N | 35.194916 | 58.514503 | 2.786949 |
| H | 32.787994 | 61.113998 | 0.731997 |
| H | 32.121998 | 59.689003 | 0.017000 |
| H | 34.817860 | 59.046436 | 0.437539 |
| H | 32.433189 | 60.182732 | 2.826037 |
| H | 33.274830 | 59.440502 | 4.187315 |
| H | 35.901356 | 58.219646 | 2.123008 |
| H | 35.559208 | 58.686378 | 3.777055 |
| C | 39.457001 | 61.788998 | 2.672004 |
| C | 39.758854 | 60.487015 | 3.419165 |
| O | 40.111649 | 60.682407 | 4.763166 |
| H | 38.532997 | 62.254002 | 3.012996 |
| H | 38.894161 | 59.798008 | 3.319946 |
| H | 40.609112 | 59.985340 | 2.918840 |
| H | 39.322292 | 60.577076 | 5.328230 |
| C | 43.002014 | 58.169003 | 5.366994 |
| C | 42.288795 | 57.164581 | 4.458693 |
| C | 43.197769 | 56.376858 | 3.495020 |
| C | 44.222206 | 55.496777 | 4.223719 |
| C | 42.358368 | 55.539516 | 2.521372 |
| H | 43.663986 | 57.675999 | 6.079012 |
| H | 41.716900 | 56.446560 | 5.076254 |
| H | 41.535053 | 57.708878 | 3.863225 |
| H | 43.760880 | 57.120323 | 2.896678 |
| H | 44.885902 | 56.084625 | 4.876616 |
| H | 43.729912 | 54.735695 | 4.853158 |
| H | 44.868797 | 54.960678 | 3.509410 |
| H | 42.994087 | 55.003738 | 1.796663 |
| H | 41.760143 | 54.784168 | 3.059690 |
| H | 41.656189 | 56.168636 | 1.949492 |
| C | 46.632008 | 61.601997 | 3.442006 |
| N | 47.051792 | 60.726871 | 4.525215 |
| C | 46.172630 | 59.819206 | 5.057980 |
| N | 45.011963 | 59.633812 | 4.518161 |
| N | 46.631680 | 59.188122 | 6.206219 |
| H | 45.687996 | 62.091995 | 3.678995 |
| H | 46.524998 | 61.039005 | 2.513998 |
| H | 47.740387 | 61.097744 | 5.168198 |
| H | 44.819862 | 59.331532 | 2.906823 |
| H | 44.506855 | 58.869576 | 4.967346 |
| H | 47.635384 | 59.035488 | 6.245070 |
| H | 46.115379 | 58.352436 | 6.459452 |
| C | 38.600147 | 45.732487 | 6.324253 |
| C | 37.704304 | 46.866463 | 5.884554 |
| C | 37.982105 | 48.188396 | 6.273755 |
| C | 36.574684 | 46.635857 | 5.085313 |
| C | 37.161327 | 49.249027 | 5.882390 |
| C | 35.746620 | 47.691643 | 4.687941 |
| C | 36.036896 | 48.999352 | 5.085151 |
| H | 38.278011 | 44.771236 | 5.896403 |
| H | 38.602257 | 45.626015 | 7.422525 |
| H | 38.858177 | 48.391987 | 6.897441 |
| H | 36.339233 | 45.614853 | 4.769782 |
| H | 37.397972 | 50.268978 | 6.197294 |
| H | 34.869949 | 47.488174 | 4.066426 |
| H | 35.391178 | 49.826038 | 4.777766 |
| C | 38.004978 | 50.015003 | 0.003029 |
| C | 38.059498 | 51.296516 | 0.800654 |
| C | 38.319164 | 52.526577 | 0.173387 |
| C | 37.899750 | 51.284908 | 2.195512 |
| C | 38.425766 | 53.705082 | 0.916796 |
| C | 38.004639 | 52.461094 | 2.945900 |
| C | 38.270721 | 53.676334 | 2.306751 |
| H | 37.410019 | 49.256996 | 0.505017 |
| H | 39.018002 | 49.660999 | 0.194990 |
| H | 38.444458 | 52.559265 | 0.913690 |
| H | 37.693913 | 50.340778 | 2.708333 |
| H | 38.636562 | 54.650112 | 0.407503 |
| H | 37.885426 | 52.426991 | 4.032280 |
| H | 38.359432 | 54.594242 | 2.893590 |
| C | 35.537994 | 62.243004 | 7.994999 |
| C | 36.995670 | 62.617695 | 8.270268 |
| O | 37.761375 | 61.517742 | 8.695227 |
| H | 35.529007 | 61.434998 | 7.263002 |
| H | 37.426788 | 63.088017 | 7.360973 |
| H | 37.048164 | 63.379345 | 9.069233 |
| H | 37.855946 | 60.919403 | 7.930591 |

| | | | |
|---|---|---|---|
| C | 32.438999 | 58.308994 | 7.789006 |
| C | 33.724079 | 58.372070 | 6.979375 |
| O | 33.658760 | 59.351513 | 5.939711 |
| H | 31.617001 | 57.879005 | 7.216995 |
| H | 34.560379 | 58.630878 | 7.652016 |
| H | 33.953938 | 57.373421 | 6.560654 |
| H | 34.602509 | 59.545963 | 5.718897 |
| C | 49.116001 | 51.857994 | 1.978000 |
| N | 48.653217 | 52.056477 | 3.335541 |
| C | 47.353737 | 52.281013 | 3.714560 |
| N | 46.396343 | 52.061642 | 2.738674 |
| N | 46.985321 | 52.707661 | 4.876504 |
| H | 50.017998 | 52.440002 | 1.785000 |
| H | 48.345001 | 52.209003 | 1.293000 |
| H | 49.345623 | 52.148724 | 4.067410 |
| H | 46.511097 | 51.237354 | 2.158118 |
| H | 45.448189 | 52.181782 | 3.080597 |
| H | 47.748875 | 52.945824 | 5.508494 |
| H | 45.619564 | 52.524952 | 5.670234 |
| C | 44.279991 | 54.723000 | 4.293995 |
| C | 43.205799 | 53.730110 | 4.743934 |
| S | 41.557945 | 54.495316 | 4.920788 |
| H | 43.964008 | 55.214005 | 3.374000 |
| H | 43.431969 | 53.325264 | 5.747798 |
| H | 43.133247 | 52.876911 | 4.054454 |
| C | 44.303967 | 51.916683 | 9.899006 |
| C | 36.562447 | 50.662762 | 5.959836 |
| C | 37.139744 | 58.452126 | 4.589240 |
| C | 43.489956 | 57.245022 | 9.970302 |
| N | 41.366165 | 52.696365 | 2.111682 |
| C | 43.917374 | 52.059666 | 8.421402 |
| C | 36.428459 | 49.587410 | 6.756796 |
| C | 35.935829 | 58.987095 | 4.860042 |
| C | 44.913433 | 57.069202 | 0.542250 |
| N | 39.124744 | 52.320553 | 3.839627 |
| C | 45.116024 | 52.276787 | 7.510658 |
| C | 45.166176 | 57.738625 | 1.883627 |
| N | 40.951408 | 55.513680 | 2.166868 |
| C | 42.690533 | 54.359669 | 0.890867 |
| C | 40.554165 | 50.540932 | 2.949647 |
| C | 37.354820 | 53.481647 | 5.066642 |
| C | 39.480824 | 57.294746 | 2.992626 |
| C | 42.885654 | 49.353325 | 1.306542 |
| C | 38.668549 | 48.653763 | 4.522624 |

| | | | |
|---|---|---|---|
| C | 35.721558 | 55.921848 | 6.046047 |
| C | 41.317219 | 59.174816 | 1.391966 |
| C | 42.381012 | 53.055305 | 1.269130 |
| C | 39.479443 | 51.005238 | 3.705764 |
| C | 37.650410 | 54.785145 | 4.686958 |
| C | 40.566620 | 56.830036 | 2.268614 |
| O | 38.975426 | 53.787468 | 1.559283 |
| O | 46.257996 | 52.363251 | 7.922219 |
| O | 44.690571 | 58.977547 | 1.955189 |
| C | 43.127960 | 51.884842 | 0.829893 |
| C | 38.596676 | 50.147816 | 4.470634 |
| C | 36.889084 | 55.944256 | 5.110128 |
| C | 41.446747 | 57.686287 | 1.490706 |
| O | 44.770779 | 52.372509 | 6.238186 |
| O | 45.757542 | 57.184151 | 2.789125 |
| C | 42.525192 | 50.802826 | 1.420475 |
| C | 37.685944 | 50.986309 | 5.085236 |
| C | 37.482792 | 57.035381 | 4.505730 |
| C | 42.362160 | 56.856701 | 0.888744 |
| C | 41.422272 | 51.328243 | 2.209076 |
| C | 38.035408 | 52.338188 | 4.661993 |
| C | 38.609547 | 56.508625 | 3.742553 |
| C | 42.026245 | 55.504089 | 1.309889 |
| N | 38.688122 | 55.152618 | 3.868122 |
| Fe | 39.927971 | 53.900738 | 2.855897 |
| H | 44.982033 | 52.743553 | 0.161711 |
| H | 44.908112 | 51.004898 | 0.021966 |
| H | 35.739948 | 51.387188 | 5.958398 |
| H | 37.963783 | 59.149307 | 4.401008 |
| H | 43.429218 | 56.660946 | 9.035531 |
| H | 43.356548 | 58.295509 | 9.677153 |
| H | 43.228458 | 52.907703 | 8.268519 |
| H | 43.362968 | 51.176788 | 8.061519 |
| H | 37.207840 | 48.829803 | 6.860958 |
| H | 35.524319 | 49.448460 | 7.355318 |
| H | 35.041893 | 58.379940 | 5.015967 |
| H | 35.807926 | 60.071754 | 4.903264 |
| H | 45.632530 | 57.493713 | 9.819265 |
| H | 45.179546 | 56.011120 | 0.669196 |
| H | 43.529350 | 54.492088 | 0.209698 |
| H | 40.726055 | 49.465061 | 2.934119 |
| H | 36.505737 | 53.344299 | 5.733997 |
| H | 39.291153 | 58.366482 | 2.961884 |
| H | 43.069111 | 48.897617 | 2.294104 |

H 42.081207 48.769539 0.827448
H 43.794987 49.215012 0.704603
H 39.085529 48.232094 3.595975
H 39.299381 48.296345 5.356282
H 37.667446 48.219883 4.666703
H 35.675339 56.851154 6.634020
H 34.761078 55.831711 5.507698
H 35.778065 55.080395 6.752766
H 42.185360 59.617710 0.887845
H 40.408123 59.471794 0.840952
H 41.257214 59.637196 2.390539
C 39.613773 51.645611 8.493936
O 37.869633 52.599567 6.540789
C 39.433067 53.142056 8.267061
O 37.901070 59.940952 6.329438
C 38.441639 53.476681 7.170705
O 36.169193 59.239613 5.106819
C 38.160755 54.922356 6.854611
C 38.868393 55.991615 7.432105
C 38.566711 57.310307 7.085647
C 37.536118 57.593639 6.177719
C 36.820877 56.525238 5.611126
C 37.133938 55.209309 5.937398
C 37.192661 59.037258 5.848535
H 39.975643 51.140392 7.584813
H 38.660885 51.174210 8.777458
H 40.334301 51.470490 9.305623
H 40.395966 53.617393 8.004642
H 39.119652 53.634918 9.205611
H 39.659275 55.802647 8.159652
H 39.119041 58.145046 7.519977
H 36.007687 56.736488 4.915214
H 36.582817 54.374763 5.500523
H 37.543724 50.190578 0.989140
H 33.521866 60.459015 0.713096
H 39.360664 61.604191 1.586938
H 34.957809 63.093018 7.597647
H 35.048546 61.878056 8.912625
H 32.147377 59.313831 8.132480
H 32.591930 57.671734 8.674561
H 49.337410 50.799168 1.745088
H 39.645889 45.904137 6.018384
H 44.448624 55.507877 5.046494
H 45.236534 54.211277 4.102601
H 47.408577 62.364681 3.287513
H 42.270958 58.777050 5.921138
H 43.616249 58.869064 4.774407
H 40.278091 62.507465 2.824288

**$^{4}$TS1$_{r1_(S)}$**

C 33.067001 60.179001 0.247000
N 34.053432 59.427467 0.999372
C 34.327724 59.547600 2.332620
N 33.327137 59.842995 3.176105
N 35.560799 59.387794 2.768430
H 32.787998 61.113998 0.732000
H 32.122002 59.688999 0.017000
H 34.805328 59.026337 0.452878
H 32.377708 59.650696 2.882137
H 33.477287 59.895992 4.199097
H 36.298061 59.325485 2.075258
H 35.843403 59.129593 3.815717
C 39.457001 61.789001 2.672000
C 39.611095 60.395782 3.287880
O 39.830261 60.432827 4.672019
H 38.533001 62.254002 3.013000
H 38.723927 59.778404 3.032156
H 40.477192 59.890541 2.817958
H 39.002941 60.256943 5.167162
C 43.001999 58.168999 5.367000
C 41.637722 57.476883 5.341591
C 41.644821 56.042439 4.781648
C 42.533142 55.091770 5.596619
C 40.216469 55.494934 4.683294
H 43.664001 57.675999 6.079000
H 41.225388 57.451527 6.367902
H 40.929276 58.098175 4.770725
H 42.060768 56.089516 3.755029
H 43.586880 55.410900 5.610126
H 42.185486 55.041233 6.644304
H 42.512646 54.065754 5.195640
H 40.196415 54.490849 4.229812
H 39.750893 55.422165 5.680518
H 39.576866 56.156021 4.075424
C 46.632000 61.602001 13.442000
N 46.994213 60.742352 14.554979
C 47.823833 59.665512 14.468074
N 48.161861 59.156254 13.325173

| | | | |
|---|---|---|---|
| N | 48.219025 | 59.166908 | 15.703984 |
| H | 45.688000 | 62.091999 | 13.679000 |
| H | 46.525002 | 61.039001 | 12.514000 |
| H | 46.583664 | 60.934223 | 15.458380 |
| H | 47.166603 | 58.589615 | 12.088962 |
| H | 48.869915 | 58.429649 | 13.436039 |
| H | 48.406162 | 59.841988 | 16.438274 |
| H | 48.872417 | 58.393501 | 15.684989 |
| C | 33.479000 | 49.292999 | 4.505000 |
| C | 33.189404 | 50.322990 | 5.573093 |
| C | 34.206604 | 50.793404 | 6.419438 |
| C | 31.899343 | 50.855053 | 5.730406 |
| C | 33.953766 | 51.767803 | 7.391218 |
| C | 31.636728 | 51.829212 | 6.698240 |
| C | 32.662647 | 52.288742 | 7.529594 |
| H | 32.601879 | 48.654957 | 4.312473 |
| H | 34.314739 | 48.637600 | 4.796153 |
| H | 35.218391 | 50.387562 | 6.322989 |
| H | 31.088158 | 50.497723 | 5.088129 |
| H | 34.766132 | 52.115322 | 8.035536 |
| H | 30.623280 | 52.226509 | 6.806102 |
| H | 32.458752 | 53.047810 | 8.289431 |
| C | 38.005001 | 50.014999 | -0.003000 |
| C | 37.988419 | 51.309849 | 0.775445 |
| C | 38.784355 | 52.399578 | 0.383711 |
| C | 37.154400 | 51.468121 | 1.892827 |
| C | 38.741024 | 53.608795 | 1.081288 |
| C | 37.106922 | 52.676613 | 2.595047 |
| C | 37.898811 | 53.753551 | 2.189553 |
| H | 37.410000 | 49.257000 | 0.505000 |
| H | 39.018002 | 49.660999 | -0.195000 |
| H | 39.446884 | 52.297710 | -0.481716 |
| H | 36.532093 | 50.630047 | 2.220858 |
| H | 39.373569 | 54.442318 | 0.763549 |
| H | 36.454357 | 52.773579 | 3.466669 |
| H | 37.870384 | 54.694916 | 2.742431 |
| C | 35.537998 | 62.243000 | 7.995000 |
| C | 37.023258 | 62.451077 | 8.326394 |
| O | 37.660667 | 61.246433 | 8.662727 |
| H | 35.528999 | 61.435001 | 7.263000 |
| H | 37.513588 | 62.950043 | 7.463499 |
| H | 37.134899 | 63.134491 | 9.187053 |
| H | 37.701229 | 60.683643 | 7.862299 |
| C | 32.438999 | 58.308998 | 7.789000 |
| C | 33.564301 | 58.787117 | 6.881853 |
| O | 33.165943 | 59.863232 | 6.014642 |
| H | 31.617001 | 57.879002 | 7.217000 |
| H | 34.439655 | 59.096046 | 7.476508 |
| H | 33.910538 | 57.981686 | 6.221263 |
| H | 33.232212 | 60.691792 | 6.508219 |
| C | 49.116001 | 51.858002 | 1.978000 |
| N | 48.661777 | 52.047180 | 3.339925 |
| C | 47.353470 | 51.983952 | 3.756036 |
| N | 46.447243 | 51.573463 | 2.793597 |
| N | 46.928108 | 52.314175 | 4.929648 |
| H | 50.018002 | 52.439999 | 1.785000 |
| H | 48.345001 | 52.209000 | 1.293000 |
| H | 49.347412 | 52.251148 | 4.054743 |
| H | 46.725372 | 50.807034 | 2.190105 |
| H | 45.501762 | 51.483212 | 3.150405 |
| H | 47.635429 | 52.717663 | 5.543583 |
| H | 45.542706 | 52.147949 | 5.769856 |
| C | 44.279999 | 54.723000 | 14.294000 |
| C | 43.061440 | 54.028584 | 14.908786 |
| S | 41.596569 | 55.121922 | 14.924120 |
| H | 43.964001 | 55.214001 | 13.374000 |
| H | 43.237289 | 53.763515 | 15.966512 |
| H | 42.813412 | 53.102329 | 14.373492 |
| C | 44.209763 | 52.297241 | 10.029272 |
| C | 36.582462 | 51.596397 | 16.329605 |
| C | 37.416897 | 59.301418 | 14.633391 |
| C | 43.433048 | 57.626286 | 9.719663 |
| N | 41.330898 | 53.287148 | 12.246946 |
| C | 43.818027 | 52.222961 | 8.546728 |
| C | 36.422199 | 50.572384 | 17.187164 |
| C | 36.244457 | 59.902973 | 14.903371 |
| C | 44.903954 | 57.361717 | 10.072619 |
| N | 39.168446 | 53.073326 | 14.113007 |
| C | 45.020214 | 52.308250 | 7.615054 |
| C | 45.551758 | 58.382267 | 10.996728 |
| N | 41.021515 | 56.112953 | 12.199622 |
| C | 42.621925 | 54.835361 | 10.855879 |
| C | 40.570908 | 51.222923 | 13.330895 |
| C | 37.422619 | 54.343494 | 15.263993 |
| C | 39.690296 | 57.989986 | 13.048267 |
| C | 42.855034 | 49.888767 | 11.731887 |
| C | 38.676373 | 49.470486 | 15.056077 |
| C | 35.883175 | 56.880028 | 16.155191 |

| | | | |
|---|---|---|---|
| C | 41.578701 | 59.730621 | 11.314211 |
| C | 42.313511 | 53.563553 | 11.338180 |
| C | 39.510967 | 51.749287 | 14.069658 |
| C | 37.750214 | 55.617481 | 14.820209 |
| C | 40.725304 | 57.453522 | 12.298871 |
| O | 38.843380 | 54.430981 | 11.779418 |
| O | 46.139175 | 52.595104 | 7.997407 |
| O | 46.712070 | 57.941650 | 11.450386 |
| C | 43.050705 | 52.358479 | 10.983160 |
| C | 38.622158 | 50.957531 | 14.895352 |
| C | 37.044792 | 56.823067 | 15.213680 |
| C | 41.606434 | 58.239223 | 11.449316 |
| O | 44.705296 | 52.054714 | 6.355545 |
| O | 45.078690 | 59.474468 | 11.250233 |
| C | 42.484276 | 51.340103 | 11.710539 |
| C | 37.715336 | 51.846901 | 15.443787 |
| C | 37.688461 | 57.868259 | 14.581355 |
| C | 42.414444 | 57.343342 | 10.792189 |
| C | 41.408291 | 51.936913 | 12.485391 |
| C | 38.082066 | 53.161701 | 14.930205 |
| C | 38.783611 | 57.267235 | 13.822914 |
| C | 42.036896 | 56.022755 | 11.272531 |
| N | 38.791897 | 55.914928 | 13.976862 |
| Fe | 39.995235 | 54.587189 | 13.043589 |
| H | 44.867359 | 53.169350 | 10.166168 |
| H | 44.841103 | 51.427742 | 10.270050 |
| H | 35.774315 | 52.334957 | 16.279499 |
| H | 38.272682 | 59.951630 | 14.420229 |
| H | 43.183830 | 57.018250 | 8.832950 |
| H | 43.336769 | 58.674725 | 9.404094 |
| H | 43.135277 | 53.046444 | 8.273918 |
| H | 43.260265 | 51.300297 | 8.317656 |
| H | 37.184402 | 49.805828 | 17.340588 |
| H | 35.511917 | 50.487255 | 17.786430 |
| H | 35.322727 | 59.345772 | 15.082038 |
| H | 36.172012 | 60.993473 | 14.922214 |
| H | 45.509270 | 57.363857 | 9.148633 |
| H | 45.058598 | 56.366631 | 10.515815 |
| H | 43.416248 | 54.904083 | 10.114692 |
| H | 40.746571 | 50.150166 | 13.407546 |
| H | 36.573914 | 54.261681 | 15.941100 |
| H | 39.561993 | 59.070232 | 13.002896 |
| H | 42.041645 | 49.255287 | 11.338336 |
| H | 43.746254 | 49.695732 | 11.118151 |
| H | 43.072456 | 49.534943 | 12.753650 |
| H | 39.126225 | 48.979340 | 14.180761 |
| H | 39.267483 | 49.169411 | 15.939690 |
| H | 37.665379 | 49.055710 | 15.189129 |
| H | 35.889217 | 57.820911 | 16.726461 |
| H | 34.916603 | 56.830830 | 15.622553 |
| H | 35.898823 | 56.049683 | 16.876804 |
| H | 42.558125 | 60.102745 | 10.983563 |
| H | 40.810020 | 60.063011 | 10.593976 |
| H | 41.352203 | 60.216225 | 12.276151 |
| C | 39.225822 | 51.790722 | 9.749677 |
| O | 36.770805 | 52.826031 | 8.965956 |
| C | 39.018311 | 53.266106 | 9.517662 |
| O | 37.641296 | 59.651741 | 6.259395 |
| C | 37.699409 | 53.630909 | 8.954627 |
| O | 36.179752 | 58.475410 | 5.023849 |
| C | 37.525665 | 54.966152 | 8.283409 |
| C | 38.247265 | 56.121998 | 8.630638 |
| C | 38.060791 | 57.313042 | 7.919477 |
| C | 37.167900 | 57.364719 | 6.838365 |
| C | 36.441826 | 56.208881 | 6.503814 |
| C | 36.603603 | 55.032413 | 7.225090 |
| C | 36.996666 | 58.611565 | 5.987131 |
| H | 39.128448 | 51.232285 | 8.799911 |
| H | 38.458118 | 51.388718 | 10.427451 |
| H | 40.219276 | 51.582085 | 10.165262 |
| H | 39.866821 | 53.807949 | 9.078965 |
| H | 38.988762 | 53.877934 | 10.764233 |
| H | 38.934952 | 56.094608 | 9.478795 |
| H | 38.615120 | 58.213734 | 8.190470 |
| H | 35.761986 | 56.255077 | 5.652633 |
| H | 36.035130 | 54.136532 | 6.969200 |
| H | 37.542995 | 50.162945 | -0.996277 |
| H | 33.508659 | 60.456917 | -0.722493 |
| H | 39.433887 | 61.739155 | 1.568953 |
| H | 35.064789 | 63.145676 | 7.573965 |
| H | 34.976185 | 61.915447 | 8.884834 |
| H | 32.043274 | 59.130600 | 8.409506 |
| H | 32.819771 | 57.530102 | 8.469498 |
| H | 49.344254 | 50.801590 | 1.733708 |
| H | 33.743870 | 49.757767 | 3.578320 |
| H | 44.679756 | 55.499405 | 14.964495 |
| H | 45.080574 | 54.000439 | 14.067360 |

H 47.391029 62.391205 13.279618
H 42.897453 59.223270 5.666784
H 43.489174 58.146805 4.376661
H 40.299862 62.430115 2.973702

**$^{4}$INT1$_{r1_(S)}$**

C 33.067043 60.179104 0.246787
N 34.029991 59.387444 0.985864
C 34.118370 59.187008 2.324461
N 33.186348 59.650543 3.156890
N 35.148731 58.490257 2.807199
H 32.787956 61.113964 0.732045
H 32.122002 59.688953 0.017100
H 34.818218 59.046749 0.450673
H 32.431534 60.228447 2.814080
H 33.235474 59.457203 4.181758
H 35.851551 58.170704 2.150815
H 35.516392 58.705387 3.789250
C 39.457047 61.789104 2.671613
C 39.776863 60.473316 3.384747
O 40.170391 60.631950 4.721417
H 38.533058 62.253868 3.013336
H 38.910984 59.783524 3.291230
H 40.614647 59.984138 2.853915
H 39.373039 60.643482 5.286673
C 43.001396 58.169178 5.367771
C 42.381886 57.182102 4.375898
C 43.371334 56.498966 3.412962
C 44.416332 55.642933 4.140409
C 42.620659 55.664871 2.366418
H 43.664780 57.675907 6.078213
H 41.824799 56.400867 4.926629
H 41.625053 57.719810 3.778536
H 43.912243 57.302307 2.874507
H 45.028522 56.236206 4.837131
H 43.943359 54.835758 4.726116
H 45.112061 55.169720 3.427982
H 43.314014 55.210423 1.638806
H 42.054310 54.846020 2.843099
H 41.898857 56.278423 1.802279
C 46.632153 61.602135 13.442008
N 47.081696 60.737682 14.520269
C 46.269913 59.738758 14.995801
N 45.150932 59.462234 14.411101
N 46.747852 59.119041 16.142439
H 45.687946 62.091915 13.678965
H 46.524899 61.038948 12.514042
H 47.693253 61.160820 15.208090
H 44.966393 59.243305 12.792666
H 44.716335 58.628181 14.806702
H 47.758999 59.048214 16.210102
H 46.290310 58.242931 16.370665
C 33.478706 49.292767 4.504878
C 33.785347 50.165054 5.698039
C 35.039776 50.783363 5.827165
C 32.844669 50.356407 6.721613
C 35.352695 51.558220 6.946347
C 33.149162 51.134998 7.843415
C 34.405025 51.735889 7.960875
H 32.409130 49.035259 4.460615
H 34.038483 48.342136 4.558923
H 35.788536 50.656029 5.039420
H 31.859634 49.886490 6.640467
H 36.342518 52.014603 7.016158
H 32.400562 51.269650 8.629281
H 34.647518 52.340118 8.839019
C 38.004978 50.014771 -0.003573
C 38.051853 51.299435 0.796416
C 38.056835 52.549015 0.156634
C 38.133595 51.274406 2.198965
C 38.147633 53.734180 0.893201
C 38.222443 52.454964 2.943509
C 38.231182 53.691364 2.287712
H 37.409966 49.257153 0.505191
H 39.018047 49.660980 -0.194708
H 37.991772 52.593491 -0.935085
H 38.131020 50.311375 2.719184
H 38.155853 54.695755 0.371377
H 38.285633 52.419373 4.034767
H 38.303421 54.613907 2.869382
C 35.537807 62.243118 7.994992
C 36.956497 62.802879 8.097231
O 37.894855 61.825665 8.468912
H 35.529076 61.434937 7.263069
H 37.226284 63.284489 7.132868
H 36.994549 63.598278 8.863559
H 37.951042 61.192165 7.729795
C 32.438969 58.308956 7.789107

| | | | |
|---|---|---|---|
| C | 33.708553 | 58.412189 | 6.960432 |
| O | 33.600025 | 59.404053 | 5.937480 |
| H | 31.617012 | 57.879036 | 7.216956 |
| H | 34.551594 | 58.680027 | 7.621032 |
| H | 33.951958 | 57.424454 | 6.523247 |
| H | 34.534412 | 59.635303 | 5.712127 |
| C | 49.115910 | 51.858013 | 1.978066 |
| N | 48.647724 | 52.057571 | 3.333530 |
| C | 47.376652 | 52.428204 | 3.691485 |
| N | 46.407310 | 52.283558 | 2.713555 |
| N | 47.046288 | 52.918449 | 4.839601 |
| H | 50.018009 | 52.439987 | 1.785009 |
| H | 48.345036 | 52.209003 | 1.292962 |
| H | 49.339321 | 52.111515 | 4.070440 |
| H | 46.436337 | 51.432491 | 2.161437 |
| H | 45.475708 | 52.515736 | 3.043206 |
| H | 47.825397 | 53.084595 | 5.475624 |
| H | 45.704491 | 52.718761 | 5.632194 |
| C | 44.280048 | 54.723007 | 14.293954 |
| C | 43.364990 | 53.563152 | 14.679600 |
| S | 41.612633 | 54.048389 | 14.949443 |
| H | 43.963875 | 55.213978 | 13.374032 |
| H | 43.679688 | 53.127537 | 15.643115 |
| H | 43.403652 | 52.758476 | 13.933707 |
| C | 44.548985 | 51.933186 | 9.868716 |
| C | 36.818390 | 50.341957 | 15.837440 |
| C | 37.592800 | 58.207256 | 15.147056 |
| C | 43.480873 | 57.216293 | 9.900277 |
| N | 41.666965 | 52.592247 | 12.183419 |
| C | 44.111088 | 52.041748 | 8.403164 |
| C | 36.667084 | 49.242962 | 16.598160 |
| C | 36.413616 | 58.753754 | 15.493373 |
| C | 44.949486 | 57.061085 | 10.357949 |
| N | 39.435951 | 52.111752 | 13.888578 |
| C | 45.271042 | 52.315575 | 7.459537 |
| C | 45.256535 | 57.676914 | 11.712835 |
| N | 41.125675 | 55.395504 | 12.213848 |
| C | 42.821369 | 54.296696 | 10.840493 |
| C | 40.960205 | 50.399651 | 13.033493 |
| C | 37.639393 | 53.205021 | 15.140456 |
| C | 39.816029 | 57.136494 | 13.340160 |
| C | 43.332268 | 49.325264 | 11.379196 |
| C | 39.056461 | 48.423573 | 14.473982 |
| C | 36.088345 | 55.594662 | 16.352421 |
| C | 41.499439 | 59.087769 | 11.651156 |
| C | 42.616074 | 52.992878 | 11.279980 |
| C | 39.841053 | 50.813358 | 13.749695 |
| C | 37.977592 | 54.530472 | 14.886357 |
| C | 40.794895 | 56.703285 | 12.457069 |
| O | 39.159847 | 53.629406 | 11.690665 |
| O | 46.432369 | 52.335663 | 7.823821 |
| O | 44.832130 | 58.931915 | 11.826412 |
| C | 43.408878 | 51.855610 | 10.839812 |
| C | 38.944580 | 49.915688 | 14.453324 |
| C | 37.257401 | 55.668041 | 15.420890 |
| C | 41.608494 | 57.594193 | 11.643508 |
| O | 44.871372 | 52.533772 | 6.219572 |
| O | 45.828999 | 57.070648 | 12.596261 |
| C | 42.895313 | 50.754456 | 11.480313 |
| C | 37.980679 | 50.714424 | 15.036183 |
| C | 37.887234 | 56.794731 | 14.923681 |
| C | 42.438766 | 56.790028 | 10.899932 |
| C | 41.792847 | 51.230450 | 12.297360 |
| C | 38.319637 | 52.088425 | 14.673495 |
| C | 38.983681 | 56.312504 | 14.093223 |
| C | 42.132320 | 55.418686 | 11.288089 |
| N | 39.021622 | 54.946869 | 14.101892 |
| Fe | 40.315250 | 53.752689 | 13.092921 |
| H | 45.192329 | 52.795204 | 10.105122 |
| H | 45.201794 | 51.054543 | 9.979415 |
| H | 35.979263 | 51.046452 | 15.804915 |
| H | 38.438370 | 58.890709 | 15.010724 |
| H | 43.357967 | 56.647594 | 8.962387 |
| H | 43.303783 | 58.269104 | 9.639361 |
| H | 43.368832 | 52.843475 | 8.258650 |
| H | 43.605606 | 51.120342 | 8.066074 |
| H | 37.459084 | 48.502743 | 16.728636 |
| H | 35.734001 | 49.065781 | 17.139454 |
| H | 35.501720 | 58.165199 | 15.610823 |
| H | 36.325550 | 59.832722 | 15.644477 |
| H | 45.599987 | 57.543495 | 9.607632 |
| H | 45.250565 | 56.006748 | 10.419672 |
| H | 43.614788 | 54.456795 | 10.113024 |
| H | 41.182606 | 49.333462 | 13.019360 |
| H | 36.778244 | 53.030617 | 15.782673 |
| H | 39.661774 | 58.211540 | 13.415786 |
| H | 43.546543 | 48.892651 | 12.370584 |

| | | | |
|---|---|---|---|
| H | 42.556606 | 48.693604 | 10.913258 |
| H | 44.243443 | 49.228916 | 10.771865 |
| H | 39.537586 | 48.037918 | 13.562835 |
| H | 39.649944 | 48.063824 | 15.333593 |
| H | 38.061947 | 47.958378 | 14.550959 |
| H | 36.088123 | 56.450073 | 17.045231 |
| H | 35.125614 | 55.617062 | 15.811076 |
| H | 36.101402 | 54.675072 | 16.955746 |
| H | 42.308681 | 59.549641 | 11.072241 |
| H | 40.535641 | 59.428093 | 11.235421 |
| H | 41.573750 | 59.487305 | 12.675485 |
| C | 39.948231 | 52.208347 | 8.764075 |
| O | 38.425613 | 52.869980 | 6.415119 |
| C | 39.693935 | 53.569229 | 8.246888 |
| O | 37.848717 | 60.207279 | 6.157846 |
| C | 38.886501 | 53.817272 | 7.078505 |
| O | 36.088760 | 59.354473 | 5.084081 |
| C | 38.530418 | 55.229897 | 6.705003 |
| C | 39.273380 | 56.356392 | 7.101991 |
| C | 38.858223 | 57.641979 | 6.752027 |
| C | 37.678493 | 57.835808 | 6.018554 |
| C | 36.946251 | 56.710152 | 5.606953 |
| C | 37.371712 | 55.425598 | 5.932844 |
| C | 37.186436 | 59.242542 | 5.731652 |
| H | 39.467918 | 51.445423 | 8.138017 |
| H | 39.583199 | 52.156345 | 9.807349 |
| H | 41.033470 | 52.008060 | 8.826040 |
| H | 40.085674 | 54.412731 | 8.820670 |
| H | 39.091564 | 54.500813 | 11.269947 |
| H | 40.190331 | 56.236870 | 7.682765 |
| H | 39.434521 | 58.518635 | 7.050758 |
| H | 36.022873 | 56.850254 | 5.043097 |
| H | 36.800079 | 54.549923 | 5.620614 |
| H | 37.541897 | 50.186115 | -0.988787 |
| H | 33.524818 | 60.457584 | -0.712240 |
| H | 39.359024 | 61.624485 | 1.583138 |
| H | 34.812084 | 63.012188 | 7.679536 |
| H | 35.213646 | 61.831169 | 8.964737 |
| H | 32.135468 | 59.300770 | 8.159031 |
| H | 32.614361 | 57.653606 | 8.657182 |
| H | 49.334858 | 50.798473 | 1.750410 |
| H | 33.744141 | 49.757980 | 3.578506 |
| H | 44.300129 | 55.490181 | 15.084828 |
| H | 45.313377 | 54.369953 | 14.141743 |
| H | 47.398884 | 62.372833 | 13.275457 |
| H | 42.217545 | 58.703999 | 5.924876 |
| H | 43.593674 | 58.937210 | 4.841095 |
| H | 40.277538 | 62.506935 | 2.831499 |

**$^4$TS2$_{r1_(S)}$**

| | | | |
|---|---|---|---|
| C | 33.067001 | 60.179001 | 0.247000 |
| N | 34.052582 | 59.429092 | 1.000936 |
| C | 34.328857 | 59.563404 | 2.332742 |
| N | 33.324203 | 59.858059 | 3.173006 |
| N | 35.562229 | 59.418640 | 2.770164 |
| H | 32.787998 | 61.113998 | 0.732000 |
| H | 32.122002 | 59.688999 | 0.017000 |
| H | 34.807381 | 59.030586 | 0.456532 |
| H | 32.377304 | 59.649227 | 2.882159 |
| H | 33.471451 | 59.926334 | 4.194421 |
| H | 36.298725 | 59.357044 | 2.075858 |
| H | 35.838886 | 59.143631 | 3.824138 |
| C | 39.457001 | 61.789001 | 2.672000 |
| C | 39.604450 | 60.384808 | 3.262146 |
| O | 39.819569 | 60.392956 | 4.646702 |
| H | 38.533001 | 62.254002 | 3.013000 |
| H | 38.717266 | 59.773754 | 2.990057 |
| H | 40.471764 | 59.886726 | 2.787531 |
| H | 38.981487 | 60.252373 | 5.135299 |
| C | 43.001999 | 58.168999 | 5.367000 |
| C | 41.626221 | 57.498829 | 5.382283 |
| C | 41.600883 | 56.049007 | 4.862401 |
| C | 42.491001 | 55.106323 | 5.685487 |
| C | 40.162312 | 55.521793 | 4.807611 |
| H | 43.664001 | 57.675999 | 6.079000 |
| H | 41.233517 | 57.507156 | 6.416593 |
| H | 40.916538 | 58.115185 | 4.807896 |
| H | 41.996555 | 56.060921 | 3.826847 |
| H | 43.548428 | 55.413162 | 5.673702 |
| H | 42.161011 | 55.084255 | 6.739878 |
| H | 42.452236 | 54.071793 | 5.307516 |
| H | 40.117542 | 54.505291 | 4.384235 |
| H | 39.715652 | 55.486343 | 5.815563 |
| H | 39.520672 | 56.175323 | 4.193727 |
| C | 46.632000 | 61.602001 | 13.442000 |
| N | 46.990486 | 60.747604 | 14.561085 |
| C | 47.889217 | 59.725784 | 14.493286 |
| N | 48.323524 | 59.278313 | 13.356622 |

| | | | |
|---|---|---|---|
| N | 48.244228 | 59.214657 | 15.735246 |
| H | 45.688000 | 62.091999 | 13.679000 |
| H | 46.525002 | 61.039001 | 12.514000 |
| H | 46.484333 | 60.857914 | 15.428944 |
| H | 47.390259 | 58.616695 | 12.104234 |
| H | 49.076332 | 58.601444 | 13.487933 |
| H | 48.342312 | 59.874584 | 16.499741 |
| H | 48.938297 | 58.477455 | 15.736091 |
| C | 30.795221 | 49.130581 | 8.113594 |
| C | 31.678749 | 50.301846 | 8.474395 |
| C | 33.077473 | 50.159969 | 8.504943 |
| C | 31.134598 | 51.553688 | 8.797713 |
| C | 33.903870 | 51.231659 | 8.848407 |
| C | 31.959044 | 52.631348 | 9.142675 |
| C | 33.346607 | 52.475071 | 9.169494 |
| H | 30.850124 | 48.335510 | 8.877354 |
| H | 31.099627 | 48.674950 | 7.156514 |
| H | 33.522877 | 49.191402 | 8.255140 |
| H | 30.048372 | 51.686886 | 8.779438 |
| H | 34.990280 | 51.121914 | 8.874152 |
| H | 31.510338 | 53.597469 | 9.391426 |
| H | 34.014187 | 53.298038 | 9.432830 |
| C | 38.005001 | 50.014999 | -0.003000 |
| C | 38.000191 | 51.314167 | 0.768166 |
| C | 38.826038 | 52.383007 | 0.380286 |
| C | 37.153896 | 51.499065 | 1.871932 |
| C | 38.801182 | 53.597923 | 1.068454 |
| C | 37.125278 | 52.713753 | 2.564668 |
| C | 37.947388 | 53.769588 | 2.163816 |
| H | 37.410000 | 49.257000 | 0.505000 |
| H | 39.018002 | 49.660999 | -0.195000 |
| H | 39.498402 | 52.259384 | -0.474632 |
| H | 36.507915 | 50.677811 | 2.196465 |
| H | 39.456940 | 54.414440 | 0.753747 |
| H | 36.463337 | 52.833103 | 3.426364 |
| H | 37.933563 | 54.715126 | 2.710110 |
| C | 35.537998 | 62.243000 | 7.995000 |
| C | 37.025089 | 62.503849 | 8.272834 |
| O | 37.714558 | 61.323006 | 8.588623 |
| H | 35.528999 | 61.435001 | 7.263000 |
| H | 37.467003 | 63.017059 | 7.391985 |
| H | 37.144203 | 63.193417 | 9.127574 |
| H | 37.727062 | 60.751133 | 7.794069 |
| C | 32.438999 | 58.308998 | 7.789000 |
| C | 33.540127 | 58.827789 | 6.875487 |
| O | 33.104248 | 59.894669 | 6.015939 |
| H | 31.617001 | 57.879002 | 7.217000 |
| H | 34.411865 | 59.157341 | 7.464408 |
| H | 33.902767 | 58.037552 | 6.206433 |
| H | 33.128593 | 60.720356 | 6.518031 |
| C | 49.116001 | 51.858002 | 1.978000 |
| N | 48.656296 | 52.044369 | 3.336732 |
| C | 47.344833 | 51.974644 | 3.737466 |
| N | 46.452145 | 51.550426 | 2.768163 |
| N | 46.903843 | 52.310501 | 4.902803 |
| H | 50.018002 | 52.439999 | 1.785000 |
| H | 48.345001 | 52.209000 | 1.293000 |
| H | 49.333652 | 52.260742 | 4.055733 |
| H | 46.741947 | 50.781128 | 2.173883 |
| H | 45.503212 | 51.457184 | 3.114757 |
| H | 47.597954 | 52.718998 | 5.528159 |
| H | 45.554180 | 52.067711 | 5.731780 |
| C | 44.279999 | 54.723000 | 14.294000 |
| C | 43.125038 | 53.942387 | 14.920085 |
| S | 41.552811 | 54.891071 | 14.970330 |
| H | 43.964001 | 55.214001 | 13.374000 |
| H | 43.347351 | 53.686165 | 15.969650 |
| H | 42.950623 | 52.995773 | 14.392836 |
| C | 44.396099 | 52.080193 | 10.039402 |
| C | 36.625557 | 51.666058 | 16.172190 |
| C | 37.763653 | 59.325798 | 14.423831 |
| C | 43.799168 | 57.411137 | 9.621078 |
| N | 41.598080 | 53.195190 | 12.282892 |
| C | 43.944225 | 52.035297 | 8.573337 |
| C | 36.430878 | 50.671757 | 17.056709 |
| C | 36.603390 | 59.961773 | 14.664373 |
| C | 45.247349 | 57.193417 | 10.086842 |
| N | 39.281887 | 53.039440 | 13.973930 |
| C | 45.100163 | 52.175209 | 7.593584 |
| C | 45.830624 | 58.291874 | 10.964186 |
| N | 41.251839 | 56.009380 | 12.019687 |
| C | 42.907116 | 54.670750 | 10.819638 |
| C | 40.706142 | 51.153301 | 13.321497 |
| C | 37.572563 | 54.376553 | 15.102531 |
| C | 39.988247 | 57.931557 | 12.859412 |
| C | 42.965019 | 49.734993 | 11.773154 |
| C | 38.718616 | 49.474869 | 15.007551 |
| C | 36.135647 | 56.973831 | 15.955646 |

| | | | |
|---|---|---|---|
| C | 41.907677 | 59.592991 | 11.084925 |
| C | 42.581867 | 53.430019 | 11.360615 |
| C | 39.618153 | 51.710770 | 13.988422 |
| C | 37.977917 | 55.635281 | 14.672112 |
| C | 40.990749 | 57.351265 | 12.097546 |
| O | 39.166443 | 54.276096 | 11.532340 |
| O | 46.221828 | 52.505802 | 7.932791 |
| O | 46.983959 | 57.916962 | 11.489905 |
| C | 43.267845 | 52.193489 | 11.021576 |
| C | 38.690155 | 50.957706 | 14.807039 |
| C | 37.310707 | 56.866528 | 15.035432 |
| C | 41.907234 | 58.104431 | 11.250367 |
| O | 44.740849 | 51.924713 | 6.347112 |
| O | 45.318783 | 59.383472 | 11.128877 |
| C | 42.659218 | 51.201427 | 11.752637 |
| C | 37.785141 | 51.874355 | 15.309988 |
| C | 37.997280 | 57.884708 | 14.397584 |
| C | 42.719288 | 57.179066 | 10.644593 |
| C | 41.604282 | 51.841553 | 12.517341 |
| C | 38.187099 | 53.172287 | 14.781066 |
| C | 39.079769 | 57.246666 | 13.661718 |
| C | 42.298672 | 55.875504 | 11.149160 |
| N | 39.046616 | 55.892281 | 13.846372 |
| Fe | 40.306458 | 54.542271 | 13.043452 |
| H | 45.095879 | 52.921753 | 10.154659 |
| H | 44.993176 | 51.180840 | 10.257453 |
| H | 35.829201 | 52.413052 | 16.076828 |
| H | 38.640568 | 59.948956 | 14.217262 |
| H | 43.617783 | 56.743145 | 8.762009 |
| H | 43.710121 | 58.435795 | 9.232928 |
| H | 43.237736 | 52.854820 | 8.354090 |
| H | 43.392525 | 51.109783 | 8.341901 |
| H | 37.180637 | 49.903088 | 17.254726 |
| H | 35.504429 | 50.615540 | 17.634184 |
| H | 35.663799 | 59.432713 | 14.835247 |
| H | 36.560207 | 61.053959 | 14.665715 |
| H | 45.912022 | 57.123177 | 9.207530 |
| H | 45.374718 | 56.239498 | 10.620925 |
| H | 43.724731 | 54.700836 | 10.101774 |
| H | 40.852867 | 50.078194 | 13.418074 |
| H | 36.709141 | 54.334503 | 15.763737 |
| H | 39.889389 | 59.013901 | 12.800834 |
| H | 42.124630 | 49.137062 | 11.381005 |
| H | 43.846775 | 49.503197 | 11.159257 |
| H | 43.167595 | 49.373119 | 12.795151 |
| H | 39.168949 | 48.954620 | 14.149228 |
| H | 39.298191 | 49.188766 | 15.903500 |
| H | 37.700127 | 49.079823 | 15.142384 |
| H | 36.164555 | 57.921349 | 16.514761 |
| H | 35.176952 | 56.949627 | 15.407472 |
| H | 36.113220 | 56.153137 | 16.687616 |
| H | 42.888088 | 59.940147 | 10.732688 |
| H | 41.132595 | 59.925613 | 10.372106 |
| H | 41.707756 | 60.102562 | 12.040519 |
| C | 39.186649 | 51.944328 | 9.814982 |
| O | 36.619450 | 53.075928 | 9.359253 |
| C | 38.941963 | 53.369572 | 9.476021 |
| O | 37.600689 | 59.699833 | 6.203680 |
| C | 37.599834 | 53.810097 | 9.151851 |
| O | 36.131405 | 58.482880 | 5.018531 |
| C | 37.427517 | 55.116951 | 8.437182 |
| C | 38.213226 | 56.255928 | 8.688582 |
| C | 38.035221 | 57.417580 | 7.930050 |
| C | 37.094448 | 57.451019 | 6.889761 |
| C | 36.304493 | 56.312759 | 6.652134 |
| C | 36.449078 | 55.170612 | 7.429868 |
| C | 36.945297 | 58.655285 | 5.980233 |
| H | 39.306679 | 51.357719 | 8.882167 |
| H | 38.327652 | 51.517105 | 10.351524 |
| H | 40.103016 | 51.819363 | 10.403518 |
| H | 39.783813 | 53.989902 | 9.168730 |
| H | 38.354374 | 53.862453 | 11.868438 |
| H | 38.945766 | 56.233418 | 9.498580 |
| H | 38.636715 | 58.307045 | 8.127266 |
| H | 35.591259 | 56.340843 | 5.828002 |
| H | 35.831409 | 54.287609 | 7.252627 |
| H | 37.542480 | 50.161766 | -0.996294 |
| H | 33.508415 | 60.456657 | -0.722786 |
| H | 39.441216 | 61.759365 | 1.568015 |
| H | 35.018059 | 63.128426 | 7.592511 |
| H | 35.020546 | 61.896248 | 8.904244 |
| H | 32.036549 | 59.108292 | 8.434049 |
| H | 32.844345 | 57.521812 | 8.445118 |
| H | 49.345612 | 50.802067 | 1.732581 |
| H | 29.740366 | 49.431007 | 8.023115 |
| H | 44.631371 | 55.516506 | 14.973620 |
| H | 45.134491 | 54.060863 | 14.073431 |

| | | | |
|---|---|---|---|
| H | 47.392071 | 62.389240 | 13.279893 |
| H | 42.921703 | 59.230587 | 5.647442 |
| H | 43.471657 | 58.116798 | 4.369347 |
| H | 40.300465 | 62.421337 | 2.990211 |

**[4]PR1$_{r1_(S)}$**

| | | | |
|---|---|---|---|
| C | 33.067051 | 60.178959 | 0.247119 |
| N | 34.029743 | 59.388313 | 0.987841 |
| C | 34.120098 | 59.193790 | 2.327232 |
| N | 33.183990 | 59.651192 | 3.158255 |
| N | 35.157593 | 58.508587 | 2.811577 |
| H | 32.787975 | 61.114021 | 0.731942 |
| H | 32.121986 | 59.689022 | 0.017004 |
| H | 34.818222 | 59.047260 | 0.453240 |
| H | 32.417744 | 60.211540 | 2.811664 |
| H | 33.234589 | 59.461746 | 4.184092 |
| H | 35.868374 | 58.203255 | 2.157007 |
| H | 35.518215 | 58.720936 | 3.795779 |
| C | 39.456978 | 61.788952 | 2.671906 |
| C | 39.794708 | 60.493923 | 3.414380 |
| O | 40.177971 | 60.688057 | 4.749173 |
| H | 38.533047 | 62.254032 | 3.013086 |
| H | 38.940903 | 59.788048 | 3.331692 |
| H | 40.642811 | 60.007332 | 2.897473 |
| H | 39.377556 | 60.687820 | 5.309834 |
| C | 43.001995 | 58.169048 | 5.367030 |
| C | 42.324726 | 57.162430 | 4.434202 |
| C | 43.283291 | 56.260014 | 3.631777 |
| C | 44.056290 | 55.284431 | 4.530548 |
| C | 42.527409 | 55.499866 | 2.534072 |
| H | 43.663979 | 57.675961 | 6.078994 |
| H | 41.637051 | 56.519184 | 5.014538 |
| H | 41.682484 | 57.717533 | 3.728174 |
| H | 44.021740 | 56.920013 | 3.135086 |
| H | 44.643867 | 55.805706 | 5.302356 |
| H | 43.364029 | 54.598160 | 5.048970 |
| H | 44.757339 | 54.660858 | 3.951879 |
| H | 43.205936 | 54.866199 | 1.939115 |
| H | 41.754696 | 54.841240 | 2.966708 |
| H | 42.020390 | 56.192074 | 1.841686 |
| C | 46.631817 | 61.601780 | 13.441922 |
| N | 47.049122 | 60.732124 | 14.520225 |
| C | 47.191944 | 59.369316 | 14.432858 |
| N | 46.995792 | 58.699123 | 13.352160 |
| N | 47.546368 | 58.797260 | 15.655411 |
| H | 45.688087 | 62.092121 | 13.679093 |
| H | 46.525070 | 61.039074 | 12.513947 |
| H | 46.929600 | 61.092472 | 15.458412 |
| H | 46.390751 | 58.202805 | 11.831711 |
| H | 47.243214 | 57.711105 | 13.465586 |
| H | 48.214539 | 59.320232 | 16.213902 |
| H | 47.760735 | 57.807053 | 15.614624 |
| C | 33.479160 | 49.293110 | 4.505085 |
| C | 33.884434 | 50.126766 | 5.696993 |
| C | 35.157516 | 50.717316 | 5.757316 |
| C | 33.017338 | 50.311310 | 6.784890 |
| C | 35.558254 | 51.460022 | 6.870677 |
| C | 33.410378 | 51.056961 | 7.901328 |
| C | 34.682938 | 51.631924 | 7.949074 |
| H | 32.394188 | 49.106277 | 4.495662 |
| H | 33.977283 | 48.307415 | 4.526136 |
| H | 35.848644 | 50.595757 | 4.917637 |
| H | 32.019131 | 49.863541 | 6.757758 |
| H | 36.557312 | 51.900921 | 6.888705 |
| H | 32.717419 | 51.187855 | 8.737204 |
| H | 34.994076 | 52.213184 | 8.820986 |
| C | 38.005112 | 50.015060 | -0.002850 |
| C | 38.047756 | 51.308704 | 0.780189 |
| C | 38.017727 | 52.549412 | 0.123866 |
| C | 38.160927 | 51.303486 | 2.180280 |
| C | 38.105614 | 53.745991 | 0.841797 |
| C | 38.246868 | 52.495510 | 2.905983 |
| C | 38.221085 | 53.723194 | 2.234422 |
| H | 37.409954 | 49.257011 | 0.504962 |
| H | 39.017963 | 49.660946 | -0.195085 |
| H | 37.927887 | 52.577419 | -0.966570 |
| H | 38.186890 | 50.347748 | 2.713085 |
| H | 38.086632 | 54.700218 | 0.307000 |
| H | 38.336517 | 52.474419 | 3.994912 |
| H | 38.291878 | 54.654564 | 2.802137 |
| C | 35.538074 | 62.242939 | 7.995004 |
| C | 36.934341 | 62.861977 | 8.045098 |
| O | 37.922913 | 61.934608 | 8.411865 |
| H | 35.528919 | 61.435028 | 7.262971 |
| H | 37.156864 | 63.333782 | 7.063678 |
| H | 36.959007 | 63.675117 | 8.793209 |
| H | 37.970737 | 61.274326 | 7.696248 |
| C | 32.438999 | 58.309044 | 7.788904 |

| | | | |
|---|---|---|---|
| C | 33.707569 | 58.414818 | 6.958681 |
| O | 33.596924 | 59.409657 | 5.938548 |
| H | 31.616982 | 57.878952 | 7.217064 |
| H | 34.551273 | 58.681435 | 7.618741 |
| H | 33.951534 | 57.428665 | 6.518092 |
| H | 34.531242 | 59.644493 | 5.716718 |
| C | 49.116016 | 51.858013 | 1.978032 |
| N | 48.740948 | 52.067230 | 3.367513 |
| C | 47.518883 | 51.732903 | 3.900094 |
| N | 46.818932 | 50.770355 | 3.207195 |
| N | 46.999416 | 52.264755 | 4.960173 |
| H | 50.017998 | 52.439991 | 1.784961 |
| H | 48.345001 | 52.209000 | 1.293000 |
| H | 49.234959 | 52.795837 | 3.866168 |
| H | 47.350433 | 50.033787 | 2.757543 |
| H | 45.974174 | 50.453545 | 3.671726 |
| H | 47.529133 | 53.047569 | 5.339862 |
| H | 45.923176 | 51.547485 | 5.880832 |
| C | 44.279930 | 54.723000 | 14.293984 |
| C | 43.535583 | 53.410240 | 14.515909 |
| S | 41.721897 | 53.616306 | 14.741006 |
| H | 43.964027 | 55.214008 | 13.373995 |
| H | 43.899742 | 52.916893 | 15.432764 |
| H | 43.720409 | 52.710258 | 13.686307 |
| C | 44.597996 | 50.324581 | 10.002618 |
| C | 36.158180 | 51.137066 | 15.090467 |
| C | 38.349716 | 58.496201 | 13.122173 |
| C | 44.771778 | 55.658875 | 9.419980 |
| N | 41.724693 | 51.875938 | 11.870058 |
| C | 44.311001 | 50.340729 | 8.481960 |
| C | 35.719925 | 50.221981 | 15.973398 |
| C | 37.238323 | 59.253025 | 13.123889 |
| C | 46.088909 | 55.394798 | 10.175140 |
| N | 39.263870 | 52.088558 | 13.280944 |
| C | 45.238888 | 51.249210 | 7.682168 |
| C | 46.438042 | 56.354309 | 11.298299 |
| N | 41.756485 | 54.674713 | 11.470997 |
| C | 43.406555 | 53.104591 | 10.560376 |
| C | 40.495266 | 50.006580 | 12.881366 |
| C | 37.551144 | 53.654205 | 14.058860 |
| C | 40.621090 | 56.782772 | 12.016852 |
| C | 42.701988 | 48.250641 | 11.648088 |
| C | 38.120697 | 48.665428 | 14.341555 |
| C | 36.270447 | 56.428120 | 14.511941 |
| C | 42.906651 | 58.109192 | 10.454996 |
| C | 42.850483 | 51.941319 | 11.084266 |
| C | 39.421005 | 50.728004 | 13.391122 |
| C | 38.138767 | 54.831577 | 13.613849 |
| C | 41.646893 | 56.049728 | 11.441424 |
| O | 39.640762 | 54.314533 | 9.218626 |
| O | 45.842281 | 52.188580 | 8.172783 |
| O | 46.128304 | 57.621723 | 11.046039 |
| C | 43.402126 | 50.616135 | 10.862351 |
| C | 38.302589 | 50.128899 | 14.087131 |
| C | 37.554722 | 56.143291 | 13.799071 |
| C | 42.735989 | 56.640179 | 10.690022 |
| O | 45.276611 | 50.929035 | 6.404558 |
| O | 46.991093 | 55.980824 | 12.317928 |
| C | 42.592953 | 49.740978 | 11.544545 |
| C | 37.450325 | 51.167953 | 14.411310 |
| C | 38.432125 | 57.041588 | 13.222127 |
| C | 43.520794 | 55.596287 | 10.254609 |
| C | 41.549126 | 50.541283 | 12.156304 |
| C | 38.078136 | 52.379276 | 13.899914 |
| C | 39.538265 | 56.247570 | 12.704815 |
| C | 42.903645 | 54.383430 | 10.760625 |
| N | 39.338703 | 54.914631 | 12.947933 |
| Fe | 40.672920 | 53.414913 | 12.646551 |
| H | 45.386528 | 51.065632 | 10.203717 |
| H | 45.017006 | 49.346706 | 10.284177 |
| H | 35.477417 | 51.960068 | 14.844687 |
| H | 39.307957 | 59.018505 | 13.026847 |
| H | 44.713917 | 54.916405 | 8.607194 |
| H | 44.830559 | 56.646358 | 8.939919 |
| H | 43.289612 | 50.709297 | 8.282382 |
| H | 44.346581 | 49.333393 | 8.040776 |
| H | 36.338013 | 49.389622 | 16.315809 |
| H | 34.718388 | 50.300838 | 16.404341 |
| H | 36.234055 | 58.826763 | 13.167526 |
| H | 37.308147 | 60.340866 | 13.045809 |
| H | 46.919899 | 55.444309 | 9.448781 |
| H | 46.116535 | 54.383053 | 10.600451 |
| H | 44.303165 | 53.001228 | 9.949138 |
| H | 40.496002 | 48.928692 | 13.038243 |
| H | 36.602654 | 53.741104 | 14.585412 |
| H | 40.654331 | 57.863384 | 11.889922 |
| H | 42.756107 | 47.917831 | 12.698164 |
| H | 41.832294 | 47.745525 | 11.194343 |

| | | | |
|---|---|---|---|
| H | 43.601288 | 47.878487 | 11.137003 |
| H | 38.620068 | 48.051788 | 13.577268 |
| H | 38.530556 | 48.362896 | 15.321724 |
| H | 37.052895 | 48.398312 | 14.341223 |
| H | 36.309738 | 57.410892 | 15.005556 |
| H | 35.409027 | 56.446774 | 13.820556 |
| H | 36.053711 | 55.673969 | 15.282904 |
| H | 43.817982 | 58.319012 | 9.882084 |
| H | 42.046814 | 58.536297 | 9.912217 |
| H | 42.994606 | 58.655926 | 11.408193 |
| C | 40.202663 | 52.097595 | 8.344875 |
| O | 38.621292 | 52.947262 | 6.283992 |
| C | 40.061573 | 53.592136 | 8.080352 |
| O | 37.841293 | 60.241253 | 6.162622 |
| C | 39.051132 | 53.870728 | 6.956232 |
| O | 36.088249 | 59.361359 | 5.099339 |
| C | 38.611526 | 55.275986 | 6.681623 |
| C | 39.298054 | 56.411461 | 7.148456 |
| C | 38.854496 | 57.690811 | 6.812329 |
| C | 37.697834 | 57.866680 | 6.038785 |
| C | 37.006145 | 56.731915 | 5.583415 |
| C | 37.464752 | 55.454094 | 5.886586 |
| C | 37.187836 | 59.267082 | 5.745986 |
| H | 40.531921 | 51.565842 | 7.440470 |
| H | 39.237560 | 51.673798 | 8.659588 |
| H | 40.935986 | 51.928921 | 9.145734 |
| H | 41.034737 | 53.983025 | 7.705905 |
| H | 40.338860 | 54.292477 | 9.894256 |
| H | 40.171417 | 56.296078 | 7.789645 |
| H | 39.388874 | 58.577068 | 7.156948 |
| H | 36.094662 | 56.859627 | 4.997906 |
| H | 36.932812 | 54.570198 | 5.530344 |
| H | 37.542709 | 50.177109 | -0.990228 |
| H | 33.525459 | 60.456722 | -0.711815 |
| H | 39.350071 | 61.597149 | 1.588737 |
| H | 34.770603 | 62.982559 | 7.708793 |
| H | 35.268429 | 61.820530 | 8.976870 |
| H | 32.134914 | 59.300056 | 8.160469 |
| H | 32.616238 | 57.652500 | 8.655717 |
| H | 49.344772 | 50.802006 | 1.752220 |
| H | 33.743748 | 49.757702 | 3.578251 |
| H | 44.098236 | 55.422428 | 15.125783 |
| H | 45.367546 | 54.554111 | 14.214587 |
| H | 47.380219 | 62.395309 | 13.262120 |
| H | 42.255894 | 58.765083 | 5.913096 |
| H | 43.618622 | 58.880585 | 4.790996 |
| H | 40.272198 | 62.517960 | 2.806546 |

**The C–C Bond Cleavage Reaction**

**$^{2}$RE2$_{r2}$**

| | | | |
|---|---|---|---|
| C | 57.255424 | 57.277332 | 44.055023 |
| N | 55.934376 | 56.877281 | 43.610706 |
| C | 55.805126 | 56.164021 | 42.385487 |
| N | 56.062710 | 54.788277 | 42.491196 |
| N | 54.730194 | 56.639130 | 41.588474 |
| H | 57.933655 | 57.542126 | 43.215714 |
| H | 57.769611 | 56.527416 | 44.656853 |
| H | 55.274708 | 57.650089 | 43.616909 |
| H | 55.450119 | 54.302380 | 43.160301 |
| H | 56.073563 | 54.289639 | 41.597973 |
| H | 54.962582 | 57.490089 | 41.077065 |
| H | 54.354755 | 55.962570 | 40.914570 |
| C | 51.197948 | 59.806034 | 40.405910 |
| C | 50.858288 | 58.333706 | 40.679546 |
| O | 50.330456 | 57.708874 | 39.540127 |
| H | 52.136040 | 59.900944 | 39.860058 |
| H | 51.757992 | 57.814018 | 41.063210 |
| H | 50.109028 | 58.298378 | 41.500793 |
| H | 50.867512 | 56.931118 | 39.271942 |
| C | 48.097618 | 54.812386 | 39.916145 |
| C | 46.749508 | 55.324738 | 40.439167 |
| C | 45.564556 | 54.578587 | 39.813957 |
| C | 46.692207 | 55.263130 | 41.971519 |
| H | 48.263500 | 53.758644 | 40.197502 |
| H | 48.911312 | 55.412670 | 40.321857 |
| H | 46.667568 | 56.388668 | 40.145893 |
| H | 44.597961 | 54.971939 | 40.174831 |
| H | 45.573837 | 54.664639 | 38.715309 |
| H | 45.598644 | 53.503250 | 40.063179 |
| H | 45.746853 | 55.678413 | 42.362957 |
| H | 46.769970 | 54.219967 | 42.325836 |
| H | 47.523518 | 55.828159 | 42.423691 |
| C | 42.866039 | 55.100857 | 31.033169 |
| N | 42.472744 | 53.918556 | 30.296532 |
| C | 43.209030 | 52.751011 | 30.395903 |
| N | 44.092308 | 52.586014 | 31.313320 |
| N | 42.901852 | 51.831276 | 29.390135 |

H 43.084881 54.860004 32.073025
H 42.032066 55.796101 31.136862
H 42.132408 54.100330 29.358456
H 44.182121 52.827263 33.097050
H 44.499371 51.649349 31.296284
H 41.922623 51.805756 29.118790
H 43.269802 50.899658 29.553896
C 54.367989 47.756012 45.433987
C 54.527714 46.522087 44.539223
C 53.849415 46.603413 43.153156
C 52.319054 46.684132 43.249649
C 54.422318 47.733234 42.286942
H 53.308708 47.981197 45.636963
H 55.606586 46.326874 44.392998
H 54.131100 45.638432 45.073582
H 54.087860 45.651226 42.641510
H 51.993053 47.617020 43.740360
H 51.859138 46.678925 42.250164
H 51.911640 45.839886 43.833462
H 55.514439 47.630280 42.163757
H 53.950832 47.744560 41.294289
H 54.230911 48.722607 42.735214
C 54.529842 55.506153 36.210140
C 53.169716 55.744568 35.549755
O 52.416019 56.727318 36.210461
H 54.347084 55.273880 37.258987
H 53.301907 56.083542 34.504055
H 52.623795 54.777355 35.492554
H 52.149162 56.365444 37.082272
C 56.908268 51.685867 38.400688
C 55.618755 52.134872 39.071377
O 55.783852 53.372074 39.727627
H 57.693871 51.452332 39.119247
H 54.826508 52.212666 38.305393
H 55.281738 51.346680 39.775898
H 54.950226 53.895226 39.623577
C 54.525871 49.441116 36.476128
C 53.514153 50.470165 35.973946
H 54.147022 48.927910 37.360958
H 53.861378 50.966038 35.050991
H 52.545273 49.991528 35.764458
H 53.332069 51.255749 36.724606
C 48.229858 41.837029 42.197102
C 47.787483 43.111267 41.459702
C 46.967766 44.022011 42.382889
C 48.996258 43.857616 40.880459
H 48.929134 42.116959 42.984894
H 47.133072 42.812790 40.617668
H 47.571785 44.350021 43.247154
H 46.637051 44.929760 41.853527
H 46.073055 43.508308 42.775620
H 49.531879 43.241791 40.139252
H 48.716351 44.805359 40.395718
H 49.714336 44.112453 41.679920
C 40.012138 52.850037 44.798904
N 40.009674 52.426262 43.419388
C 40.759071 51.378887 42.911327
N 41.673069 50.723438 43.556335
N 40.440910 51.056866 41.618717
H 39.443935 53.779961 44.842972
H 41.006981 53.113976 45.157074
H 39.135159 52.574627 42.929314
H 41.734474 50.980881 44.539497
H 42.831078 49.805267 42.811459
H 40.180161 51.836605 41.024029
H 41.086128 50.384266 41.170277
C 45.410130 49.034962 33.573986
C 45.557190 47.553921 33.924198
S 47.231686 46.875187 33.595036
H 46.134930 49.633060 34.126026
H 44.851837 46.951492 33.323364
H 45.294437 47.385143 34.979061
C 44.201950 47.988987 38.940838
C 50.813389 42.076687 34.496880
C 52.354050 49.453983 31.989529
C 46.647514 52.328415 36.722420
N 47.093624 46.900288 36.785225
C 44.520489 48.321232 40.405399
C 50.648071 40.745728 34.358006
C 53.074661 49.353466 30.855768
C 45.219112 52.455097 36.159241
N 48.983112 45.154129 35.465187
C 43.383163 49.039135 41.105095
C 45.160694 53.025692 34.751183
N 48.297562 49.151028 35.545872
C 46.477959 49.235943 37.177277
C 47.143578 44.452168 36.929863
C 50.909920 45.095722 33.963669

| | | | |
|---|---|---|---|
| C | 49.996986 | 49.840710 | 33.917149 |
| C | 44.668598 | 44.808754 | 38.748966 |
| C | 48.287537 | 41.603954 | 36.476410 |
| C | 53.054745 | 46.289982 | 32.070187 |
| C | 49.019913 | 52.698563 | 34.541542 |
| C | 46.323906 | 47.862858 | 37.371033 |
| C | 48.256168 | 44.201221 | 36.124729 |
| C | 50.980900 | 46.453300 | 33.666077 |
| C | 48.975155 | 50.092201 | 34.824074 |
| O | 49.802677 | 47.345322 | 37.020630 |
| O | 43.601967 | 49.279633 | 42.378040 |
| O | 42.359421 | 49.362328 | 40.511021 |
| C | 45.283119 | 47.257442 | 38.197353 |
| C | 48.798164 | 42.879372 | 35.881416 |
| C | 51.955315 | 47.035805 | 32.760151 |
| C | 48.484753 | 51.430801 | 35.133343 |
| O | 50.807777 | 48.159908 | 36.956078 |
| O | 45.924469 | 53.860035 | 34.318344 |
| O | 44.138058 | 52.528049 | 34.048904 |
| C | 45.466629 | 45.901615 | 38.105804 |
| C | 49.884682 | 43.056332 | 35.041927 |
| C | 51.679508 | 48.388176 | 32.719761 |
| C | 47.495583 | 51.269051 | 36.071445 |
| C | 46.613640 | 45.700275 | 37.231571 |
| C | 49.972183 | 44.494904 | 34.800159 |
| C | 50.550686 | 48.594612 | 33.629662 |
| C | 47.397430 | 49.831646 | 36.316231 |
| N | 50.146557 | 47.414234 | 34.167339 |
| Fe | 48.585491 | 47.159534 | 35.434380 |
| H | 43.946720 | 48.924896 | 38.421291 |
| H | 43.274857 | 47.393345 | 38.920582 |
| H | 51.771912 | 42.487923 | 34.159241 |
| H | 52.256008 | 50.454174 | 32.426579 |
| H | 46.569965 | 52.131187 | 37.803928 |
| H | 47.143517 | 53.305313 | 36.626289 |
| H | 45.417892 | 48.957336 | 40.488800 |
| H | 44.763599 | 47.414562 | 40.984776 |
| H | 49.722527 | 40.232784 | 34.626114 |
| H | 51.450462 | 40.128498 | 33.943687 |
| H | 53.199486 | 48.410774 | 30.318375 |
| H | 53.549507 | 50.236954 | 30.419767 |
| H | 44.688152 | 51.492855 | 36.174213 |
| H | 44.638630 | 53.146637 | 36.797840 |
| H | 45.804379 | 49.896721 | 37.722977 |

| | | | |
|---|---|---|---|
| H | 46.652058 | 43.588848 | 37.378410 |
| H | 51.634464 | 44.445404 | 33.475410 |
| H | 50.421764 | 50.706463 | 33.411255 |
| H | 44.331169 | 44.060608 | 38.011444 |
| H | 45.255283 | 44.265354 | 39.510132 |
| H | 43.773335 | 45.207161 | 39.249973 |
| H | 47.724907 | 41.782967 | 37.404694 |
| H | 47.614983 | 41.060802 | 35.786587 |
| H | 49.119034 | 40.921703 | 36.716225 |
| H | 53.939026 | 46.932655 | 31.936367 |
| H | 53.367405 | 45.404518 | 32.644573 |
| H | 52.756271 | 45.936935 | 31.065521 |
| H | 50.021629 | 52.936668 | 34.939766 |
| H | 49.116856 | 52.619343 | 33.446423 |
| H | 48.352211 | 53.545963 | 34.741650 |
| C | 48.630367 | 48.900158 | 41.211803 |
| O | 49.245491 | 47.192776 | 39.675995 |
| C | 49.350330 | 48.568501 | 39.899845 |
| O | 51.674622 | 48.268265 | 40.414886 |
| C | 50.813541 | 49.036545 | 40.029327 |
| O | 51.570965 | 55.405376 | 38.631920 |
| C | 51.131569 | 50.490158 | 39.810669 |
| O | 53.562233 | 54.881908 | 39.526188 |
| C | 50.468929 | 51.299458 | 38.869965 |
| C | 50.852047 | 52.631805 | 38.688839 |
| C | 51.905247 | 53.184269 | 39.431545 |
| C | 52.551735 | 52.381893 | 40.386253 |
| C | 52.174709 | 51.054600 | 40.567768 |
| C | 52.378189 | 54.612244 | 39.182552 |
| H | 47.584599 | 48.565517 | 41.142052 |
| H | 48.650478 | 49.978516 | 41.431618 |
| H | 49.108368 | 48.358383 | 42.043423 |
| H | 48.867924 | 49.135956 | 39.083481 |
| H | 49.498730 | 47.041149 | 38.736732 |
| H | 49.681850 | 50.880562 | 38.240925 |
| H | 50.352676 | 53.262741 | 37.951263 |
| H | 53.374130 | 52.812286 | 40.960388 |
| H | 52.691528 | 50.422096 | 41.292381 |
| H | 54.863194 | 47.603344 | 46.408028 |
| H | 54.813782 | 48.654099 | 44.978638 |
| H | 57.163910 | 58.168037 | 44.703423 |
| H | 51.306248 | 60.367496 | 41.351608 |
| H | 50.392471 | 60.270267 | 39.814575 |
| H | 39.569786 | 52.117867 | 45.503483 |

H 47.380203 41.303017 42.655975
H 48.749733 41.137794 41.521263
H 55.082146 54.674671 35.739773
H 55.149837 56.417313 36.167141
H 56.738575 50.775341 37.808979
H 57.282856 52.474743 37.727657
H 55.495758 49.895233 36.719631
H 54.709534 48.665623 35.712372
H 43.745319 55.619537 30.611492
H 45.603565 49.198040 32.498901
H 44.398563 49.416077 33.805340
H 48.163189 54.884785 38.819748

**$^{2}$TS1$_{r2}$**

C 57.255333 57.277294 44.055035
N 55.964882 56.869743 43.487926
C 55.725525 56.057720 42.398144
N 56.422379 54.920288 42.252365
N 54.780880 56.400936 41.542213
H 57.957874 57.619350 43.274670
H 57.769672 56.527431 44.656818
H 55.235687 57.564053 43.602886
H 56.721397 54.444588 43.094807
H 56.278057 54.313217 41.414314
H 54.534470 57.382980 41.474232
H 54.311020 55.744461 40.793968
C 51.197861 59.805801 40.405754
C 50.822021 58.340511 40.657555
O 50.318844 57.738358 39.504292
H 52.136093 59.901039 39.860168
H 51.704983 57.795265 41.053604
H 50.061131 58.308571 41.468189
H 50.863819 56.955723 39.267143
C 48.097431 54.812557 39.916225
C 46.772243 55.272846 40.542091
C 45.558849 54.528187 39.972649
C 46.818558 55.151337 42.071838
H 48.301094 53.751144 40.132076
H 48.911610 55.412540 40.321732
H 46.649990 56.346294 40.298962
H 44.616962 54.888916 40.422443
H 45.481270 54.656284 38.881310
H 45.629700 53.445496 40.174133
H 45.893177 55.530579 42.540104
H 46.939404 54.097591 42.378197
H 47.666912 55.716854 42.492493
C 42.866039 55.100773 31.033274
N 42.478382 53.915730 30.297152
C 43.204941 52.742500 30.428951
N 44.051208 52.580544 31.378843
N 42.929329 51.818859 29.416351
H 43.084839 54.860039 32.073044
H 42.032097 55.796146 31.136784
H 42.172489 54.091801 29.346157
H 43.989113 52.800938 33.163593
H 44.460224 51.644291 31.390684
H 41.957069 51.791973 29.120327
H 43.287083 50.887245 29.603712
C 54.367916 47.755993 45.433800
C 54.466606 46.583439 44.447758
C 53.769394 46.757847 43.076351
C 52.247639 46.921459 43.182041
C 54.375072 47.881001 42.223122
H 53.321411 47.988491 45.687996
H 55.536354 46.363358 44.269058
H 54.052811 45.680397 44.934704
H 53.951672 45.812378 42.531269
H 51.975437 47.849716 43.713543
H 51.800041 46.989250 42.178062
H 51.785313 46.077202 43.723019
H 55.460049 47.737553 42.072483
H 53.879421 47.918240 41.239738
H 54.228943 48.869022 42.693535
C 54.529800 55.506214 36.210178
C 53.248394 56.203709 35.739964
O 52.859215 57.220055 36.636532
H 54.347088 55.273827 37.258976
H 53.387985 56.673553 34.748783
H 52.448895 55.444927 35.619366
H 52.545948 56.772472 37.447777
C 56.908184 51.685921 38.400665
C 55.659676 52.140934 39.138966
O 55.849380 53.373245 39.847275
H 57.693867 51.452309 39.119247
H 54.830395 52.268867 38.427189
H 55.339825 51.346596 39.837204
H 55.029644 53.919117 39.708660
C 54.525913 49.441101 36.476151

C 53.544590 50.532806 36.052376
H 54.147003 48.927921 37.360954
H 53.842106 51.012047 35.103600
H 52.535030 50.112770 35.932163
H 53.463047 51.319965 36.819729
C 48.230038 41.837257 42.196861
C 47.615047 43.134224 41.637177
C 46.675125 43.778152 42.664963
C 48.709522 44.118931 41.200493
H 48.928951 42.116814 42.985107
H 47.011734 42.876125 40.746540
H 47.226418 44.055458 43.581284
H 46.222038 44.699421 42.265301
H 45.858376 43.095737 42.959488
H 49.335201 43.701862 40.395351
H 48.312592 45.083023 40.842506
H 49.377769 44.350571 42.049530
C 40.011944 52.850075 44.799057
N 40.009464 52.417339 43.423615
C 40.716621 51.319527 42.955227
N 41.598980 50.652817 43.630669
N 40.389294 50.963741 41.674553
H 39.444019 53.780014 44.842957
H 41.007019 53.113934 45.156998
H 39.138851 52.577225 42.929871
H 41.670067 50.952183 44.601215
H 42.779400 49.704113 42.895748
H 40.157795 51.732948 41.054199
H 41.025772 50.264374 41.249748
C 45.410858 49.034599 33.573891
C 45.653980 47.571011 33.967186
S 47.393845 47.014252 33.753662
H 46.134418 49.633408 34.126320
H 45.016766 46.900135 33.359203
H 45.343796 47.421066 35.014729
C 44.170006 47.976006 39.002197
C 51.453903 42.660828 34.910480
C 52.322979 50.082836 32.240620
C 46.286587 52.485954 36.850861
N 47.196419 47.109943 36.944260
C 44.483788 48.287197 40.473000
C 51.441078 41.317734 34.770069
C 53.103691 50.023075 31.141762
C 44.853542 52.515446 36.283134
N 49.281033 45.546295 35.751141
C 43.334251 48.957127 41.191875
C 44.764370 53.128380 34.895962
N 48.252781 49.475632 35.732407
C 46.381382 49.390209 37.301151
C 47.408127 44.671227 37.062496
C 51.282879 45.676018 34.357811
C 49.901337 50.305176 34.122654
C 44.773861 44.833015 38.698269
C 48.835560 41.933838 36.681751
C 53.340851 47.012310 32.468582
C 48.615810 53.062241 34.665466
C 46.333057 48.009361 37.492496
C 48.598392 44.524105 36.344921
C 51.228958 47.027988 34.021519
C 48.836853 50.468613 35.000179
O 49.867344 47.697357 37.261311
O 43.556541 49.197884 42.467922
O 42.289406 49.251694 40.618877
C 45.295609 47.321575 38.257877
C 49.273872 43.257954 36.137093
C 52.159321 47.670071 33.111717
C 48.201733 51.754620 35.270447
O 50.419441 48.928589 37.603523
O 45.369453 54.120686 34.546719
O 43.910954 52.476341 34.103878
C 45.559410 45.982346 38.143581
C 50.401272 43.542854 35.385559
C 51.749107 48.987179 33.006729
C 47.221748 51.506245 36.197266
C 46.769566 45.872482 37.339031
C 50.368359 44.990017 35.154697
C 50.587765 49.113640 33.888691
C 47.266895 50.066437 36.461040
N 50.291637 47.920265 34.465569
Fe 48.774090 47.528416 35.753433
H 43.873283 48.912506 38.505966
H 43.272015 47.337055 38.967876
H 52.385803 43.168682 34.633629
H 52.080021 51.084560 32.614220
H 46.217186 52.260456 37.926998
H 46.703365 53.501968 36.776398
H 45.368671 48.940762 40.562382
H 44.757835 47.375690 41.030678

| | | | |
|---|---|---|---|
| H | 50.559399 | 40.708752 | 34.978970 |
| H | 52.331245 | 40.789555 | 34.414921 |
| H | 53.373329 | 49.080734 | 30.659904 |
| H | 53.481289 | 50.939701 | 30.678667 |
| H | 44.410351 | 51.510593 | 36.261318 |
| H | 44.220650 | 53.140606 | 36.941090 |
| H | 45.632504 | 49.989357 | 37.819950 |
| H | 46.950649 | 43.765602 | 37.462524 |
| H | 52.088505 | 45.093159 | 33.912319 |
| H | 50.244686 | 51.195324 | 33.596130 |
| H | 44.549244 | 44.082134 | 37.920929 |
| H | 45.306904 | 44.301147 | 39.506077 |
| H | 43.812321 | 45.172333 | 39.114658 |
| H | 48.178967 | 42.051086 | 37.557083 |
| H | 48.279530 | 41.335644 | 35.934921 |
| H | 49.702908 | 41.328312 | 36.992764 |
| H | 54.166134 | 47.729805 | 32.334370 |
| H | 53.718987 | 46.176407 | 33.076988 |
| H | 53.102505 | 46.603508 | 31.468290 |
| H | 49.592190 | 53.398376 | 35.058552 |
| H | 48.722248 | 52.982639 | 33.570534 |
| H | 47.873177 | 53.848450 | 34.857372 |
| C | 49.138142 | 49.186314 | 41.328762 |
| O | 48.855820 | 47.486168 | 39.705837 |
| C | 49.240467 | 48.835293 | 39.847641 |
| O | 51.588604 | 48.274879 | 39.757526 |
| C | 50.704647 | 49.052879 | 39.304585 |
| O | 51.805237 | 55.590355 | 38.830521 |
| C | 51.085648 | 50.562023 | 39.279587 |
| O | 53.686657 | 54.872368 | 39.799740 |
| C | 50.462551 | 51.552780 | 38.497295 |
| C | 50.901432 | 52.880363 | 38.530239 |
| C | 51.974503 | 53.269382 | 39.354389 |
| C | 52.568874 | 52.287060 | 40.168030 |
| C | 52.137775 | 50.963108 | 40.120045 |
| C | 52.493736 | 54.674126 | 39.331623 |
| H | 48.113869 | 48.991322 | 41.690792 |
| H | 49.381947 | 50.244423 | 41.516586 |
| H | 49.832481 | 48.551552 | 41.899765 |
| H | 48.555828 | 49.497124 | 39.278122 |
| H | 49.152515 | 47.242924 | 38.800831 |
| H | 49.651848 | 51.266804 | 37.827484 |
| H | 50.431370 | 53.634609 | 37.895184 |
| H | 53.394581 | 52.576035 | 40.821846 |
| H | 52.634495 | 50.188046 | 40.707600 |
| H | 54.894112 | 47.524704 | 46.375755 |
| H | 54.816784 | 48.674896 | 45.021515 |
| H | 57.058151 | 58.126392 | 44.724377 |
| H | 51.304863 | 60.357903 | 41.357552 |
| H | 50.403145 | 60.289047 | 39.816322 |
| H | 39.570503 | 52.121487 | 45.508621 |
| H | 47.470051 | 41.153965 | 42.612549 |
| H | 48.799782 | 41.294334 | 41.424721 |
| H | 54.751591 | 54.583721 | 35.649624 |
| H | 55.397148 | 56.185459 | 36.158592 |
| H | 56.705341 | 50.778561 | 37.816586 |
| H | 57.275433 | 52.469673 | 37.717266 |
| H | 55.529472 | 49.841808 | 36.682220 |
| H | 54.632076 | 48.677212 | 35.686985 |
| H | 43.746048 | 55.620647 | 30.613743 |
| H | 45.613136 | 49.186348 | 32.497986 |
| H | 44.385708 | 49.384182 | 33.801880 |
| H | 48.099438 | 54.940643 | 38.823021 |

**$^{2}$INT1$_{r2}$**

| | | | |
|---|---|---|---|
| C | 57.255280 | 57.277023 | 44.054752 |
| N | 55.919209 | 56.917088 | 43.601837 |
| C | 55.667179 | 56.286991 | 42.383003 |
| N | 56.547039 | 55.290466 | 42.066696 |
| N | 54.689377 | 56.618130 | 41.597542 |
| H | 57.921768 | 57.525433 | 43.209499 |
| H | 57.769672 | 56.527599 | 44.657028 |
| H | 55.205204 | 57.595669 | 43.835995 |
| H | 56.933395 | 54.764133 | 42.842121 |
| H | 56.389290 | 54.732269 | 41.221447 |
| H | 54.239704 | 57.486778 | 41.885712 |
| H | 53.822239 | 55.539593 | 40.677292 |
| C | 51.197952 | 59.805584 | 40.405624 |
| C | 50.796837 | 58.339031 | 40.557194 |
| O | 50.411015 | 57.803448 | 39.320507 |
| H | 52.136131 | 59.901215 | 39.860260 |
| H | 51.638142 | 57.769093 | 40.999718 |
| H | 49.962185 | 58.270222 | 41.289352 |
| H | 50.844398 | 56.939480 | 39.194859 |
| C | 48.097363 | 54.812492 | 39.916409 |
| C | 46.821220 | 55.084789 | 40.726723 |
| C | 45.614693 | 54.302071 | 40.197151 |
| C | 47.048798 | 54.798862 | 42.217625 |

| | | | |
|---|---|---|---|
| H | 48.390072 | 53.751175 | 39.965973 |
| H | 48.911488 | 55.412579 | 40.321651 |
| H | 46.589096 | 56.163471 | 40.627064 |
| H | 44.699078 | 54.537506 | 40.767471 |
| H | 45.421665 | 54.529949 | 39.137070 |
| H | 45.786175 | 53.214981 | 40.277210 |
| H | 46.152969 | 55.034084 | 42.818726 |
| H | 47.289604 | 53.733986 | 42.380867 |
| H | 47.888878 | 55.392212 | 42.615959 |
| C | 42.866024 | 55.100807 | 31.033213 |
| N | 42.476261 | 53.918083 | 30.294184 |
| C | 43.202576 | 52.744457 | 30.414682 |
| N | 44.056740 | 52.577126 | 31.357948 |
| N | 42.919300 | 51.825939 | 29.399967 |
| H | 43.084808 | 54.859997 | 32.073040 |
| H | 42.032112 | 55.796165 | 31.136808 |
| H | 42.161354 | 54.098503 | 29.346983 |
| H | 43.898113 | 52.661835 | 33.158016 |
| H | 44.463612 | 51.639709 | 31.357178 |
| H | 41.946510 | 51.804520 | 29.105452 |
| H | 43.273994 | 50.892094 | 29.581663 |
| C | 54.367855 | 47.755985 | 45.433777 |
| C | 54.288254 | 46.604046 | 44.419098 |
| C | 53.547054 | 46.874168 | 43.085899 |
| C | 52.049759 | 47.170448 | 43.251740 |
| C | 54.211296 | 47.953968 | 42.220066 |
| H | 53.365494 | 48.095974 | 45.742908 |
| H | 55.318672 | 46.275837 | 44.182152 |
| H | 53.805382 | 45.738560 | 44.911270 |
| H | 53.618473 | 45.928024 | 42.516319 |
| H | 51.880116 | 48.108345 | 43.808990 |
| H | 51.577450 | 47.291656 | 42.261105 |
| H | 51.535007 | 46.359875 | 43.797619 |
| H | 55.272549 | 47.721622 | 42.018208 |
| H | 53.673973 | 48.037720 | 41.260765 |
| H | 54.172485 | 48.945023 | 42.706799 |
| C | 54.529690 | 55.506321 | 36.210239 |
| C | 53.252102 | 56.157505 | 35.680882 |
| O | 52.844604 | 57.248604 | 36.479065 |
| H | 54.347198 | 55.273701 | 37.258968 |
| H | 53.407135 | 56.551643 | 34.660576 |
| H | 52.454655 | 55.390984 | 35.604904 |
| H | 52.555531 | 56.892002 | 37.334560 |
| C | 56.908226 | 51.685871 | 38.400513 |
| C | 55.662270 | 52.189259 | 39.102360 |
| O | 55.885910 | 53.466740 | 39.694965 |
| H | 57.693836 | 51.452362 | 39.119297 |
| H | 54.837597 | 52.261227 | 38.373844 |
| H | 55.339474 | 51.450378 | 39.860950 |
| H | 55.000957 | 53.830463 | 39.889881 |
| C | 54.526005 | 49.441101 | 36.476208 |
| C | 53.612450 | 50.609055 | 36.109032 |
| H | 54.146938 | 48.927940 | 37.360939 |
| H | 53.905571 | 51.080090 | 35.155945 |
| H | 52.573185 | 50.261364 | 36.012878 |
| H | 53.614094 | 51.387516 | 36.888508 |
| C | 48.230019 | 41.837704 | 42.196785 |
| C | 47.708263 | 43.130543 | 41.545029 |
| C | 46.832085 | 43.922604 | 42.524078 |
| C | 48.869225 | 43.990261 | 41.025749 |
| H | 48.929024 | 42.116512 | 42.985153 |
| H | 47.073948 | 42.851547 | 40.681824 |
| H | 47.412895 | 44.230091 | 43.411839 |
| H | 46.446903 | 44.840607 | 42.052967 |
| H | 45.970814 | 43.328041 | 42.876785 |
| H | 49.435921 | 43.470318 | 40.236359 |
| H | 48.535748 | 44.956982 | 40.616650 |
| H | 49.575253 | 44.220627 | 41.843372 |
| C | 40.011959 | 52.849991 | 44.799114 |
| N | 40.009357 | 52.419327 | 43.422306 |
| C | 40.691673 | 51.302528 | 42.959309 |
| N | 41.547646 | 50.609310 | 43.641003 |
| N | 40.365059 | 50.959816 | 41.674335 |
| H | 39.444019 | 53.780014 | 44.842945 |
| H | 41.007004 | 53.114025 | 45.156963 |
| H | 39.140766 | 52.589207 | 42.927883 |
| H | 41.616547 | 50.902588 | 44.613564 |
| H | 42.691540 | 49.585285 | 42.918209 |
| H | 40.174774 | 51.738651 | 41.051704 |
| H | 40.975002 | 50.233845 | 41.257915 |
| C | 45.411289 | 49.034317 | 33.573895 |
| C | 45.617973 | 47.568344 | 33.975292 |
| S | 47.352959 | 46.982349 | 33.816998 |
| H | 46.134106 | 49.633698 | 34.126415 |
| H | 44.991779 | 46.908840 | 33.344219 |
| H | 45.270802 | 47.419659 | 35.010929 |
| C | 43.977676 | 47.661987 | 39.082561 |
| C | 51.531410 | 42.756626 | 34.981586 |

C 52.101357 50.264481 32.471745
C 45.864124 52.285278 36.997063
N 47.022587 46.958515 36.996235
C 44.337444 48.063148 40.521355
C 51.585270 41.417206 34.820698
C 53.288143 50.410923 31.844727
C 44.467793 52.274235 36.337975
N 49.206657 45.520363 35.826431
C 43.217464 48.795395 41.226002
C 44.438747 52.960617 34.982620
N 48.024155 49.405064 35.894745
C 46.110462 49.193832 37.400772
C 47.345310 44.531582 37.071712
C 51.242249 45.774727 34.500427
C 49.683788 50.351212 34.362431
C 44.732121 44.545944 38.744976
C 48.901009 41.870052 36.657730
C 53.226307 47.228699 32.601418
C 48.244385 53.028084 34.917332
C 46.120621 47.808495 37.560028
C 48.555408 44.455345 36.376854
C 51.145149 47.133507 34.199413
C 48.588394 50.443634 35.211174
O 49.610451 47.633862 37.451145
O 43.439400 49.029999 42.502968
O 42.197563 49.146729 40.640121
C 45.114544 47.061882 38.311638
C 49.293575 43.225742 36.157898
C 52.056835 47.833225 33.314075
C 47.883865 51.690945 35.491287
O 50.232204 48.844215 37.784409
O 44.922997 54.052742 34.763828
O 43.792736 52.249046 34.059845
C 45.437233 45.738403 38.174107
C 50.426979 43.579315 35.446709
C 51.599251 49.138218 33.241501
C 46.882759 51.371937 36.373142
C 46.649769 45.696827 37.364944
C 50.335491 45.029171 35.249527
C 50.419346 49.193424 34.105453
C 46.988052 49.929951 36.604134
N 50.169743 47.977211 34.655594
Fe 48.624084 47.478809 35.873634
H 43.589596 48.552975 38.566151
H 43.130733 46.956356 39.116604
H 52.443855 43.311966 34.733295
H 51.406647 51.108566 32.392750
H 45.736374 52.021309 38.058968
H 46.236111 53.321102 36.981834
H 45.228703 48.714657 40.535122
H 44.628090 47.191559 41.130890
H 50.730003 40.763645 35.002342
H 52.506809 40.937752 34.477020
H 54.076389 49.657326 31.878714
H 53.508663 51.327908 31.290352
H 44.081760 51.250961 36.238029
H 43.767105 52.835548 36.983253
H 45.324436 49.746513 37.916096
H 46.923546 43.597809 37.444527
H 52.088123 45.238419 34.072632
H 50.011230 51.273312 33.883011
H 44.620255 43.740459 37.999771
H 45.276482 44.116428 39.604885
H 43.723175 44.809948 39.099876
H 48.201733 41.935097 37.504665
H 48.410526 41.259956 35.875690
H 49.784203 41.303635 36.996525
H 54.187298 47.503056 33.075081
H 53.174034 46.130283 32.583305
H 53.275951 47.577785 31.556194
H 49.234039 53.368507 35.270428
H 48.294704 52.991425 33.815861
H 47.501175 53.794781 35.175865
C 48.878563 49.261074 41.442001
O 48.467194 47.569748 39.838413
C 48.961792 48.884644 39.965622
O 51.281670 48.179539 39.931931
C 50.444286 48.986561 39.421425
O 51.931206 55.502022 39.066559
C 50.900059 50.479057 39.396481
O 53.485813 54.612659 40.417236
C 50.374634 51.492844 38.570927
C 50.858883 52.800957 38.643669
C 51.875401 53.140446 39.558739
C 52.389996 52.127228 40.397289
C 51.916225 50.825554 40.301254
C 52.397186 54.518158 39.641617
H 47.851513 49.095669 41.811501

H 49.145073 50.316971 41.612099
H 49.563213 48.623550 42.023640
H 48.330696 49.589626 39.383457
H 48.795372 47.271420 38.960266
H 49.597553 51.238491 37.850086
H 50.460239 53.576828 37.985607
H 53.166504 52.378670 41.121494
H 52.318169 50.019787 40.917503
H 54.907375 47.442242 46.343853
H 54.897713 48.630074 45.019943
H 57.177795 58.173592 44.688824
H 51.319538 60.287884 41.392017
H 50.418320 60.350555 39.849590
H 39.568642 52.121075 45.507141
H 47.420216 41.231148 42.637501
H 48.776218 41.210545 41.472538
H 54.783997 54.578850 35.673130
H 55.382240 56.203011 36.161232
H 56.679882 50.764790 37.848366
H 57.287704 52.438560 37.690392
H 55.568485 49.749088 36.651409
H 54.542526 48.693443 35.664818
H 43.745495 55.620846 30.612860
H 45.628136 49.174576 32.498905
H 44.389935 49.402470 33.783470
H 47.974197 55.082401 38.855446

**$^{2}$TS2$_{r2}$**

C 57.255447 57.277195 44.054813
N 55.897598 56.937382 43.657772
C 55.585709 56.290218 42.459293
N 56.461670 55.298592 42.111088
N 54.566315 56.601833 41.724529
H 57.897217 57.484066 43.179832
H 57.769566 56.527493 44.656986
H 55.197960 57.618664 43.923607
H 56.898209 54.789616 42.870518
H 56.276451 54.730656 41.278343
H 54.125607 57.470612 42.026497
H 53.724651 55.549641 40.628220
C 51.197941 59.805965 40.405884
C 50.790161 58.340111 40.543636
O 50.428555 57.816830 39.298061
H 52.136055 59.901009 39.860096
H 51.622787 57.766567 40.998955
H 49.943378 58.271618 41.263481
H 50.846531 56.939342 39.184689
C 48.097664 54.812325 39.916187
C 46.990063 54.650280 40.967392
C 45.806713 53.829052 40.440926
C 47.547447 54.038975 42.260250
H 48.513584 53.836250 39.618584
H 48.911282 55.412739 40.321827
H 46.610020 55.662003 41.213234
H 45.000786 53.752167 41.191219
H 45.378773 54.275780 39.528374
H 46.124123 52.802807 40.189827
H 46.769733 53.951736 43.038704
H 47.948570 53.028076 42.074127
H 48.370083 54.649284 42.668514
C 42.866024 55.100861 31.033159
N 42.467072 53.921719 30.292681
C 43.215698 52.759239 30.370104
N 44.112000 52.598141 31.274424
N 42.903118 51.845230 29.359688
H 43.084896 54.860008 32.073025
H 42.032070 55.796104 31.136864
H 42.114346 54.110043 29.360447
H 44.037937 52.746738 33.067425
H 44.531631 51.666950 31.250860
H 41.917816 51.808311 29.111795
H 43.281815 50.916267 29.516100
C 54.367996 47.756001 45.434002
C 54.351254 46.607277 44.416256
C 53.746201 46.905849 43.023388
C 52.251240 47.250309 43.063751
C 54.522484 47.970581 42.236187
H 53.351982 48.120235 45.656796
H 55.388329 46.248676 44.274105
H 53.799057 45.755959 44.856171
H 53.838940 45.961910 42.454498
H 52.066360 48.196732 43.600700
H 51.857491 47.371876 42.041103
H 51.667667 46.460251 43.567162
H 55.594246 47.718781 42.152386
H 54.105690 48.061218 41.220608
H 54.446533 48.964531 42.709934
C 54.529762 55.506187 36.210167

| | | | |
|---|---|---|---|
| C | 53.226971 | 56.102474 | 35.675331 |
| O | 52.765602 | 57.163372 | 36.481899 |
| H | 54.347160 | 55.273834 | 37.258991 |
| H | 53.369102 | 56.511761 | 34.658585 |
| H | 52.468117 | 55.298328 | 35.588451 |
| H | 52.500771 | 56.780502 | 37.336136 |
| C | 56.908211 | 51.685959 | 38.400757 |
| C | 55.670025 | 52.219151 | 39.096828 |
| O | 55.899624 | 53.509724 | 39.654484 |
| H | 57.693913 | 51.452297 | 39.119194 |
| H | 54.840897 | 52.274284 | 38.372265 |
| H | 55.347778 | 51.500031 | 39.874321 |
| H | 55.013351 | 53.905735 | 39.785942 |
| C | 54.525921 | 49.441116 | 36.476158 |
| C | 53.605732 | 50.586796 | 36.062592 |
| H | 54.146984 | 48.927914 | 37.360943 |
| H | 53.940037 | 51.060871 | 35.124199 |
| H | 52.580528 | 50.218422 | 35.909981 |
| H | 53.549744 | 51.366470 | 36.838314 |
| C | 48.229828 | 41.837116 | 42.197102 |
| C | 47.698143 | 43.114155 | 41.523720 |
| C | 46.785004 | 43.900558 | 42.472912 |
| C | 48.849773 | 43.991199 | 41.013885 |
| H | 48.929161 | 42.116898 | 42.984894 |
| H | 47.089436 | 42.810688 | 40.650658 |
| H | 47.338215 | 44.228935 | 43.370750 |
| H | 46.391861 | 44.805012 | 41.981647 |
| H | 45.926949 | 43.293926 | 42.811497 |
| H | 49.453243 | 43.467224 | 40.255402 |
| H | 48.497395 | 44.932751 | 40.563873 |
| H | 49.525200 | 44.266075 | 41.843491 |
| C | 40.012135 | 52.849998 | 44.798901 |
| N | 40.006836 | 52.414158 | 43.425560 |
| C | 40.754200 | 51.349476 | 42.945724 |
| N | 41.669872 | 50.716869 | 43.610474 |
| N | 40.430565 | 50.987144 | 41.667606 |
| H | 39.443954 | 53.779976 | 44.842960 |
| H | 41.006973 | 53.113991 | 45.157082 |
| H | 39.123875 | 52.537872 | 42.943466 |
| H | 41.729954 | 51.005474 | 44.584862 |
| H | 42.853859 | 49.793804 | 42.874889 |
| H | 40.154106 | 51.742729 | 41.049740 |
| H | 41.071594 | 50.294586 | 41.238667 |
| C | 45.410122 | 49.034958 | 33.573994 |
| C | 45.628838 | 47.561028 | 33.934086 |
| S | 47.353924 | 46.971767 | 33.694851 |
| H | 46.134937 | 49.633060 | 34.126015 |
| H | 44.984013 | 46.915203 | 33.308491 |
| H | 45.320553 | 47.385162 | 34.976734 |
| C | 44.129265 | 47.919800 | 39.015114 |
| C | 51.290829 | 42.507603 | 34.867355 |
| C | 52.281097 | 49.928421 | 32.236481 |
| C | 46.240330 | 52.392670 | 36.789555 |
| N | 47.087826 | 47.003872 | 36.888313 |
| C | 44.503445 | 48.302395 | 40.454247 |
| C | 51.273190 | 41.162128 | 34.746750 |
| C | 53.499832 | 50.014603 | 31.663448 |
| C | 44.810257 | 52.430901 | 36.210957 |
| N | 49.148167 | 45.417030 | 35.697163 |
| C | 43.373516 | 48.996029 | 41.181900 |
| C | 44.739029 | 53.065411 | 34.832195 |
| N | 48.179581 | 49.360512 | 35.684757 |
| C | 46.323437 | 49.301392 | 37.275894 |
| C | 47.256538 | 44.559593 | 36.995396 |
| C | 51.182129 | 45.529568 | 34.347469 |
| C | 49.847137 | 50.173714 | 34.084980 |
| C | 44.693882 | 44.769363 | 38.733242 |
| C | 48.635193 | 41.802952 | 36.588524 |
| C | 53.247158 | 46.839687 | 32.438370 |
| C | 48.562057 | 52.936569 | 34.590431 |
| C | 46.254318 | 47.919643 | 37.459614 |
| C | 48.442688 | 44.397919 | 36.276543 |
| C | 51.153168 | 46.884670 | 34.018661 |
| C | 48.770084 | 50.347622 | 34.943108 |
| O | 49.754486 | 47.546196 | 37.261974 |
| O | 43.629738 | 49.286331 | 42.439243 |
| O | 42.313187 | 49.263988 | 40.623932 |
| C | 45.225487 | 47.247967 | 38.245766 |
| C | 49.100914 | 43.126732 | 36.066303 |
| C | 52.104465 | 47.519562 | 33.126358 |
| C | 48.144421 | 51.635410 | 35.206989 |
| O | 50.390270 | 48.812305 | 37.472591 |
| O | 45.308403 | 54.092335 | 34.525215 |
| O | 43.950451 | 52.389061 | 33.995182 |
| C | 45.461857 | 45.902550 | 38.122765 |
| C | 50.245907 | 43.401474 | 35.334991 |
| C | 51.712101 | 48.843666 | 33.021069 |
| C | 47.168270 | 51.400558 | 36.144093 |

C 46.644394 45.770393 37.287651
C 50.237724 44.850178 35.114498
C 50.529907 48.977501 33.868500
C 47.205212 49.964771 36.418819
N 50.217918 47.786213 34.448803
Fe 48.669868 47.403046 35.697281
H 43.793449 48.828529 38.492424
H 43.242332 47.266235 39.052727
H 52.223312 43.006111 34.576958
H 51.617821 50.789852 32.097340
H 46.164738 52.177258 37.867252
H 46.665741 53.404804 36.710922
H 45.383102 48.968689 40.474827
H 44.809128 47.421394 41.043583
H 50.392849 40.557125 34.971027
H 52.159908 40.627476 34.393162
H 54.260807 49.239426 31.767445
H 53.777779 50.902748 31.088415
H 44.367836 51.426357 36.170979
H 44.172047 53.048531 36.870071
H 45.588589 49.910175 37.802757
H 46.785915 43.659744 37.392239
H 51.996544 44.941593 33.927418
H 50.213528 51.062763 33.572746
H 44.540806 43.944592 38.017517
H 45.212116 44.343288 39.611103
H 43.699905 45.099289 39.074703
H 47.929279 41.918537 37.424549
H 48.125523 41.201721 35.811951
H 49.484608 41.199875 36.950237
H 54.210327 47.023228 32.950279
H 53.107491 45.749432 32.391640
H 53.360825 47.206409 31.404644
H 49.540596 53.271759 34.978764
H 48.665981 52.848145 33.496086
H 47.824661 53.728428 34.779694
C 49.123669 49.433437 41.193752
O 48.729206 47.680153 39.646698
C 49.037216 48.995213 39.765347
O 51.559601 48.059990 39.356129
C 50.910530 48.961052 38.796364
O 51.836525 55.507088 38.992718
C 51.174580 50.408176 38.995628
O 53.486252 54.640114 40.267662
C 50.580452 51.451828 38.233498
C 50.932144 52.776344 38.434818
C 51.901447 53.141830 39.408554
C 52.442806 52.103199 40.214462
C 52.089146 50.781971 40.014156
C 52.356213 54.514858 39.528820
H 48.157543 49.283772 41.718990
H 49.406441 50.493336 41.281174
H 49.871155 48.811710 41.715202
H 48.509876 49.671360 39.070885
H 48.981640 47.407829 38.715454
H 49.868408 51.192162 37.451496
H 50.498627 53.561764 37.811119
H 53.169926 52.362000 40.986221
H 52.531750 49.984158 40.612354
H 54.818512 47.430511 46.387169
H 54.952518 48.616825 45.070217
H 57.224072 58.192013 44.666641
H 51.322124 60.280769 41.395863
H 50.421043 60.359150 39.853615
H 39.570511 52.123955 45.511295
H 47.421459 41.231686 42.641754
H 48.777260 41.203114 41.479958
H 54.825764 54.586910 35.680168
H 55.353275 56.237690 36.163147
H 56.664627 50.756264 37.869930
H 57.295155 52.417912 37.673264
H 55.557224 49.773987 36.669823
H 54.580288 48.682285 35.676395
H 43.746723 55.617790 30.612200
H 45.598190 49.203037 32.498016
H 44.390133 49.388573 33.814175
H 47.728985 55.316841 39.008770

**$^{2}$INT2$_{r2}$**

C 57.255463 57.277378 44.055016
N 55.945133 56.862122 43.595196
C 55.836288 56.080048 42.409649
N 56.103771 54.713760 42.603451
N 54.759899 56.494022 41.581337
H 57.943459 57.556160 43.226879
H 57.769569 56.527390 44.656853
H 55.290409 57.637287 43.541527
H 55.479370 54.270489 43.290993

| | | | |
|---|---|---|---|
| H | 56.105301 | 54.161392 | 41.743694 |
| H | 54.989323 | 57.299278 | 40.999866 |
| H | 54.364517 | 55.768143 | 40.972523 |
| C | 51.197952 | 59.805916 | 40.405910 |
| C | 50.887516 | 58.324158 | 40.657841 |
| O | 50.461082 | 57.698784 | 39.478046 |
| H | 52.136040 | 59.901039 | 39.860077 |
| H | 51.780746 | 57.832203 | 41.091106 |
| H | 50.092056 | 58.257126 | 41.433262 |
| H | 50.989277 | 56.893772 | 39.282845 |
| C | 48.097649 | 54.812340 | 39.916199 |
| C | 46.780998 | 55.107166 | 40.645664 |
| C | 45.608540 | 54.326809 | 40.038708 |
| C | 46.909561 | 54.830315 | 42.149647 |
| H | 48.373180 | 53.749016 | 40.008633 |
| H | 48.911289 | 55.412727 | 40.321823 |
| H | 46.564842 | 56.186295 | 40.523460 |
| H | 44.657131 | 54.555237 | 40.550411 |
| H | 45.480755 | 54.563660 | 38.970089 |
| H | 45.777355 | 53.238876 | 40.116196 |
| H | 45.981316 | 55.081757 | 42.692230 |
| H | 47.125378 | 53.763515 | 42.334789 |
| H | 47.731667 | 55.415138 | 42.593983 |
| C | 42.866024 | 55.100853 | 31.033192 |
| N | 42.513210 | 53.893024 | 30.317192 |
| C | 43.059959 | 52.670963 | 30.674543 |
| N | 43.580975 | 52.471165 | 31.832575 |
| N | 43.008549 | 51.730656 | 29.643284 |
| H | 43.084896 | 54.860004 | 32.073025 |
| H | 42.032070 | 55.796108 | 31.136839 |
| H | 42.404129 | 54.012363 | 29.316639 |
| H | 42.956963 | 52.299698 | 33.628674 |
| H | 43.942463 | 51.519672 | 31.923944 |
| H | 42.210457 | 51.805634 | 29.019106 |
| H | 43.164326 | 50.774048 | 29.944090 |
| C | 54.367981 | 47.756001 | 45.433983 |
| C | 53.496708 | 46.625225 | 44.872684 |
| C | 52.572361 | 46.977547 | 43.683624 |
| C | 51.512527 | 48.029545 | 44.039616 |
| C | 53.342243 | 47.377926 | 42.417351 |
| H | 53.760254 | 48.593533 | 45.813164 |
| H | 54.154423 | 45.791748 | 44.562973 |
| H | 52.870205 | 46.222733 | 45.690681 |
| H | 52.028011 | 46.044724 | 43.444557 |
| H | 51.965794 | 49.017307 | 44.229431 |
| H | 50.794559 | 48.152805 | 43.213371 |
| H | 50.943604 | 47.742401 | 44.940842 |
| H | 54.086266 | 46.611401 | 42.140259 |
| H | 52.654564 | 47.504959 | 41.565849 |
| H | 53.881065 | 48.331329 | 42.552837 |
| C | 54.529915 | 55.506187 | 36.210159 |
| C | 53.180428 | 55.467705 | 35.489635 |
| O | 52.242481 | 56.347794 | 36.049511 |
| H | 54.347004 | 55.273819 | 37.258961 |
| H | 53.302326 | 55.757553 | 34.428219 |
| H | 52.811214 | 54.418312 | 35.480663 |
| H | 52.054272 | 56.056686 | 36.967331 |
| C | 56.908215 | 51.685814 | 38.400600 |
| C | 55.573029 | 51.889057 | 39.104553 |
| O | 55.621857 | 52.957867 | 40.019562 |
| H | 57.693874 | 51.452370 | 39.119255 |
| H | 54.790577 | 52.072186 | 38.348518 |
| H | 55.287338 | 50.945278 | 39.615608 |
| H | 54.825943 | 53.530293 | 39.870598 |
| C | 54.525997 | 49.441193 | 36.476257 |
| C | 53.605392 | 50.578331 | 36.040817 |
| H | 54.146931 | 48.927860 | 37.360889 |
| H | 53.997498 | 51.101807 | 35.152851 |
| H | 52.602386 | 50.205242 | 35.787853 |
| H | 53.477978 | 51.326275 | 36.837776 |
| C | 48.229839 | 41.837173 | 42.197083 |
| C | 47.853291 | 43.108898 | 41.420029 |
| C | 47.039978 | 44.072529 | 42.293350 |
| C | 49.103031 | 43.800858 | 40.860340 |
| H | 48.929153 | 42.116859 | 42.984913 |
| H | 47.218456 | 42.817226 | 40.562042 |
| H | 47.613876 | 44.358849 | 43.193199 |
| H | 46.806103 | 44.999416 | 41.747456 |
| H | 46.096397 | 43.611629 | 42.635132 |
| H | 49.663795 | 43.138332 | 40.180065 |
| H | 48.849335 | 44.718670 | 40.309231 |
| H | 49.785042 | 44.091709 | 41.679211 |
| C | 40.012127 | 52.850029 | 44.798901 |
| N | 40.004784 | 52.427490 | 43.418552 |
| C | 40.468288 | 51.200806 | 42.982708 |
| N | 41.064091 | 50.324158 | 43.730556 |
| N | 40.219128 | 50.951103 | 41.660381 |
| H | 39.443947 | 53.779968 | 44.842964 |

| | | | |
|---|---|---|---|
| H | 41.006981 | 53.113964 | 45.157074 |
| H | 39.274330 | 52.823959 | 42.840263 |
| H | 41.100231 | 50.590149 | 44.712296 |
| H | 41.866577 | 49.034554 | 43.173042 |
| H | 40.264221 | 51.757690 | 41.046352 |
| H | 40.690716 | 50.109688 | 41.286884 |
| C | 45.410179 | 49.034912 | 33.573994 |
| C | 45.082489 | 47.737453 | 34.308037 |
| S | 46.504467 | 46.628181 | 34.644096 |
| H | 46.134892 | 49.633099 | 34.126034 |
| H | 44.381413 | 47.133026 | 33.703896 |
| H | 44.564419 | 47.953884 | 35.254524 |
| C | 42.930347 | 46.457691 | 39.629230 |
| C | 51.269726 | 43.239910 | 35.372208 |
| C | 50.630852 | 50.945385 | 33.596916 |
| C | 43.885452 | 51.423546 | 37.757591 |
| N | 46.112282 | 46.432636 | 37.655136 |
| C | 43.276634 | 46.956890 | 41.046726 |
| C | 51.522991 | 41.948959 | 35.077675 |
| C | 51.322769 | 51.152130 | 32.460617 |
| C | 42.722809 | 51.356808 | 36.738251 |
| N | 48.524094 | 45.493240 | 36.440475 |
| C | 42.358986 | 48.043327 | 41.563587 |
| C | 42.911186 | 52.292740 | 35.553509 |
| N | 46.677879 | 49.101654 | 36.753906 |
| C | 44.725391 | 48.418613 | 38.055561 |
| C | 46.880348 | 44.107239 | 37.611889 |
| C | 50.454105 | 46.183289 | 35.108150 |
| C | 48.252277 | 50.473804 | 35.474251 |
| C | 44.274197 | 43.535686 | 39.184879 |
| C | 48.874348 | 41.798683 | 37.019066 |
| C | 52.162704 | 48.094166 | 33.373573 |
| C | 46.388824 | 52.808388 | 36.206924 |
| C | 45.017738 | 47.058517 | 38.170780 |
| C | 48.078445 | 44.294239 | 36.920692 |
| C | 50.138962 | 47.528564 | 34.940643 |
| C | 47.118279 | 50.296078 | 36.256687 |
| O | 48.556126 | 47.572575 | 38.284321 |
| O | 42.423328 | 48.219982 | 42.861900 |
| O | 41.657814 | 48.717484 | 40.812359 |
| C | 44.151127 | 46.093407 | 38.840279 |
| C | 49.014126 | 43.231602 | 36.608887 |
| C | 50.914379 | 48.442661 | 34.122341 |
| C | 46.189331 | 51.371075 | 36.582142 |
| O | 49.051525 | 48.911381 | 38.444416 |
| O | 43.167336 | 53.472858 | 35.659023 |
| O | 42.739239 | 51.684052 | 34.376614 |
| C | 44.742847 | 44.868393 | 38.686214 |
| C | 50.048431 | 43.824780 | 35.907509 |
| C | 50.283283 | 49.669277 | 34.211010 |
| C | 45.159378 | 50.785465 | 37.273552 |
| C | 45.974937 | 45.103622 | 37.947067 |
| C | 49.705719 | 45.243698 | 35.812870 |
| C | 49.143803 | 49.468700 | 35.102890 |
| C | 45.499737 | 49.366058 | 37.388931 |
| N | 49.070862 | 48.171967 | 35.506447 |
| Fe | 47.593601 | 47.301750 | 36.590187 |
| H | 42.367580 | 47.252930 | 39.119045 |
| H | 42.245277 | 45.596344 | 39.696030 |
| H | 52.083237 | 43.952778 | 35.193939 |
| H | 50.283131 | 51.835152 | 34.134109 |
| H | 43.548203 | 50.939445 | 38.687881 |
| H | 44.063503 | 52.481518 | 38.004353 |
| H | 44.292870 | 47.390465 | 41.050255 |
| H | 43.318081 | 46.140911 | 41.784622 |
| H | 50.774998 | 41.160645 | 35.182144 |
| H | 52.503994 | 41.649750 | 34.697472 |
| H | 51.681087 | 50.332996 | 31.833096 |
| H | 51.536469 | 52.168438 | 32.118542 |
| H | 42.571743 | 50.329590 | 36.378956 |
| H | 41.791279 | 51.677948 | 37.238548 |
| H | 43.807610 | 48.768555 | 38.528374 |
| H | 46.632484 | 43.090546 | 37.916294 |
| H | 51.352879 | 45.826305 | 34.607376 |
| H | 48.444016 | 51.479553 | 35.102703 |
| H | 44.207840 | 42.793789 | 38.370396 |
| H | 44.958179 | 43.118931 | 39.944122 |
| H | 43.278072 | 43.609695 | 39.646957 |
| H | 48.221973 | 41.687668 | 37.897785 |
| H | 48.446579 | 41.173481 | 36.213223 |
| H | 49.855309 | 41.365921 | 37.273537 |
| H | 52.841000 | 48.959614 | 33.319752 |
| H | 52.708996 | 47.269653 | 33.856205 |
| H | 51.951046 | 47.780037 | 32.334812 |
| H | 47.312969 | 53.212894 | 36.653149 |
| H | 46.475986 | 52.935051 | 35.114483 |
| H | 45.547764 | 53.434299 | 36.531578 |
| C | 46.837158 | 48.396770 | 42.730442 |

| | | | |
|---|---|---|---|
| O | 47.233612 | 47.259739 | 40.633556 |
| C | 47.524811 | 48.331070 | 41.411777 |
| O | 50.771347 | 48.060661 | 39.667061 |
| C | 50.204823 | 48.987324 | 39.135292 |
| O | 51.675320 | 55.341297 | 38.679588 |
| C | 50.694756 | 50.412498 | 39.172924 |
| O | 53.533405 | 54.568005 | 39.671124 |
| C | 50.140797 | 51.429558 | 38.376575 |
| C | 50.674042 | 52.718410 | 38.403915 |
| C | 51.773300 | 53.025543 | 39.218700 |
| C | 52.308144 | 52.015095 | 40.035015 |
| C | 51.777096 | 50.724461 | 40.012012 |
| C | 52.373016 | 54.428837 | 39.195045 |
| H | 45.733536 | 48.535290 | 42.655315 |
| H | 47.228111 | 49.235943 | 43.328194 |
| H | 46.989285 | 47.466003 | 43.307880 |
| H | 47.823132 | 49.251976 | 40.895874 |
| H | 47.699577 | 47.336189 | 39.751472 |
| H | 49.299133 | 51.196247 | 37.727242 |
| H | 50.256165 | 53.516056 | 37.786846 |
| H | 53.162258 | 52.252392 | 40.671551 |
| H | 52.201054 | 49.934093 | 40.634178 |
| H | 54.987740 | 47.394512 | 46.271839 |
| H | 55.051872 | 48.165661 | 44.673206 |
| H | 57.143120 | 58.161705 | 44.708084 |
| H | 51.296841 | 60.361313 | 41.355728 |
| H | 50.391087 | 60.263500 | 39.811337 |
| H | 39.557205 | 52.118656 | 45.494152 |
| H | 47.352264 | 41.355198 | 42.660572 |
| H | 48.729961 | 41.095043 | 41.551853 |
| H | 55.244053 | 54.774040 | 35.795898 |
| H | 54.974533 | 56.513760 | 36.154251 |
| H | 56.856884 | 50.856693 | 37.679306 |
| H | 57.203068 | 52.601715 | 37.862244 |
| H | 55.540848 | 49.796986 | 36.704659 |
| H | 54.622345 | 48.681396 | 35.680859 |
| H | 43.737904 | 55.625404 | 30.603497 |
| H | 45.846771 | 48.821362 | 32.582855 |
| H | 44.498886 | 49.648209 | 33.437912 |
| H | 48.027393 | 55.049908 | 38.843056 |

**TS3$_{r2}$**

| | | | |
|---|---|---|---|
| C | 57.255493 | 57.277462 | 44.055096 |
| N | 55.932331 | 56.906010 | 43.569008 |
| C | 55.675949 | 56.222252 | 42.399376 |
| N | 56.515320 | 55.226479 | 42.057804 |
| N | 54.648869 | 56.546799 | 41.649292 |
| H | 57.928383 | 57.548809 | 43.222912 |
| H | 57.769558 | 56.527336 | 44.656796 |
| H | 55.187881 | 57.538975 | 43.833076 |
| H | 57.066883 | 54.799809 | 42.791332 |
| H | 56.366245 | 54.645123 | 41.212856 |
| H | 54.168911 | 57.411236 | 41.877163 |
| H | 54.173149 | 55.848240 | 40.823845 |
| C | 51.197960 | 59.805935 | 40.405930 |
| C | 50.744732 | 58.350250 | 40.521000 |
| O | 50.315609 | 57.853180 | 39.286381 |
| H | 52.136036 | 59.901028 | 39.860065 |
| H | 51.569153 | 57.735970 | 40.937038 |
| H | 49.919777 | 58.296444 | 41.264454 |
| H | 50.817814 | 57.040634 | 39.080257 |
| C | 48.097664 | 54.812309 | 39.916206 |
| C | 46.852680 | 54.923313 | 40.806198 |
| C | 45.664764 | 54.161961 | 40.208721 |
| C | 47.152336 | 54.450737 | 42.235352 |
| H | 48.441803 | 53.768093 | 39.840778 |
| H | 48.911255 | 55.412750 | 40.321815 |
| H | 46.573566 | 55.994301 | 40.860466 |
| H | 44.760010 | 54.263531 | 40.832859 |
| H | 45.422642 | 54.531052 | 39.199398 |
| H | 45.891041 | 53.086514 | 40.117729 |
| H | 46.275055 | 54.567657 | 42.895058 |
| H | 47.435162 | 53.384174 | 42.243782 |
| H | 47.987583 | 55.018547 | 42.679546 |
| C | 42.866024 | 55.100857 | 31.033190 |
| N | 42.514332 | 53.893112 | 30.316301 |
| C | 43.148197 | 52.695377 | 30.622658 |
| N | 43.782608 | 52.532635 | 31.726093 |
| N | 43.038090 | 51.748878 | 29.600470 |
| H | 43.084896 | 54.860004 | 32.073025 |
| H | 42.032070 | 55.796104 | 31.136841 |
| H | 42.365482 | 54.021912 | 29.321482 |
| H | 43.188969 | 52.424938 | 33.504475 |
| H | 44.171024 | 51.591339 | 31.817472 |
| H | 42.154282 | 51.759964 | 29.098480 |
| H | 43.296032 | 50.808266 | 29.883207 |
| C | 54.367985 | 47.756001 | 45.433987 |
| C | 53.549873 | 46.674717 | 44.714821 |
| C | 52.692734 | 47.123707 | 43.506897 |

| | | | |
|---|---|---|---|
| C | 51.586376 | 48.117043 | 43.888000 |
| C | 53.526424 | 47.652702 | 42.331062 |
| H | 53.724167 | 48.546490 | 45.853783 |
| H | 54.240376 | 45.881660 | 44.372276 |
| H | 52.882397 | 46.190849 | 45.452106 |
| H | 52.186306 | 46.208820 | 43.148792 |
| H | 52.001652 | 49.096340 | 44.184570 |
| H | 50.909409 | 48.288620 | 43.035538 |
| H | 50.977364 | 47.742226 | 44.728703 |
| H | 54.308994 | 46.931973 | 42.036884 |
| H | 52.888882 | 47.836205 | 41.450390 |
| H | 54.028122 | 48.603767 | 42.584743 |
| C | 54.529964 | 55.506168 | 36.210140 |
| C | 53.347919 | 56.371574 | 35.767384 |
| O | 53.112137 | 57.431679 | 36.668098 |
| H | 54.346962 | 55.273834 | 37.258957 |
| H | 53.538342 | 56.823395 | 34.776917 |
| H | 52.450439 | 55.730522 | 35.653473 |
| H | 52.777435 | 57.031754 | 37.491756 |
| C | 56.908173 | 51.685623 | 38.400616 |
| C | 55.721947 | 52.311543 | 39.114555 |
| O | 56.024094 | 53.610622 | 39.630417 |
| H | 57.693920 | 51.452503 | 39.119251 |
| H | 54.877117 | 52.397102 | 38.413834 |
| H | 55.382961 | 51.636787 | 39.923820 |
| H | 55.154514 | 54.075825 | 39.667931 |
| C | 54.525993 | 49.441231 | 36.476254 |
| C | 53.715191 | 50.692139 | 36.149815 |
| H | 54.146931 | 48.927841 | 37.360878 |
| H | 54.114750 | 51.219265 | 35.267090 |
| H | 52.669724 | 50.433914 | 35.928707 |
| H | 53.701279 | 51.402905 | 36.990150 |
| C | 48.229839 | 41.837162 | 42.197083 |
| C | 47.954319 | 43.082890 | 41.337662 |
| C | 47.199978 | 44.154751 | 42.134583 |
| C | 49.254704 | 43.653923 | 40.758022 |
| H | 48.929153 | 42.116863 | 42.984909 |
| H | 47.312710 | 42.783680 | 40.487690 |
| H | 47.774487 | 44.445637 | 43.033035 |
| H | 47.055256 | 45.066105 | 41.533504 |
| H | 46.214878 | 43.789249 | 42.474979 |
| H | 49.775436 | 42.917263 | 40.123634 |
| H | 49.063950 | 44.552971 | 40.152878 |
| H | 49.945721 | 43.946079 | 41.569485 |
| C | 40.012119 | 52.850029 | 44.798904 |
| N | 39.999153 | 52.412609 | 43.423492 |
| C | 40.467068 | 51.174217 | 43.008785 |
| N | 41.115482 | 50.334415 | 43.752602 |
| N | 40.156750 | 50.876789 | 41.708828 |
| H | 39.443951 | 53.779972 | 44.842964 |
| H | 41.006985 | 53.113964 | 45.157074 |
| H | 39.221470 | 52.754372 | 42.871426 |
| H | 41.195274 | 50.637062 | 44.721462 |
| H | 42.041126 | 49.075909 | 43.123756 |
| H | 40.165993 | 51.663357 | 41.067375 |
| H | 40.629391 | 50.032520 | 41.338787 |
| C | 45.410149 | 49.034939 | 33.573978 |
| C | 45.297588 | 47.652927 | 34.225605 |
| S | 46.909309 | 46.793285 | 34.384834 |
| H | 46.134914 | 49.633072 | 34.126038 |
| H | 44.624096 | 47.010010 | 33.625694 |
| H | 44.828312 | 47.748722 | 35.218552 |
| C | 43.232254 | 46.768555 | 39.444191 |
| C | 51.599056 | 43.324684 | 35.399807 |
| C | 51.045231 | 50.971603 | 33.325531 |
| C | 44.363480 | 51.711559 | 37.585129 |
| N | 46.423420 | 46.669094 | 37.473522 |
| C | 43.571690 | 47.271244 | 40.858799 |
| C | 51.888767 | 42.016907 | 35.220436 |
| C | 51.881439 | 51.174400 | 32.283707 |
| C | 43.131886 | 51.619419 | 36.653328 |
| N | 48.871174 | 45.657440 | 36.329594 |
| C | 42.524151 | 48.168068 | 41.475376 |
| C | 43.250912 | 52.504318 | 35.423409 |
| N | 47.068989 | 49.292423 | 36.565113 |
| C | 45.111038 | 48.689659 | 37.897507 |
| C | 47.139194 | 44.326138 | 37.441776 |
| C | 50.853340 | 46.285408 | 35.054031 |
| C | 48.655357 | 50.590008 | 35.221153 |
| C | 44.489422 | 43.809044 | 38.967323 |
| C | 49.097530 | 41.956181 | 36.941471 |
| C | 52.585758 | 48.121666 | 33.247158 |
| C | 46.833549 | 52.986435 | 35.897400 |
| C | 45.354935 | 47.318657 | 38.005798 |
| C | 48.373177 | 44.475571 | 36.799797 |
| C | 50.554550 | 47.629166 | 34.834286 |
| C | 47.524742 | 50.459068 | 36.020317 |
| O | 48.852760 | 47.661968 | 38.094498 |

| | | | |
|---|---|---|---|
| O | 42.683140 | 48.365726 | 42.769150 |
| O | 41.634113 | 48.696217 | 40.815014 |
| C | 44.453922 | 46.374901 | 38.667904 |
| C | 49.292500 | 43.384571 | 36.537636 |
| C | 51.340134 | 48.503010 | 33.984837 |
| C | 46.623096 | 51.565746 | 36.328068 |
| O | 49.420872 | 49.251057 | 38.434052 |
| O | 43.542633 | 53.682407 | 35.458477 |
| O | 42.973179 | 51.857254 | 34.289856 |
| C | 45.004803 | 45.134434 | 38.495262 |
| C | 50.375446 | 43.942921 | 35.878407 |
| C | 50.704357 | 49.733589 | 34.004269 |
| C | 45.599293 | 51.027744 | 37.066002 |
| C | 46.249016 | 45.343700 | 37.757648 |
| C | 50.068783 | 45.370903 | 35.753849 |
| C | 49.550358 | 49.567863 | 34.894447 |
| C | 45.907936 | 49.603539 | 37.210701 |
| N | 49.476753 | 48.294312 | 35.357731 |
| Fe | 47.968819 | 47.485058 | 36.449673 |
| H | 42.685001 | 47.568192 | 38.922501 |
| H | 42.528233 | 45.921986 | 39.515293 |
| H | 52.400280 | 44.033039 | 35.157120 |
| H | 50.543415 | 51.863216 | 33.719383 |
| H | 44.079269 | 51.281864 | 38.559525 |
| H | 44.565678 | 52.778046 | 37.765903 |
| H | 44.502720 | 47.865368 | 40.826126 |
| H | 43.797009 | 46.447201 | 41.553925 |
| H | 51.167233 | 41.217644 | 35.398804 |
| H | 52.878880 | 41.714661 | 34.865486 |
| H | 52.410675 | 50.363487 | 31.780088 |
| H | 52.039913 | 52.183529 | 31.891729 |
| H | 42.946899 | 50.581394 | 36.345638 |
| H | 42.240009 | 51.973736 | 37.202801 |
| H | 44.205784 | 49.073238 | 38.370579 |
| H | 46.854713 | 43.316349 | 37.740349 |
| H | 51.760269 | 45.903080 | 34.586258 |
| H | 48.845909 | 51.578789 | 34.803528 |
| H | 44.395538 | 43.086754 | 38.137104 |
| H | 45.158005 | 43.348072 | 39.715481 |
| H | 43.496349 | 43.907192 | 39.433506 |
| H | 48.326473 | 41.852066 | 37.718925 |
| H | 48.791080 | 41.319065 | 36.090004 |
| H | 50.031849 | 41.525398 | 37.339775 |
| H | 53.328396 | 48.936165 | 33.273930 |
| H | 53.057705 | 47.225163 | 33.676548 |
| H | 52.388229 | 47.904785 | 32.180153 |
| H | 47.776657 | 53.395760 | 36.300690 |
| H | 46.893456 | 53.074310 | 34.798580 |
| H | 46.009697 | 53.636929 | 36.221653 |
| C | 47.258404 | 48.347359 | 42.536716 |
| O | 47.748543 | 47.301018 | 40.412445 |
| C | 47.957611 | 48.357632 | 41.218353 |
| O | 51.276165 | 48.431904 | 39.482037 |
| C | 50.575401 | 49.318336 | 39.014301 |
| O | 52.047016 | 55.779701 | 38.908142 |
| C | 51.036995 | 50.776875 | 39.119064 |
| O | 53.725555 | 54.875145 | 40.075836 |
| C | 50.556877 | 51.791134 | 38.272331 |
| C | 51.045135 | 53.095226 | 38.379913 |
| C | 52.020378 | 53.417217 | 39.340694 |
| C | 52.470951 | 52.404972 | 40.210041 |
| C | 51.989445 | 51.104424 | 40.096973 |
| C | 52.596630 | 54.794312 | 39.431637 |
| H | 46.146805 | 48.419655 | 42.460655 |
| H | 47.592247 | 49.193249 | 43.161758 |
| H | 47.460907 | 47.412689 | 43.093426 |
| H | 48.182217 | 49.315090 | 40.730461 |
| H | 48.166958 | 47.456127 | 39.481960 |
| H | 49.807426 | 51.531731 | 37.522774 |
| H | 50.686752 | 53.879997 | 37.709949 |
| H | 53.215481 | 52.658504 | 40.966625 |
| H | 52.350384 | 50.306927 | 40.749073 |
| H | 54.944878 | 47.323109 | 46.268974 |
| H | 55.086437 | 48.243370 | 44.754486 |
| H | 57.145988 | 58.165073 | 44.694508 |
| H | 51.335884 | 60.257732 | 41.405621 |
| H | 50.432182 | 60.390759 | 39.871498 |
| H | 39.561680 | 52.124508 | 45.504662 |
| H | 47.312420 | 41.442211 | 42.666393 |
| H | 48.691963 | 41.026489 | 41.608021 |
| H | 54.616512 | 54.571991 | 35.632896 |
| H | 55.479446 | 56.063095 | 36.149681 |
| H | 56.602135 | 50.746101 | 37.922306 |
| H | 57.310600 | 52.363987 | 37.631580 |
| H | 55.593334 | 49.663681 | 36.638313 |
| H | 54.476990 | 48.716705 | 35.646606 |
| H | 43.740471 | 55.627132 | 30.609318 |
| H | 45.789452 | 48.939941 | 32.540253 |

H 44.441620 49.574097 33.555008
H 47.897079 55.174423 38.895508

**[2]PR2$_{r2}$**

C 57.255379 57.277203 44.054886
N 55.953156 56.918201 43.513973
C 55.855091 56.432335 42.211414
N 56.622448 55.341789 41.956390
N 55.110760 56.987816 41.302368
H 57.958279 57.584713 43.260010
H 57.769627 56.527496 44.656937
H 55.240810 57.613605 43.704205
H 56.832733 54.740036 42.743458
H 56.593658 54.872292 41.042919
H 54.719978 57.883904 41.584854
H 54.525318 56.135090 40.258728
C 51.197922 59.805939 40.405781
C 50.725857 58.362457 40.586449
O 50.247856 57.797722 39.395580
H 52.136097 59.901020 39.860168
H 51.540878 57.754646 41.027130
H 49.904743 58.359104 41.330582
H 50.907379 57.159702 39.066269
C 48.097538 54.812527 39.916042
C 46.776119 55.582241 40.063389
C 45.611687 54.859924 39.374496
C 46.460052 55.854473 41.540478
H 48.060024 53.837772 40.428345
H 48.911373 55.412525 40.321953
H 46.909569 56.562267 39.566193
H 44.669579 55.429058 39.468475
H 45.807446 54.710922 38.300220
H 45.449451 53.864246 39.821907
H 45.536591 56.448982 41.658051
H 46.323463 54.908089 42.092308
H 47.280277 56.406567 42.029713
C 42.866001 55.100822 31.033228
N 42.516819 53.869972 30.361313
C 43.153873 52.685596 30.716869
N 43.883282 52.589977 31.768120
N 42.924778 51.664997 29.788176
H 43.084911 54.860023 32.073025
H 42.032078 55.796120 31.136814
H 42.348225 53.949295 29.364326
H 44.009949 52.742306 33.771957
H 44.213192 51.633003 31.911690
H 41.981209 51.640968 29.410130
H 43.224365 50.747169 30.102835
C 54.367985 47.756001 45.433971
C 52.955109 47.204033 45.221748
C 52.013340 48.077583 44.364986
C 51.692398 49.431965 45.013176
C 52.509373 48.263809 42.924717
H 54.353416 48.737228 45.936615
H 53.031319 46.205425 44.753452
H 52.484669 47.038502 46.209480
H 51.058231 47.524746 44.300949
H 52.591202 50.070911 45.085472
H 50.950142 49.964195 44.398548
H 51.291424 49.306351 46.035172
H 52.694874 47.293350 42.433155
H 51.754688 48.818092 42.346157
H 53.450001 48.842232 42.890091
C 54.529694 55.506187 36.210155
C 53.530670 56.583675 35.787560
O 53.545769 57.689590 36.661758
H 54.347229 55.273846 37.259007
H 53.774506 56.962395 34.778442
H 52.517586 56.136517 35.718742
H 53.203152 57.360741 37.512821
C 56.908226 51.685940 38.400738
C 55.883884 52.617062 39.019253
O 56.461102 53.893959 39.292065
H 57.693897 51.452312 39.119213
H 55.032990 52.741844 38.328468
H 55.469604 52.167309 39.942299
H 55.704353 54.507244 39.254044
C 54.525887 49.441101 36.476128
C 53.733192 50.717350 36.200268
H 54.147007 48.927925 37.360958
H 54.092163 51.232903 35.293510
H 52.665630 50.491413 36.053852
H 53.795425 51.431087 37.036896
C 48.229874 41.837097 42.197067
C 47.858261 43.029713 41.304611
C 47.202812 44.143669 42.129173
C 49.059158 43.546852 40.501446
H 48.929119 42.116917 42.984924

H 47.109749 42.670647 40.572758
H 47.896332 44.506550 42.910091
H 46.933479 45.008404 41.504807
H 46.292408 43.782005 42.640038
H 49.558441 42.722870 39.962955
H 48.762096 44.292961 39.745983
H 49.809010 44.011967 41.166744
C 40.012150 52.850040 44.798920
N 39.975422 52.388847 43.430531
C 40.293957 51.125195 42.984787
N 40.920483 50.304852 43.879456
N 39.988762 50.696091 41.795773
H 39.443928 53.779957 44.842957
H 41.006981 53.113972 45.157074
H 39.612537 53.021690 42.731731
H 41.565998 50.747852 44.524418
H 41.281353 49.426601 43.463287
H 39.420815 51.359035 41.269581
H 40.820683 49.564083 41.053741
C 45.410259 49.034863 33.574020
C 45.151402 47.619957 34.099957
S 46.555233 46.447891 33.947620
H 46.134842 49.633163 34.126026
H 44.310772 47.193211 33.520882
H 44.811520 47.660385 35.143433
C 42.835831 47.127846 38.991547
C 49.304581 41.049412 34.549500
C 52.078457 48.427193 33.520622
C 45.906048 51.404835 37.572365
N 45.832962 45.990150 37.027203
C 43.099445 47.238380 40.502834
C 49.030987 39.768280 34.222614
C 53.080105 48.360085 32.616634
C 44.614487 51.714264 36.790703
N 47.710522 44.185726 35.717930
C 42.136932 48.128563 41.265362
C 44.817253 52.502865 35.508286
N 47.483536 48.199459 36.378193
C 45.399223 48.315205 37.645367
C 45.609486 43.554340 36.811771
C 49.852074 44.073582 34.554413
C 49.522411 48.857140 35.179340
C 42.982273 43.959145 38.388790
C 46.461777 40.660191 36.056351
C 52.398041 45.218746 33.191608
C 48.674664 51.731903 35.909908
C 45.087418 46.954609 37.633209
C 46.789639 43.264290 36.120327
C 50.148327 45.435211 34.523991
C 48.372349 49.125385 35.912697
O 48.493706 45.930096 37.652527
O 42.074551 48.116543 42.492214
O 41.410969 48.915249 40.504234
C 43.891758 46.372036 38.239704
C 47.204292 41.923656 35.750202
C 51.312580 45.998062 33.866600
C 47.941635 50.475830 36.270039
O 48.343170 51.085732 40.940178
O 45.708729 53.297737 35.311150
O 43.843216 52.260166 34.615879
C 43.953331 45.027340 37.986992
C 48.423744 42.061481 35.108723
C 51.208790 47.370838 34.009319
C 46.775379 50.332611 36.975262
C 45.182735 44.807068 37.233982
C 48.714344 43.497501 35.115326
C 49.986084 47.598042 34.788097
C 46.510319 48.893742 37.033360
N 49.372555 46.421650 35.066113
Fe 47.599045 46.205269 36.060406
H 42.734806 48.143257 38.582745
H 41.852028 46.652737 38.836868
H 50.333630 41.384647 34.373463
H 51.880390 49.417969 33.943066
H 45.626709 51.117268 38.598900
H 46.484642 52.333466 37.673157
H 44.113659 47.635284 40.683334
H 43.098038 46.251190 40.991398
H 48.035690 39.330952 34.319660
H 49.816013 39.120148 33.821674
H 53.355225 47.440441 32.098003
H 53.647533 49.258652 32.357819
H 44.051716 50.799461 36.556248
H 43.947723 52.336189 37.417557
H 44.709885 48.981548 38.164288
H 44.963669 42.711639 37.060501
H 50.562763 43.404087 34.071293
H 50.113079 49.722225 34.879246

H 42.634369 43.371941 37.520840
H 43.431694 43.241852 39.098267
H 42.093079 44.387714 38.876122
H 45.746895 40.795952 36.881378
H 45.888229 40.289555 35.185760
H 47.156502 39.854198 36.345638
H 53.391403 45.633511 33.431335
H 52.396236 44.163128 33.500919
H 52.302563 45.236725 32.089657
H 49.649887 51.793839 36.423794
H 48.877743 51.781067 34.826763
H 48.086388 52.624157 36.162136
C 47.151104 48.505650 42.312115
O 46.962158 47.052135 40.399200
C 47.240471 48.142250 40.862110
O 49.914116 50.257439 42.340111
C 49.499695 50.987839 41.405468
O 52.403866 56.170761 38.821217
C 50.535831 51.930786 40.766815
O 54.176071 55.233639 39.805248
C 50.268990 52.522709 39.522476
C 51.062710 53.557949 39.041080
C 52.176304 53.988586 39.783508
C 52.515629 53.315228 40.975746
C 51.694836 52.302734 41.463566
C 52.908169 55.213188 39.414848
H 46.803844 47.657726 42.921082
H 46.481510 49.376175 42.417286
H 48.140690 48.887409 42.628445
H 47.593304 48.971340 40.207249
H 48.013199 46.399284 38.354179
H 49.376911 52.191479 38.990185
H 50.813103 54.067242 38.107460
H 53.395794 53.640846 41.534149
H 51.904469 51.800293 42.410156
H 54.971230 47.075871 46.059849
H 54.903297 47.883823 44.479439
H 57.125496 58.144939 44.720966
H 51.338802 60.292576 41.388912
H 50.431114 60.376411 39.856724
H 39.566456 52.118034 45.492245
H 47.336960 41.417259 42.694256
H 48.695705 41.024498 41.612656
H 54.430618 54.585392 35.612190
H 55.565899 55.873646 36.128544
H 56.432392 50.739094 38.111217
H 57.363956 52.142815 37.508224
H 55.598988 49.641357 36.621723
H 54.435886 48.737888 35.632481
H 43.741199 55.618774 30.601746
H 45.777397 48.983681 32.532356
H 44.467503 49.619595 33.568562
H 48.318665 54.627052 38.854359
